# Oxidative Dehydrogenation of *N*‐Heteroaromatic Alkyl Alcohols and Amines Facilitated by Dearomative Tautomerization

**DOI:** 10.1002/chem.202501531

**Published:** 2025-06-04

**Authors:** Omid Ghasemloo, Carson L. Hasselbrink, Douglas D. Cardona, Brenton DeBoef, Dugan Hayes

**Affiliations:** ^1^ Department of Chemistry University of Rhode Island 45 Upper College Rd Kingston RI 02881 USA

**Keywords:** aldehydes, arenes, nitrogen heterocycles, oxidations, tautomerization

## Abstract

The oxidation of alcohols, amines, and halides is a fundamental transformation in organic chemistry with significant applications in the synthesis of fine chemicals, pharmaceuticals, and natural products. Here we show that a broad variety of *N*‐heteroarenes bearing hydroxymethyl, aminomethyl, or halomethyl groups are oxidatively dehydrogenated to their respective aldehydes by simply heating them in acidic or basic aqueous solution under ambient atmosphere. The quantitative oxidation of 9‐acridinemethanol to 9‐acridinecarboxaldehyde serves as an illustrative example, proceeding to completion within 3 hours in refluxing 5% aqueous acetic acid or even household vinegar. Quinoline derivatives may be similarly oxidized but require higher temperatures and longer reaction times, while indole derivatives are oxidized under basic conditions. Based on comprehensive regioselectivity screens, internal kinetic isotope competition, and density functional theory (DFT) calculations, we propose a mechanism in which migration of a methylene hydrogen to the pyridinic nitrogen by acid‐catalyzed dearomative tautomerization yields an unstable enol or enamine intermediate that then irreversibly loses two hydrogen atoms to atmospheric oxygen. In addition to the simplicity and environmentally benign nature of our method, we observe no indication of any over‐oxidation to carboxylic acids. Finally, we demonstrate the synthetic utility of this reaction through two different one‐pot formylations of acridine.

## Graphical abstract

*N*‐heteroaromatic alkyl alcohols and amines are inherently unstable in acidic and/or basic solution due to an irreversible dehydrogenation that proceeds from a minority tautomer. In many cases, this reaction results in quantitative oxidation to the corresponding aldehyde, providing a simple, convenient, and green approach for preparing versatile synthetic building blocks.

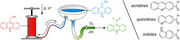

## 1 Introduction

**Scheme 1:**
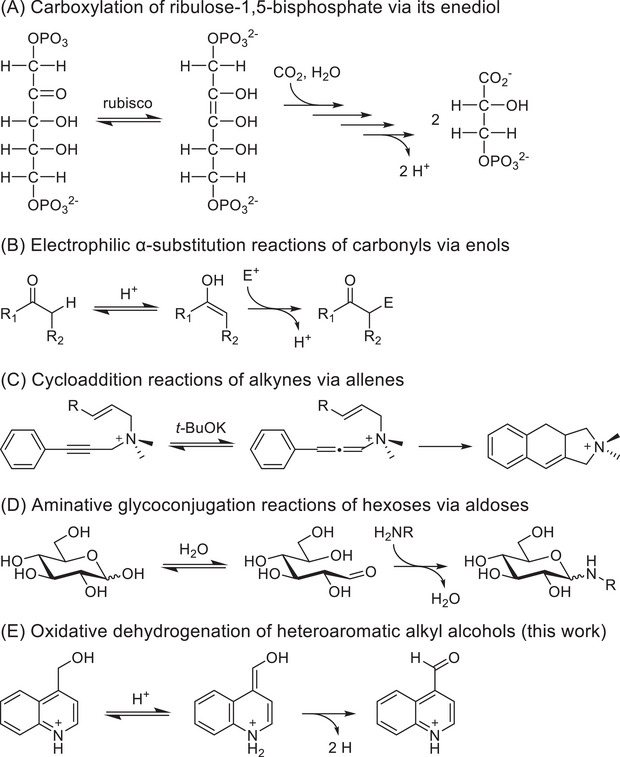
Examples of reactions driven by tautomeric equilibria.

Tautomeric equilibria provide access to thermodynamically unfavorable species that can be continuously siphoned away by irreversible reactions to give quantitative conversion of the starting material. These cascade‐like schemes typically involve addition or substitution reactions of the minority tautomer. Possibly the most important example of such a reaction is the tautomerization of ribulose‐1,5‐bisphosphate to its enediol followed by carboxylation and fragmentation in the Calvin cycle (Scheme 1A),^[^ ^1^, ^2^ ^]^ while the example best known to synthetic chemists is the electrophilic α‐substitution of carbonyls via their respective enols (Scheme 1B). Other examples include cycloaddition reactions of allenes prepared from alkynes (Scheme 1C)^[^ ^3^, ^4^, ^5^ ^]^ and aminative glycoconjugation reactions of aldoses (Scheme 1D).^[^ ^6^, ^7^, ^8^ ^]^ Pairing tautomerization with elimination reactions, however, is a far less common approach, as such a cascade requires that the starting material itself be inherently unstable under the reaction conditions. Accordingly, most examples of such reactions only occur in the gas phase.^[^ ^9^, ^10^ ^]^ Here, however, we demonstrate that tautomerization of *N*‐heteroaromatic alkyl alcohols results in irreversible and selective oxidative dehydrogenation to the corresponding carbonyls (Scheme 1E) without the need for the precious metal catalysts generally used for Guerbet‐type transformations.^[^ ^11^ ^]^ We also show that similar *N*‐heteroaromatic alkylamines can be converted to the corresponding carbonyls in the same fashion, presumably via imino intermediates.

This approach to the oxidation of this class of alcohols offers several benefits over conventional methods: it may be performed in aqueous solution or in organic solvents; it may be performed in air or nearly air‐free environments; it requires only a dilute weak acid (e.g., distilled vinegar) instead of harsh and/or toxic oxidizing agents; it is entirely selective for oxidation of primary alcohols and amines to aldehydes, showing no evidence of over‐oxidation to carboxylic acids; and in many cases, quantitative conversion can be achieved. In comparison, methods employing alkoxides (Oppenauer^[^ ^12^ ^]^ and Oppenauer‐Woodward^[^ ^13^ ^]^ oxidations), periodinane (Dess‐Martin oxidation^[^ ^14^ ^]^), and activated dimethylsulfoxide (Kornblum,^[^ ^15^ ^]^ Pfitzner–Moffatt,^[^ ^16^ ^]^ Albright‐Goldman,^[^ ^17^ ^]^ Parikh–Doering,^[^ ^18^ ^]^ Corey‐Kim,^[^ ^19^ ^]^ and Swern^[^ ^20^ ^]^ oxidations) are incompatible with aqueous conditions, with the latter also generating malodorous dimethyl sulfide; oxoammonium salts (e.g., TEMPO^[^ ^21^ ^]^) require co‐catalysts, often resulting in challenging separations; and hexavalent chromium reagents (pyridinium chlorochromate,^[^ ^22^ ^]^ pyridinium dichromate,^[^ ^23^ ^]^ and Collins reagent^[^ ^24^ ^]^) are exceptionally toxic.

We show that this reaction provides an efficient route to a broad variety of targets, specifically aldehydes of acridines, quinolines, and indoles. Aldehydes are convenient functional handles in applications ranging from bioconjugation^[^ ^25^ ^]^ to assembly of supramolecular systems^[^ ^26^, ^27^ ^]^ and covalent‐organic frameworks,^[^ ^28^ ^]^ and *N*‐heteroarenes are among the most privileged scaffolds in pharmaceutical chemistry.^[^ ^29^ ^]^ Quinoline derivatives in particular are noted for their wide‐ranging pharmacological properties, with the families of cinchona alkaloids (e.g., quinine)^[^ ^30^ ^]^ and topoisomerase inhibitors (e.g., camptothecins)^[^ ^31^ ^]^ underscoring their critical therapeutic roles. But while access to *N*‐heteroaromatic aldehydes is thus highly desirable for the synthesis of small‐molecule drugs and the construction of advanced photoresponsive materials, straightforward and high‐yielding methods for formylation of such electron‐deficient aromatics remain rare.

Recent approaches for preparing *N*‐heterocyclic aldehydes include 1) Kornblum oxidation of bromomethyl derivatives^[^ ^32^ ^]^ and iodomethyl derivatives generated in situ,^[^ ^33^, ^34^ ^]^ 2) oxidation of hydroxymethyl derivatives with pyridinium chlorochromate^[^ ^35^ ^]^ and periodinane;^[^ ^36^ ^]^ 3) oxidation of methyl derivatives with SeO_2_,^[^ ^37^ ^]^ hypervalent iodine,^[^ ^38^ ^]^ and Cu(I)/O_2_;^[^ ^39^ ^]^ and 4) hydrolysis of acetals derived from Minisci‐type reactions.^[^ ^40^, ^41^, ^42^ ^]^ Our method, on the other hand, does not require any toxic reagents or catalysts and proceeds from a variety of easily accessible and shelf‐stable precursors. As an example of the synthetic potential of the reaction, we show that it may be implemented in tandem with photochemical^[^ ^43^ ^]^ and iron‐mediated^[^ ^36^ ^]^ hydroxyalkylations to enable high‐yielding and site‐selective formylations of *N*‐heteroarenes in a single synthetic step.

## 2 Results and Discussion

### 2.1 Oxidation of Hydroxymethylacridines

The work reported herein was prompted by the serendipitous observation that a sample of 9‐acridinemethanol (9‐AcMeOH, **2**) in a dilute solution of trifluoroacetic acid (TFA) in acetonitrile converted quantitatively to 9‐acridinecarboxaldehyde (9‐AcCHO, **3**) under ambient conditions over several days. The same result was observed using water in place of acetonitrile, so all subsequent reactions were performed in aqueous solution. Conditions for the oxidation of 9‐AcMeOH were then optimized with respect to acid identity, acid quantity, temperature, and duration. Percent conversion was determined by comparing the integration of the aldehyde peak in the ^1^H NMR spectrum of the crude reaction mixture to that of the most downfield aromatic peak of the starting material.

Both mineral and organic acids effected conversion at room temperature after 72 hours, with 5% (v/v) aqueous HCl and acetic acid giving 76% and 100%, respectively; lower conversions were observed using only 2% (v/v) acid (Table S1; Figure S13). Notably, household white vinegar (∼5% aqueous acetic acid) afforded 75% conversion, comparable to that achieved using laboratory‐grade acid. Hydrochloric acid consistently gave slower conversion than acetic acid (Figure S14) and showed additional ^1^H NMR peaks in the crude mixture presumably corresponding to chlorinated side products; accordingly, acetic acid was used for all subsequent reactions. As expected, the reaction was greatly accelerated under refluxing conditions, going nearly to completion within only 2 hours (Table S2; Figure S15). While all starting material peaks in the ^1^H NMR spectra vanished after 4 hours under reflux, peaks arising from unidentified side products also became noticeable at that time, giving an optimal duration of 2–4 hours. These conditions are reported as Method A (see Supporting Information).

To explore the scope of this reaction and gain insight into the mechanism, we then attempted the oxidation of other AcMeOH isomers as shown in Table 1 (note the color coding of the positions in the starting material: green, >90% conversion; yellow, 1–90% conversion; red, <1% conversion). While 9‐AcMeOH was readily prepared from commercially available 9‐bromomethylacridine according to the method of Campbell et al.,^[^ ^44^ ^]^ the novel 1‐, 2‐, and 3‐AcMeOH isomers (**5** – **7**) were instead prepared via copper‐catalyzed cross‐coupling of anthranil with the appropriate hydroxymethylphenylboronic acid as described by Wei and coworkers.^[^ ^45^ ^]^ The *meta*‐substituted phenylboronic acid furnishes a mixture of the 1‐ and 3‐substituted products, but the isomers were easily separated by flash chromatography.

**Table 1:** Oxidation of hydroxymethylacridines.

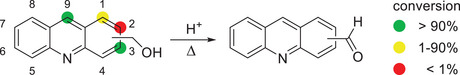				
Entry	Substituent position	Temperature [°C]	Time [hours]	Conversion [%]
1	1	220	9	15
2	2	220	7	<1
3	3	220	4	100
4	9	101	3	100

Marginal conversion was observed for 3‐AcMeOH (**7**) following Method A. However, performing the same reaction at 220 °C in a pressure tube gave quantitative conversion to 3‐AcCHO (**9**) after 4 hours (Figure S20); beyond this time, peaks from side products began to appear in the ^1^H NMR spectrum. These conditions are reported as Method B. The 1‐AcMeOH isomer (**5**) proved more challenging to oxidize, showing 15% conversion after 9 hours at 220 °C alongside an equal amount of an unidentified side product that was present even at the earliest reaction times (Figure S18). No detectable aldehyde peak was observed at all in the reaction mixture of 2‐AcMeOH (**6**) following Method B, even after 7 hours (Figure S19). Overall, this regioselectivity conforms with the mechanism we present in the following section.

### 2.2 Proposed Mechanism

**Scheme 2:**
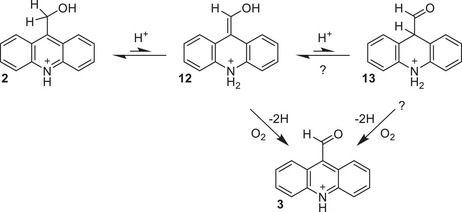
Proposed mechanism for oxidation of 9‐AcMeOH to 9‐AcCHO.

Here, we discuss the mechanism of the oxidation reaction using 9‐AcMeOH as a representative substrate. As illustrated in Scheme 2, we propose that the reaction proceeds first via migration of a methylene hydrogen to the nitrogen atom with concomitant rearrangement of the π‐bond network to give the enolic intermediate **12**. While this could possibly occur through a sigmatropic pathway (i.e., a [1,5] hydride shift), accessing the transition state would necessitate a substantial deformation along the acridine long axis. Additionally, the quantitative oxidation of 2‐QuMeOH (vide infra) demonstrates that the migration must proceed in that case via a solvent‐mediated pathway (i.e., tautomerization), as the [1,3] hydride shift would be strongly disfavored. Though, we cannot conclusively rule out a hydride shift for substrates with the appropriate structures for a [1,5] or [1,7] rearrangement, the example of 2‐QuMeOH and the acceleration of the 9‐AcMeOH oxidation reaction with higher acid concentration both lead us to propose tautomerization as the general pathway for this step. Isomers bearing hydroxymethyl groups at positions that are separated from the nitrogen by an even number of atoms, meanwhile, can undergo neither hydride shift nor tautomerization and are thus unchanged under the reaction conditions.

At this stage, the enol could then undergo a second tautomerization to the carboxaldehyde (**13**) of acridane (dihydroacridine). This intermediate is reminiscent of the reduced form of nicotinamide present in NADH; we emphasize, however, that the conversion of **2** to **13** is simply an isomerization, while the reduction of NAD^+^ to NADH requires the net gain of a hydride. Finally, two hydrogen atom equivalents are lost to give the product; this step could plausibly occur from the enol, the acridane, or both. Gas chromatographic analysis of the reaction headspace shows that the hydrogen atoms are not lost as H_2_. Additionally, conversion of 9‐AcMeOH to 9‐AcCHO after refluxing in 5% (v/v) aqueous acetic acid for 2 hours drops from 95% in air to only 28% in continuously argon‐sparged solution (Figure S35). Together, these experiments suggest that O_2_ acts as the terminal hydrogen acceptor, though it remains unclear whether this process occurs in a concerted or multi‐step fashion or whether the hydrogens are lost as two hydrogen atoms or as a proton/hydride pair. We note that while the presence of O_2_ substantially accelerates the reaction, it still proceeds at a respectable rate under nearly anaerobic conditions, and no further acceleration of the reaction is observed under a pure O_2_ atmosphere versus air. Although we cannot conclusively state whether O_2_ is obligatory for the reaction with only the data presented here, the loss of two hydrogen atoms to O_2_ during the oxidation step clearly outcompetes other possible pathways (e.g., loss to another hydrogen atom acceptor or elimination as H_2_), even at very low O_2_ concentrations.

Though we have referred to the initial intermediate in our mechanism as an enol to highlight the possibility of a subsequent keto‐enol tautomerization, we stress here that the initial tautomerization step is in fact an imine‐enamine tautomerization occurring across the pyridine ring. Pyridinic enamines have indeed been invoked as reactive intermediates in the proposed mechanisms for a variety of functionalization reactions of 2‐ and 4‐methyl *N*‐heteroarenes, including copper‐catalyzed aminations,^[^ ^46^ ^]^ amidations,^[^ ^47^ ^]^ aminoalkylations,^[^ ^48^ ^]^ and esterifications,^[^ ^49^ ^]^ acid‐catalyzed halogenations,^[^ ^33^, ^34^ ^]^ sulfonations,^[^ ^50^ ^]^ and deuterations,^[^ ^51^ ^]^ and the syntheses of thiazoles^[^ ^52^ ^]^ and thiadiazoles,^[^ ^53^ ^]^ providing strong precedent for the mechanism shown in Scheme 2.

### 2.3 Oxidation of Hydroxymethyquinolines

We next investigated the reactivity and regioselectivity of the hydroxymethylquinolines (QuMeOH), all but one of which are commercially available. In line with our expectations, 4‐QuMeOH showed only marginal conversion at room temperature after four days or at 160 °C after three days, while 18% conversion was achieved at 200 °C after 24 hours (Table S3; Figure S16). Quantitative conversion to 4‐QuCHO (**11**) was achieved at 220 °C (Method B) after 7 hours (Figure S23), with unidentified side products beginning to accumulate at later times.

Among the other isomers bearing the hydroxymethyl group on the pyridine ring, 2‐QuMeOH was also quantitatively oxidized to 2‐QuCHO (**10**) following Method B within 7 hours (Figure S21), while 3‐QuMeOH showed only a negligible aldehyde peak under the same conditions (Figure S22). This result is again in excellent agreement with our proposed mechanism. Additionally, the three distal isomers (5‐, 6‐, and 7‐QuMeOH) all showed a new aldehyde peak after 7 hours, but conversion remained under 1% for each (Figures S24–S26). These results are summarized in Table 2.

**Table 2:** Oxidation of hydroxymethylquinolines.

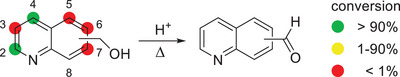				
Entry	Substituent position	Temperature [°C]	Time [hours]	Conversion [%]
1	2	220	4	100
2	3	220	7	<1
3	4	220	7	100
4	5	220	7	<1
5	6	220	7	<1
6	7	220	7	<1

### 2.4 Oxidation of Hydroxymethylindoles

**Scheme 3:**
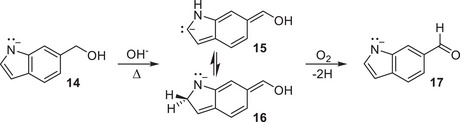
Proposed mechanism for oxidation of 6‐InMeOH to 6‐InCHO.

To explore the scope of this reaction beyond pyridinic heteroarenes, we turned to the hydroxymethylindoles (InMeOH). The oxidation reaction showed negligible conversion under the conditions of Method B with 5% aqueous acetic or hydrochloric acid, but we hypothesized that it should instead proceed under basic conditions with indoles such that the deprotonated nitrogen has a lone pair. As shown for 6‐InMeOH (**14**) in Scheme 3, we propose that proton (rather than hydride) migration occurs from the methylene carbon to the heteroatom, giving an enolic intermediate with a formal carbanion (**15**) that can then exchange a proton with the neighboring nitrogen (**16**). Finally, loss of two hydrogen atoms to O_2_ gives the 6‐InCHO product (**17**). Notably, the structure of the indole ring allows a methylene proton migration from any position, and thus we did not expect to observe any regioselectivity. Indeed, heating any of the three commercially available InMeOH isomers in 5% (w/v) aqueous sodium hydroxide at 220 °C results in substantial conversion after 7 hours (Figures S27 – S29), as summarized in Table 3. These conditions are reported as Method C. Optimizing the conditions for these reactions to improve the yield is the subject of ongoing work.

**Table 3:** Oxidation of hydroxymethylindoles.

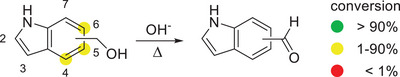				
Entry	Substituent position	Temperature [°C]	Time [hours]	Conversion [%]
1	4	220	7	18
2	5	220	7	44
3	6	220	7	34

### 2.5 Oxidation of Primary Amines

Given the apparent generality of the oxidation reaction among various *N*‐heteroarenes, we then aimed to explore the scope of the reaction with respect to the substituent. First, we hypothesized that other highly reduced substituents with terminal exchangeable protons, such as aminoalkyl groups, could work as effectively as hydroxyalkyl groups. Because oxidation of an aminomethyl group via the mechanism described above would yield an imine that would then rapidly hydrolyze in refluxing aqueous acetic acid, we expected to obtain the same aldehyde product that we would have obtained from the corresponding alcohol. In this sense, the reaction would be comparable to methods employing hypervalent iodine reagents to achieve the same result.^[^ ^54^, ^55^ ^]^

As both 9‐AcMeOH and 4‐QuMeOH were found to undergo facile, quantitative conversion, we chose to attempt aminomethyl oxidation using those scaffolds. 9‐Aminomethylacridine (9‐AcMeNH_2_, **8**) was prepared from 9‐bromomethylacridine and hexamethylenetetramine via Delépine reaction,^[^ ^56^ ^]^ and 4‐aminomethylquinoline (4‐QuMeNH_2_) was obtained commercially. Surprisingly, quantitative conversion of 9‐AcMeNH_2_ to 9‐AcCHO using Method A was achieved in only 1 hour (Table 4; Figures S17, S30), less than half the time required for 9‐AcMeOH; quantitative conversion of 4‐QuMeNH_2_ to 4‐QuCHO using Method B was achieved in 7 hours (Figure S31). From these examples, we can conclude that appropriately aminomethylated *N*‐heteroarenes may also be oxidized to their respective aldehydes using this approach, in some cases even more rapidly than the corresponding hydroxymethylated substrates.

**Table 4:** Oxidation of aminomethylated *N*‐heteroarenes.

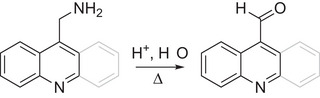				
Entry	Substrate	Temperature [°C]	Time [minutes]	Conversion [%]
1	9‐AcMeNH_2_	101	15	62
2	9‐AcMeNH_2_	101	30	84
3	9‐AcMeNH_2_	101	45	90
4	9‐AcMeNH_2_	101	60	100
5	4‐QuMeNH_2_	220	420	100

**Scheme 4:**
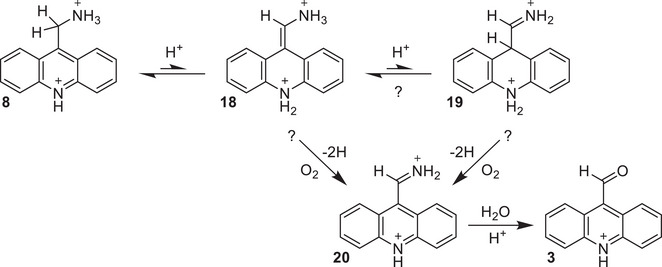
Proposed mechanism for oxidation of 9‐AcMeNH_2_ to 9‐AcCHO.

Attempts to observe the putative iminoacridine intermediate shown in Scheme 4 (**20**) by performing the reaction in anhydrous acetonitrile‐*d*_3_ in a sealed J. Young tube proved unsuccessful, as only the aldehyde product was observed. Assuming, however, that the same reaction mechanism is operative for both the aminomethylated and hydroxymethylated substrates, we again cannot conclusively determine whether hydrogen loss occurs from the corresponding enamine (**18**) and/or iminoacridane (**19**) intermediate(s).

The mechanism proposed here is somewhat reminiscent of the so‐called *tert*‐amino effect that underlies the Meth‐Cohn and Reinhoudt reactions, i.e., the cascade [1,5] hydride shift/cyclization of *ortho*‐α,β‐unsaturated *N*,*N*‐dialkylanilines.^[^ ^57^, ^58^, ^59^, ^60^ ^]^ The reactions demonstrated in the current work are distinct from those, however, in that they proceed via migration of a hydride adjacent to an alcohol or primary amine, as these groups in turn enable the hydrogen elimination step(s) that render the reaction irreversible. Accordingly, we expect that secondary aminoalkyl *N*‐heteroarenes may prove even more reactive toward oxidation in this manner, and we are currently exploring this possibility.

### 2.6 Oxidation of Haloalkyl *N*‐Heteroarenes

Our gram‐scale preparation of 9‐AcMeOH from 9‐bromomethylacridine (9‐AcMeBr) proceeds first by substitution of bromide with acetate, which is then followed by hydrolysis under basic conditions. Accordingly, we speculated that 9‐AcCHO could be obtained in a single synthetic step from 9‐AcMeBr by Method A, during which it progresses through the acetoxy and hydroxy intermediates to give the product. Indeed, we found that refluxing 9‐AcMeBr for 5 hours under the conditions of Method A affords 9‐AcCHO in decent isolated yield (26%; Figure S33). The ^1^H NMR spectrum of the crude product mixture showed roughly equal amounts of 9‐AcCHO and the dehalogenation side product (9‐methylacridine) and a small amount of the acetoxy intermediate but no residual starting material (Figure S32); longer reaction times resulted in diminished yield due to the formation of unidentified side products. This reaction achieves the same transformation as the Kornblum/Ganem oxidation^[^ ^61^ ^]^ but is compatible with aqueous conditions and does not require the use of DMSO and/or *N*‐oxides.

### 2.7 Isotope Labeling Studies

Beyond the regioselectivity studies described above, we sought to investigate the mechanism of the acid‐mediated reaction through an intramolecular competition at the methylene position using 9‐AcMeOH‐*d* (**4**) prepared from 9‐AcCHO with NaBD_4_. The ^1^H NMR spectrum of the product mixture shows the expected 2H aromatic peaks for the product and a substantially diminished aldehyde peak that integrates to 0.15 (Figure S34). Accordingly, we conclude that the branching between the C‐H and C‐D bond cleavage pathways is 85:15, giving a kinetic isotope effect (KIE) of 5.7. This value strongly suggests that C‐H bond cleavage at the methylene position is the rate‐determining step in this reaction, which is consistent with the proposed mechanism.

### 2.8 Computational Results

**Figure 1:**
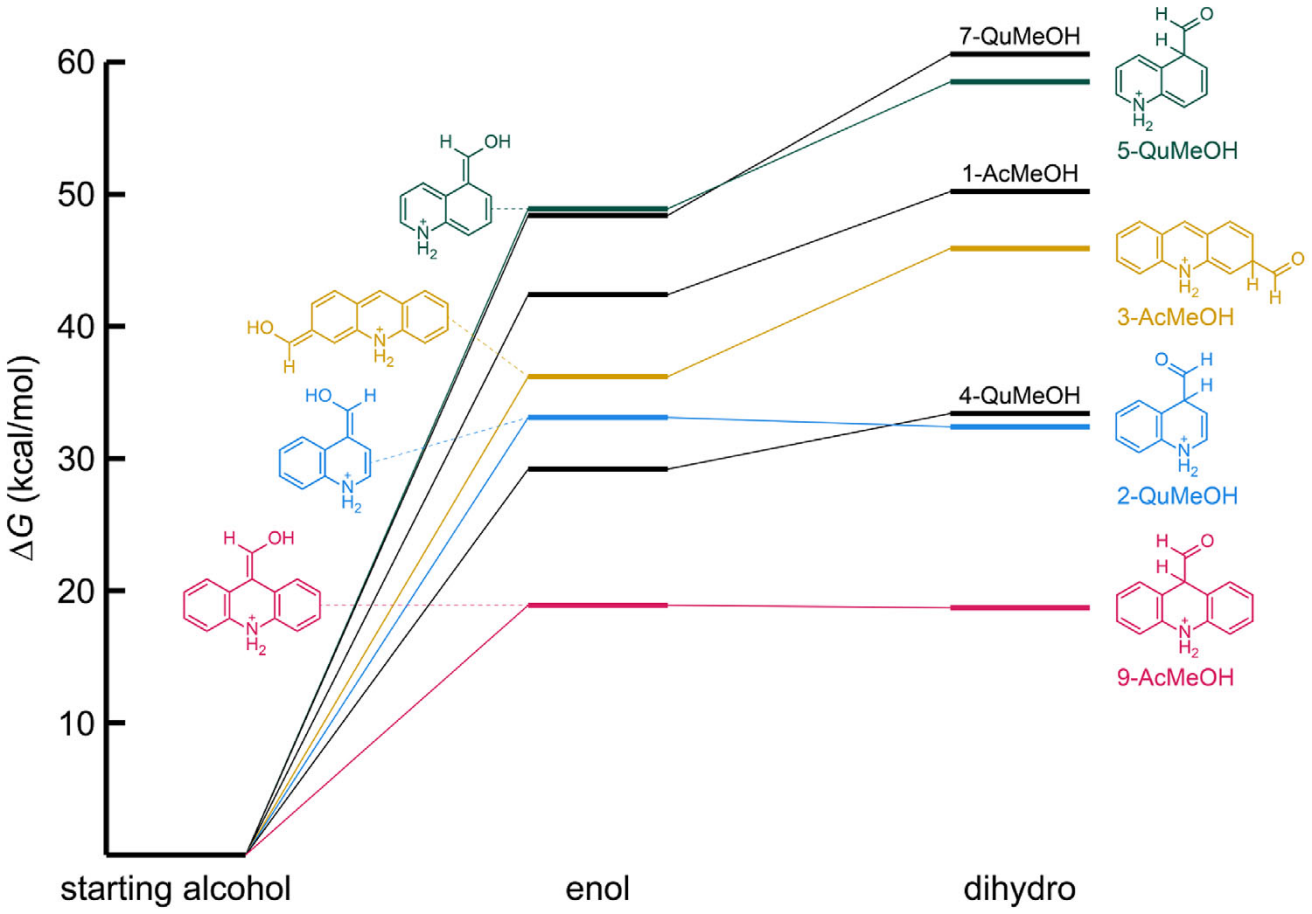
Energy level diagram showing Δ*G* values for enol and dihydroarene carboxaldehyde tautomers of AcMeOH and QuMeOH isomers calculated by DFT. Structures of two representative isomers of each are shown with color‐coded energy levels to illustrate how aromaticity is broken in the pyridine or benzene ring; the other isomers are labeled above their energy levels on the right‐hand side.

Additional evidence in support of the initial rearrangement to the enolic intermediate as the rate‐limiting step comes from comparing the temperature dependence and yields observed for the different substrates to the Δ*G* values calculated for the tautomerization reactions by DFT using the GAMESS 2022.1 package.^[^ ^62^ ^]^ While 9‐AcMeOH oxidizes rapidly at 100 °C, 3‐AcMeOH requires a temperature of 220 °C to reach completion. Qualitatively, this observation is consistent with the greater loss of aromaticity involved in producing the enol tautomer of 3‐AcMeOH (i.e., the aromaticity of benzene vs. pyridine). Our DFT calculations, summarized in Table 5 and Figure 1, indeed show a Δ*G* value for the formation of the 3‐AcMeOH enol that is 17.3 kcal/mol higher than that of 9‐AcMeOH. The Δ*G* value for 1‐AcMeOH is 6.2 kcal/mol higher still, in agreement with the substantially lower conversion observed for that substrate (15%) at 220 °C.

**Table 5:** Relative free energies of proposed intermediates calculated by DFT.^[a]^

Entry	Starting alcohol	Δ*G*, Enol [kcal/mol]	Δ*G*, dihydro [kcal/mol]
1	1‐AcMeOH	42.4	50.2
2	3‐AcMeOH	36.2	45.9
3	9‐AcMeOH	18.9	18.7
4	2‐QuMeOH	33.1	32.4
5	4‐QuMeOH	29.2	33.4
6	5‐QuMeOH	48.9	58.5
7	7‐QuMeOH	48.4	60.6

^[a]^ 6–311++G**/B3LYP/PCM(H_2_O); values calculated for *N*‐protonated cations.

Once again invoking aromaticity, the quinolines bearing the substituent on the pyridine ring should show Δ*G* values between those of 9‐ and 3‐AcMeOH, while those bearing the substituent on the benzene ring should show the highest Δ*G* values of all. This is borne out by experiment as well, with 2‐ and 4‐QuMeOH showing complete conversion at 220 °C and 5‐ and 7‐QuMeOH showing only trace amounts of aldehyde. The DFT results are also consistent, showing nearly equivalent Δ*G* values for 2‐ and 4‐QuMeOH and for 5‐ and 7‐QuMeOH, respectively lying 10.5 and 28.6 kcal/mol above that of 9‐AcMeOH.

DFT calculations also provide another perspective for considering the possible involvement of the dihydroarene carboxaldehyde tautomers discussed earlier and depicted on the right‐hand side of Figure 1. For 9‐AcMeOH and 2‐QuMeOH, this tautomer is nearly isoenergetic with the corresponding enol. Thus, it is reasonable to expect similar concentrations of both tautomers in solution during the oxidation of those substrates. This scenario is less likely but still not impossible for 4‐QuMeOH, where the dihydroarene carboxaldehyde is only 4.2 kcal/mol higher than the enol. However, the Δ*G* values for the dihydroarene carboxaldehydes of 1‐ and 3‐AcMeOH are > 45 kcal/mol, demonstrating that these species do not form, even at 220 °C. Since the reaction proceeds to some degree for both of those substrates, we can conclude that the aldehyde forms from the enol tautomer in all cases, though we cannot rule out the possibility that it also forms from the dihydroarene carboxaldehyde tautomer for 9‐AcMeOH and 2‐ and 4‐QuMeOH.

Finally, we note that we did not endeavor to find transition states for these reactions, and the values reported here correspond to Δ*G* rather than Δ*G* ^‡^. Nevertheless, the trends predicted by the DFT calculations are in excellent agreement with our experimental observations and thus provide strong support for our proposed mechanism.

### 2.9 One‐Pot Formylation of Acridine

**Scheme 5:**
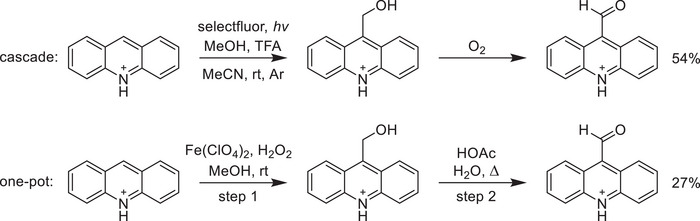
(Top) Cascade synthesis of 9‐AcCHO from acridine and methanol and corresponding isolated yield. (Bottom) One‐pot synthesis affording the same net transformation.

The simplicity of the reaction conditions employed here makes this method broadly compatible with other reactions for one‐pot syntheses. For example, the aldehyde generated in situ can be used to couple the heteroarene to a desired amine through an A^3^ coupling reaction^[^ ^63^ ^]^ or a reductive amination. Here we instead show that two different syntheses of 9‐AcMeOH can be followed directly by oxidative dehydrogenation to give 9‐AcCHO in good yield, as illustrated in Scheme 5.

Our first preparation of 9‐AcCHO follows the selectfluor‐mediated photochemical coupling of alcohols and *N*‐heteroarenes reported by Niu et al.^[^ ^43^ ^]^ That reaction includes TFA in excess, which we expected could also serve to drive the oxidation of the resulting alcohol in a cascade scheme. Though no discussion of aldehyde side products appears in that report, we believe that is likely due to the inert atmosphere and room temperature reaction conditions and a substrate scope limited to quinolines and isoquinolines. As demonstrated here, the acid‐catalyzed oxidation is O_2_‐dependent and only occurs at elevated temperatures for quinolines. But because 9‐AcMeOH oxidizes at room temperature in the presence of TFA, we expected that the photochemical coupling of acridine and methanol would give 9‐AcCHO directly if sufficient O_2_ were present. The exclusion of air during the photochemical step is even more crucial with acridine as a substrate, however, as acridine absorbs blue light and reacts in its excited state with O_2_ to give acridone. Accordingly, we performed the photochemical step under argon before stirring in the dark under air for an additional 24 hours. The product mixture (Figure S36) contained 18% unreacted acridine and 82% 9‐AcCHO (isolated in 54% yield) with only trace amounts of 9‐AcMeOH and acridone, demonstrating that the cascade synthesis was indeed successful.

Our second preparation follows the iron‐mediated synthesis of 2‐ and 4‐QuMeOH derivatives reported by Shantharjun et al.^[^ ^36^ ^]^ This reaction proceeds in air but without the addition of acid, and thus we opted for a one‐pot, two‐step approach: following the hydroxymethylation step, the reaction mixture was diluted with 5% aqueous acetic acid and stirred under reflux for 3 hours to drive the oxidation. Once again, the product mixture contained only unreacted acridine (60%) and 9‐AcCHO (40%), which was isolated in 27% yield (Figure S37).

## 3 Conclusion

We have shown that appropriately positioned hydroxyalkyl and aminoalkyl groups in a broad variety of *N*‐heteroarenes are inherently unstable under acidic or basic conditions, undergoing irreversible oxidative dehydrogenation to their respective aldehydes. Similar transformations may be achieved for substrates bearing haloalkyl groups as well, presumably via hydroxyalkyl intermediates. The scope of this reaction includes acridines, quinolines, and indoles, and we are currently exploring the suitability of nonaromatic heterocycles and even acyclic substrates. A combination of regioselectivity screens, KIE measurements, and DFT calculations suggests a mechanism in which aromaticity is broken in an initial tautomerization step to give an enol and/or dihydroarene carboxaldehyde intermediate that ultimately loses two hydrogen atom equivalents to O_2_. Our method is simple, selective, sustainable, and nontoxic, requiring only heat and a dilute acid or base and showing no evidence of over‐oxidation to carboxylic acids. Given the remarkably minimal nature of the reaction, we believe that it was likely “hiding in plain sight” – that is, many such transformations reported in the literature employing conventional oxidation reagents may have proceeded without the aid of those reagents at all. We expect this approach to reduce the environmental burden and improve the scalability of the synthesis of key intermediates in applications ranging from pharmaceuticals to advanced functional materials.

## Conflict of Interests

The authors declare no conflict of interest.

## Supporting information



Supporting Information

## Data Availability

The data that support the findings of this study are available from the corresponding author upon reasonable request.

## References

[1] J. Bassham , A. Benson , L. D. Kay , A. Z. Harris , A. T. Wilson , M. Calvin , J. Biol. Chem. 1950, 185, 781. 14774424

[2] A. Jaworowski , I. A. Rose , J. Biol. Chem. 1985, 260, 944. 3918036

[3] T. Laird , W. D. Ollis , J. Chem. Soc., Chem. Commun. 1972, 557.

[4] X. You , X. Xie , H. Chen , Y. Li , Y. Liu , Chem. Euro. J. 2015, 21, 18699. 10.1002/chem.201503374 26558737

[5] N. Kraemer , R. R. Naredla , T. R. Hoye , Org. Lett. 2022, 24, 2327. 35311283 10.1021/acs.orglett.2c00491 PMC9050169

[6] E. Mitts , R. M. Hixon , J. Am. Chem. Soc. 1944, 66, 483.

[7] J. W. Haas , R. E. Kadunce , J. Am. Chem. Soc. 1962, 84, 4910.

[8] M. A. Rapp , O. R. Baudendistel , U. E. Steiner , V. Wittmann , Chem. Sci. 2021 12, 14901. 34820106 10.1039/d1sc05008g PMC8597863

[9] L. N. Heydorn , L. M. Carter , R. D. Bowen , J. K. Terlouw , Eur J Mass Spectrom (Chichester) 2003, 9, 343. 12939486 10.1255/ejms.550

[10] R. R. Wu , M. T. Rodgers , Phys. Chem. Chem. Phys. 2016, 18, 24451. 27536972 10.1039/c6cp03620a

[11] G. E. Dobereiner , R. H. Crabtree , Chem. Rev. 2010, 110, 681. 19938813 10.1021/cr900202j

[12] R. V. Oppenauer , Recueil des Travaux Chimiques des Pays‐Bas 1937, Vol. 56, p. 137.

[13] R. B. Woodward , N. L. Wendler , F. J. Brutschy , J. Am. Chem. Soc. 1945, 67, 1425.

[14] D. B. Dess , J. C. Martin , J. Org. Chem. 1983, 48, 4155.

[15] N. Kornblum , W. J. Jones , G. J. Anderson , J. Am. Chem. Soc. 1959, 81, 4113.

[16] K. E. Pfitzner , J. G. Moffatt , J. Am. Chem. Soc. 1963, 85, 3027.

[17] J. D. Albright , L. Goldman , J. Am. Chem. Soc. 1967, 89, 2416.

[18] J. R. Parikh , W. v. E. Doering , J. Am. Chem. Soc. 1967, 89, 5505.

[19] E. J. Corey , C. U. Kim , J. Am. Chem. Soc. 1972, 94, 7586.

[20] K. Omura , D. Swern , Tetrahedron 1978, 34, 1651.

[21] N. Merbouh , J. M. Bobbitt , C. Brückner , Org. Prep. Proced. Int. 2004, 36, 1, 10.1080/00304940409355369.

[22] E. J. Corey , J. W. Suggs , Tetrahedron Lett. 1975, 16, 2647.

[23] E. J. Corey , G. Schmidt , Tetrahedron Lett. 1979, 20, 399.

[24] J. C. Collins , W. W. Hess , F. J. Frank , Tetrahedron Lett. 1968, 9, 3363.

[25] G. R. Gray , Arch. Biochem. Biophys. 1974, 163, 426. 4859528 10.1016/0003-9861(74)90495-0

[26] I. Alfonso , M. Bolte , M. Bru , M. I. Burguete , S. V. Luis , J. Rubio , J. Am. Chem. Soc. 2008, 130, 6137. 18402442 10.1021/ja710132c

[27] L. Gabrielli , C. A. Hunter , Chem. Sci. 2020, 11, 7408. 34123021 10.1039/d0sc02234a PMC8159439

[28] F. J. Uribe‐Romo , J. R. Hunt , H. Furukawa , C. Klöck , M. O'Keeffe , O. M. Yaghi , J. Am. Chem. Soc. 2009, 131, 4570. 19281246 10.1021/ja8096256

[29] E. Vitaku , D. T. Smith , J. T. Njardarson , J. Med. Chem. 2014, 57, 10257. 25255204 10.1021/jm501100b

[30] T. S. Kaufman , E. A. Rúveda , Angew. Chem., Int. Ed. 2005, 44, 854. 10.1002/anie.200400663 15669029

[31] V. J. Venditto , E. E. Simanek , Mol. Pharmaceutics 2010, 7, 307. 10.1021/mp900243b PMC3733266 20108971

[32] N. Liu , H. Zhong , J. Tu , Z. Jiang , Y. Jiang , Y. Jiang , Y. Jiang , J. Li , W. Zhang , Y. Wang , C. Sheng , Eur. J. Med. Chem. 2018, 143, 1510. 29126739 10.1016/j.ejmech.2017.10.043

[33] R. Ye , Y. Cao , X. Xi , L. Liu , T. Chen , Org. Biomol. Chem. 2019, 17, 4220. 30946414 10.1039/c9ob00490d

[34] M. L. Tedder , F. O. Dzeagu , M. M. Mason , D. A. Dixon , J. D. Carrick , Tetrahedron 2022, 116, 132805.

[35] Q. Mahmood , E. Yue , W. Zhang , G. A. Solan , T. Liang , W.‐H. Sun , Org. Chem. Front. 2016, 3, 1668.

[36] B. Shantharjun , D. Vani , R. Unnava , M. Sandeep , K. Rajender Reddy , Org. Biomol. Chem. 2021, 19, 645. 33393550 10.1039/d0ob02212h

[37] D. N. Bobrov , V. I. Tyvorskii , Tetrahedron 2010, 66, 5432.

[38] J. Xu , Y. Li , T. Ding , H. Guo , Chem. Asian J. 2021, 16, 3114. 34472705 10.1002/asia.202100704

[39] G. Zheng , H. Liu , M. Wang , Chin. J. Chem. 2016, 34, 519.

[40] W. Jia , Y. Jian , B. Huang , C. Yang , W. Xia , Synlett 2018, 29, 1881.

[41] J. Dong , X. Wang , H. Song , Y. Liu , Q. Dong , Adv. Synth. Catal. 2020, 362, 2155.

[42] W. Wang , Z. Chen , Y. Zhang , W. Zeng , S. Wang , Tetrahedron 2022, 105, 132607.

[43] L. Niu , J. Liu , X.‐A. Liang , S. Wang , A. Lei , Nat. Commun. 2019, 10, 467. 30692540 10.1038/s41467-019-08413-9 PMC6349847

[44] A. Campbell , C. S. Franklin , E. N. Morgan , D. J. Tivey , J. Chem. Soc. 1958, 1145.

[45] Y. Li , L. Xu , Y. Wei , Org. Biomol. Chem. 2022, 20, 9742. 36441231 10.1039/d2ob01705a

[46] Q. Li , Y. Huang , T. Chen , Y. Zhou , Q. Xu , S.‐F. Yin , L.‐B. Han , Org. Lett. 2014, 16, 3672. 24977337 10.1021/ol501454j

[47] H. Xie , Y. Liao , S. Chen , Y. Chen , G.‐J. Deng , Org. Biomol. Chem. 2015, 13, 6944. 26053552 10.1039/c5ob00915d

[48] M. Rueping , N. Tolstoluzhsky , Org. Lett. 2011, 13, 1095. 21288008 10.1021/ol103150g

[49] M. Liu , T. Chen , S.‐F. Yin , Catal. Sci. Technol. 2016, 6, 690.

[50] F. Xiao , S. Chen , Y. Chen , H. Huang , G.‐J. Deng , Chem. Commun. 2014, 51, 652. 10.1039/c4cc07546c 25415851

[51] M. Liu , X. Chen , T. Chen , S.‐F. Yin , Org. Biomol. Chem. 2017, 15, 2507. 28266672 10.1039/c7ob00062f

[52] T. B. Nguyen , L. Ermolenko , A. Al‐Mourabit , Org. Lett. 2013, 15, 4218. 23924277 10.1021/ol401944a

[53] H. Xie , J. Cai , Z. Wang , H. Huang , G.‐J. Deng , Org. Lett. 2016, 18, 2196. 27057761 10.1021/acs.orglett.6b00806

[54] K. C. Nicolaou , C. J. N. Mathison , T. Montagnon , J. Am. Chem. Soc. 2004, 126, 5192. 15099102 10.1021/ja0400382

[55] S. J. Desjardins Guillaume ; S. Canesi, , Synlett 2012, 23, 1497.

[56] F. Camerel , B. Donnio , C. Bourgogne , M. Schmutz , D. Guillon , P. Davidson , R. Ziessel , Chem. Euro. J. 2006, 12, 4261. 10.1002/chem.200501431 16575933

[57] O. Meth‐Cohn , H. Suschitzky , in Advances in Heterocyclic Chemistry (Eds.: A.R. Katritzky , A.J. Boulton ), Academic Press, 1972, pp. 211.

[58] O. Meth‐Cohn , in Advances in Heterocyclic Chemistry (Ed.: A.R. Katritzky ), Academic Press, 1996, pp. 1.

[59] W. Verboom , D. N. Reinhoudt , R. Visser , S. Harkema , J. Org. Chem. 1984, 49, 269.

[60] W. Verboom , D. N. Reinhoudt , Recueil des Travaux Chimiques des Pays‐Bas 1990, 109, 311.

[61] A. G. Godfrey , B. Ganem , Tetrahedron Lett. 1990, 31, 4825.

[62] G. M. J. Barca , C. Bertoni , L. Carrington , D. Datta , N. De Silva , J. E. Deustua , D. G. Fedorov , J. R. Gour , A. O. Gunina , E. Guidez , T. Harville , S. Irle , J. Ivanic , K. Kowalski , S. S. Leang , H. Li , W. Li , J. J. Lutz , I. Magoulas , J. Mato , V. Mironov , H. Nakata , B. Q. Pham , P. Piecuch , D. Poole , S. R. Pruitt , A. P. Rendell , L. B. Roskop , K. Ruedenberg , T. Sattasathuchana , M. W. Schmidt , J. Shen , L. Slipchenko , M. Sosonkina , V. Sundriyal , A. Tiwari , J. L. Galvez Vallejo , B. Westheimer , M. Włoch , P. Xu , F. Zahariev , M. S. Gordon , J. Chem. Phys. 2020, 152, 154102. 32321259 10.1063/5.0005188

[63] C. Wei , C.‐J. Li , J. Am. Chem. Soc. 2003, 125, 9584. 12904013 10.1021/ja0359299

