# Supplementary material for: Oxidative Dehydrogenation of N‐Heteroaromatic Alkyl Alcohols and Amines Facilitated by Dearomative Tautomerization
Source: Chemistry. 2025 Jun 4;31(39):e202501531. doi: 10.1002/chem.202501531 (PMC12258688; doi:10.1002/chem.202501531)
Supplement: Supplementary file 1 — Supporting Information [file CHEM-31-e202501531-s001.pdf]

# Supporting Information

## Oxidative Dehydrogenation of *N*-Heteroaromatic Alkyl Alcohols and Amines Facilitated by Dearomative Tautomerization

Omid Ghasemloo, Carson L. Hasselbrink, Douglas D. Cardona, Brenton DeBoef, and Dugan Hayes*

Department of Chemistry, University of Rhode Island, Kingston, Rhode Island 02881, USA.

*Corresponding author email: dugan@uri.edu

## Table of Contents

**General methods** .......... S02 – S03

**Synthesis and characterization** .......... S04 – S16

**Optimization of reaction conditions** .......... S17 – S23

**Additional oxidation reactions** .......... S24 – S48

**Optimized geometry coordinates** .......... S49 – S69

## General methods

All chemicals were used as received unless otherwise noted. 9-Bromomethylacridine; 2-, 3-, 5-, 6-, and 7-hydroxymethylquinoline; 4-, 5-, and 6-hydroxymethylindole; 3-, and 4-(hydroxy-methyl)phenylboronic acid; and 4-aminomethylquinoline; and 1,2-benzisosoxazole were obtained from Ambeed. 4-Hydroxymethylquinoline was obtained from Synthonix. Acridine, sodium borohydride, sodium borodeuteride (99% *d*), and copper(II) nitrate hydrate were purchased from Thermo Scientific Chemicals. Hexamethylenetetramine and selectfluor were obtained from TCI. Absolute ethanol was purchased from Pharmco. Reagent grade acetonitrile, methanol, ethyl acetate, diethyl ether, dichloromethane (DCM), dimethylformamide (DMF), glacial acetic acid (HOAc), concentrated hydrochloric acid, sodium hydroxide, and 30% hydrogen peroxide solution were obtained from Fisher Scientific. Iron(II) perchlorate hydrate was obtained from Alfa Aesar. Trifluoroacetic acid was obtained from Acros Organics. Chloroform-*d* (99.8% *d*) and dimethyl sulfoxide-$d_6$ (99.8% *d*) were obtained from Sigma Aldrich. Acetonitrile-$d_3$ (99.8% *d*) was obtained from Cambridge Isotope Laboratories. Research grade argon (99.9997%, 0.2 ppm $O_2$) and oxygen (99.999%) were obtained from Airgas.

NMR spectra were recorded using a Bruker Avance III HD spectrometer (400 MHz). Mass spectrometry (MS) was conducted using a Shimadzu LCMS-2020 system. Chemical shifts (δ) are expressed in ppm, and coupling constants ($J$) are reported in Hz. $^1$H and $^{13}$C NMR spectra are referenced internally to residual solvent peaks. $^1$H and $^{13}$C NMR spectra and MS data are reported for novel compounds; $^1$H NMR and MS only are reported for known compounds and matched to literature values.

Percent conversion values were determined from $^1$H NMR spectra by comparing the integration of the aldehyde peak to that of one of the starting material aromatic peaks that had no spectral overlap with the product aromatic peaks. All such NMR samples were prepared by taking an aliquot of the reaction, neutralizing the solution with aqueous NaOH or HCl as appropriate, extracting into DCM, removing solvent under reduced pressure, and drying under vacuum before dissolving in $CDCl_3$.

For reactions under argon and oxygen, the solvent was thoroughly degassed and sparged with the appropriate gas, and the headspace of refluxing assembly was thoroughly purged. Additionally, the

appropriate gas was continuously bubbled through the reaction solution throughout the course of the reaction at a flow rate of 500 mL/min after first bubbling through a separate vessel filled with solvent to saturate the gas and thereby mitigate solvent evaporation in the reaction vessel. The reactions otherwise followed Method A.

All density functional theory calculations were performed using the GAMESS 2022.1 package[61] using the 6-311++G** basis set and the B3LYP functional. Implicit solvent (water) was included using the polarizable continuum model, and all species were modeled as their *N*-protonated cations. Gibbs free energies were calculated following geometry optimization, and values for reaction intermediates are reported relative to their respective starting materials.

**Method A:** The starting material was prepared as a 5 mM solution in 5% (v/v) aqueous acetic or hydrochloric acid. A portion of the solution (20 mL) was refluxed in a 50 mL round-bottom flask for several hours. Upon completion, the reaction mixture was allowed to cool to room temperature and subsequently neutralized with aqueous sodium hydroxide. The mixture was extracted with DCM (3x20 mL). The combined organic layers were washed with brine (20 mL), dried over sodium sulfate, filtered, and concentrated under reduced pressure to yield the crude product. This method was used for 9-hydroxymethylacridine and 9-aminomethylacridine.

**Method B:** A 20 mL solution of the same composition as in Method A was prepared and stirred in a 40 mL pressure tube at 220°C on an oil bath for several hours. The workup then followed the procedure described in Method A. This method was used for 1-, 2-, and 3-hydroxymethylacridine, 4-aminomethylquinoline, and all hydroxymethylquinoline isomers.

**Method C:** The starting material was prepared as a 5 mM solution in 5% (w/v) aqueous sodium hydroxide. A portion of the solution (20 mL) was stirred in a 40 mL pressure tube at 220°C on an oil bath for several hours. The cooled, alkaline reaction mixture was then extracted with DCM (3x20 mL). The combined organic layers were washed with brine (20 mL), dried over sodium sulfate, filtered, and concentrated under reduced pressure to yield the crude product. This method was used for all hydroxymethylindole isomers.

## Synthesis and characterization

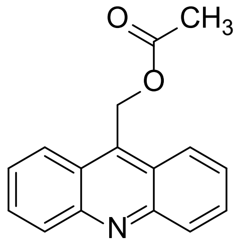

**9-Acetoxymethylacridine (1).** Equal masses of 9-bromomethylacridine (5.00 g, 18.3 mmol) and potassium acetate (5.00 g, 50.9 mmol) were refluxed in ethanol (50 mL) for 2 h. The organic layers were combined and extracted with ether (3x80 mL), washed with water (200 mL), and evaporated under reduced pressure. The resulting crude product was then recrystallized from ethanol and obtained as a glossy, pale yellow solid following vacuum filtration (3.50 g, 70%). This synthesis follows the method of Campbell *et al.*, and $^{1}H$ NMR and MS data are consistent with those reported in that work.[43]

$^{1}H$ NMR (400 MHz, DMSO-$d_6$) δ 8.44 (d, $J$ = 8.8 Hz, 2H), 8.20 (d, $J$ = 8.7 Hz, 2H), 7.89 (t, $J$ = 7.7 Hz, 2H), 7.71 (t, $J$ = 7.7 Hz, 2H), 6.14 (s, 2H), 2.03 (s, 3H). LCMS (ESI) m/z for $C_{16}H_{13}NO_2$ $[M+H]^+$: calculated: 252.102; found: 251.950.

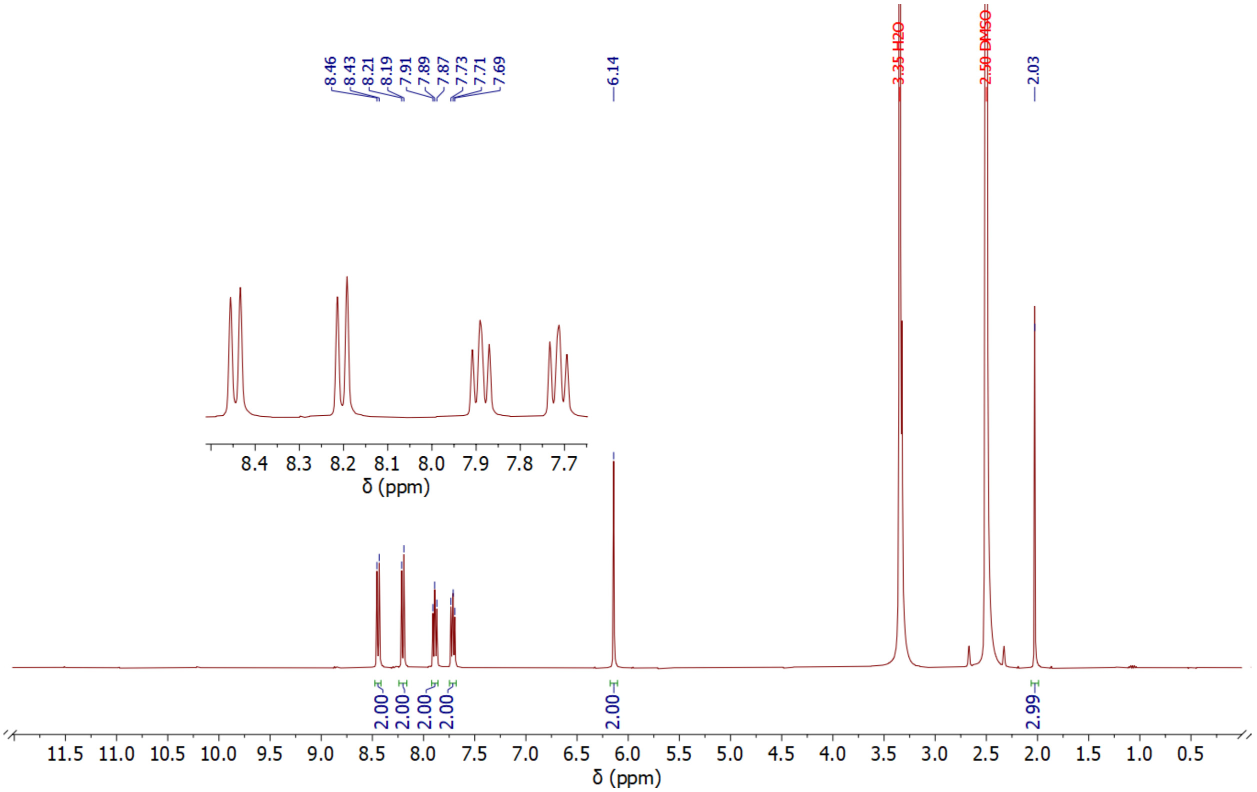


**Figure S1.** $^{1}H$ NMR spectrum of 9-acetoxymethylacridine in DMSO-$d_6$.

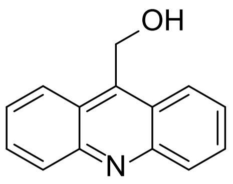

**9-Hydroxymethylacridine (2).** A portion of **1** (3.50 g, 15.6 mmol) was dissolved in ethanol (200 mL), after which 40 mL of 1 M aqueous sodium hydroxide was added. The mixture was stirred at room temperature for 20 hours. Followed by evaporation of ether, the product was recrystallized with ethanol as canary yellow granules following vacuum filtration (1.42 g, 44%). This synthesis follows the method of Campbell *et al.*, and $^1$H NMR and MS data are consistent with those reported in that work.[43]

$^1$H NMR (400 MHz, DMSO-$d_6$) δ 8.52 (d, $J$ = 8.8 Hz, 2H), 8.16 (d, $J$ = 8.7 Hz, 2H), 7.89 – 7.81 (m, 2H), 7.70 – 7.62 (m, 2H), 5.63 (t, $J$ = 5.4 Hz, 1H), 5.48 (d, $J$ = 4.9 Hz, 2H). LCMS (ESI) m/z for $C_{14}H_{11}NO$ $[M+H]^+$: calculated: 210.09; found 210.00.

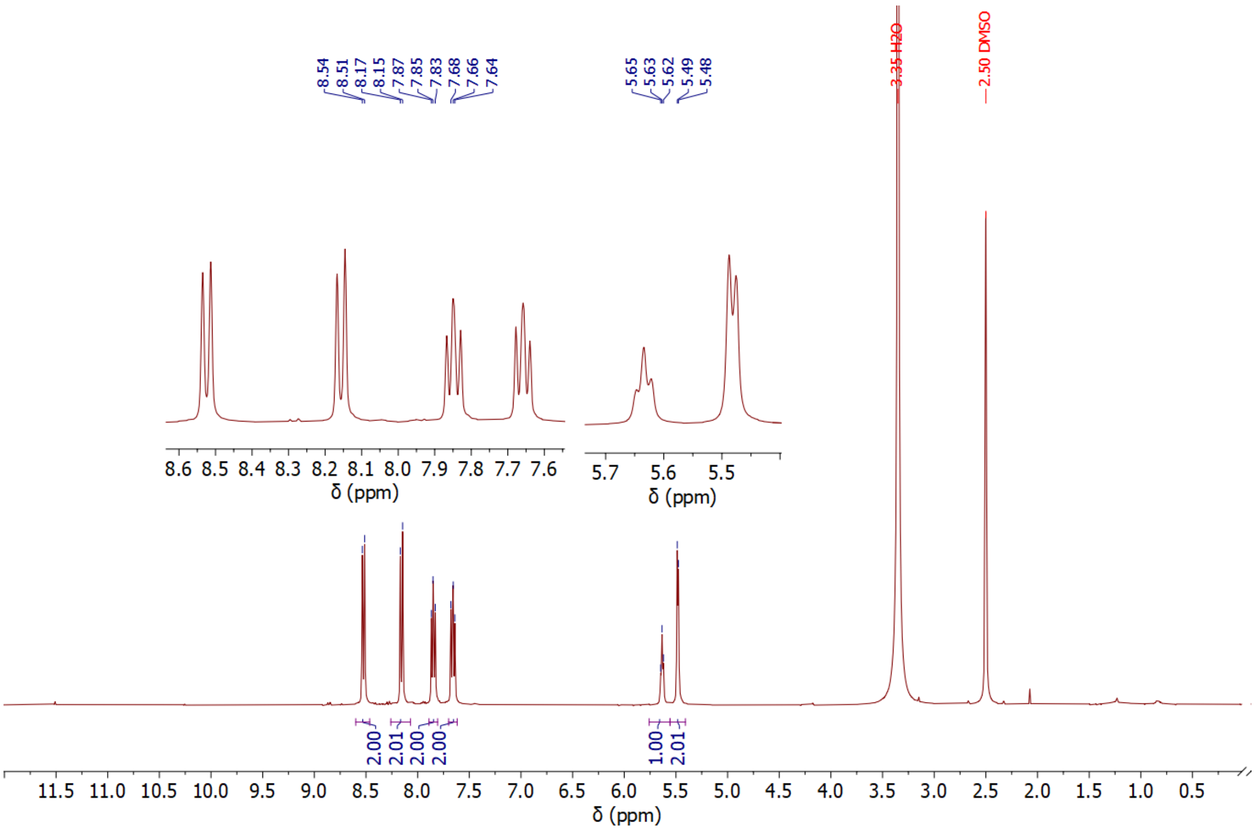


**Figure S2.** $^1$H NMR spectrum of 9-AcMeOH in DMSO-$d_6$.

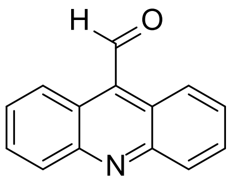


**9-Acridinecarboxaldehyde (3).** A portion of **2** (100 mg, 0.478 mmol) was oxidized for 3 h according to Method A. Flash chromatography on silica gel with 1:1 DCM/ethyl acetate gave the product as a pale yellow powder (74.6 mg, 75.4%).

$^1$H NMR (400 MHz, $CDCl_3$) δ 11.53 (s, 1H), 8.74 (d, $J$ = 8.8 Hz, 2H), 8.31 (d, $J$ = 8.8 Hz, 2H), 7.88 – 7.79 (m, 2H), 7.71 (ddd, $J$ = 8.9, 6.6, 1.3 Hz, 2H). LCMS (ESI) m/z for $C_{14}H_9NO$ $[M+H]^+$: calculated: 208.07; found 208.10.

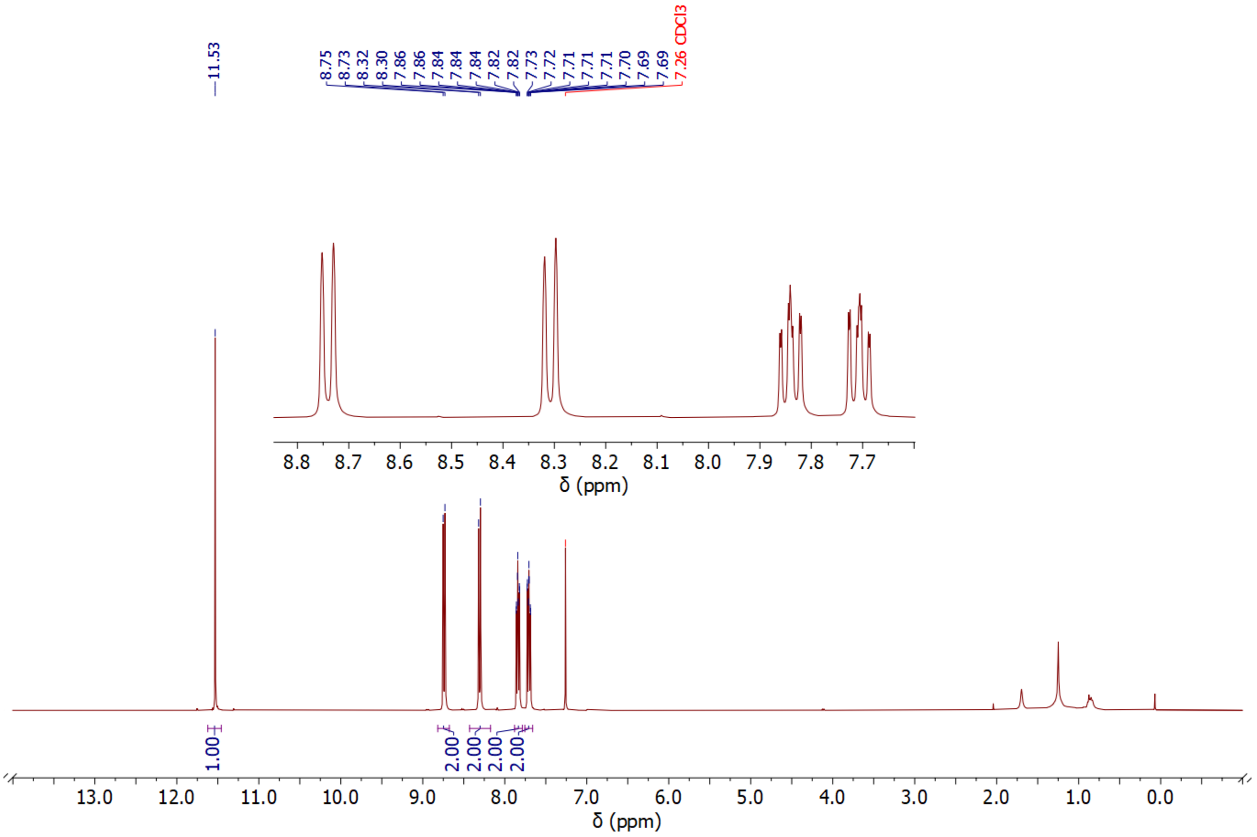


**Figure S3.** $^1$H NMR spectrum of 9-AcCHO in $CDCl_3$.

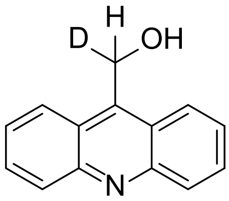


**9-Hydroxymethylacridine-*d* (4).** A portion of **3** (50.0 mg, 0.241 mmol) was dissolved in methanol (5 mL) and cooled on an ice bath. Sodium borodeuteride (93.6 mg, 2.2 mmol) was added gradually in portions, after which the solution was stirred overnight at room temperature. The reaction was quenched with water (20 mL) and extracted with DCM (3x10 mL). The combined organic layers were washed with brine (20 mL), dried over sodium sulfate, and evaporated under reduced pressure. The product was then isolated by flash chromatography on silica gel with 1:1 EtOAc/DCM (23.0 mg, 46%).

$^1$H NMR (400 MHz, DMSO-$d_6$) δ 8.52 (d, $J$ = 8.8 Hz, 2H), 8.16 (d, $J$ = 8.7 Hz, 2H), 7.85 (dd, $J$ = 8.6, 6.7 Hz, 2H), 7.66 (dd, $J$ = 8.6, 6.7 Hz, 2H), 5.61 (d, $J$ = 5.5 Hz, 1H), 5.46 (d, $J$ = 5.5 Hz, 1H).

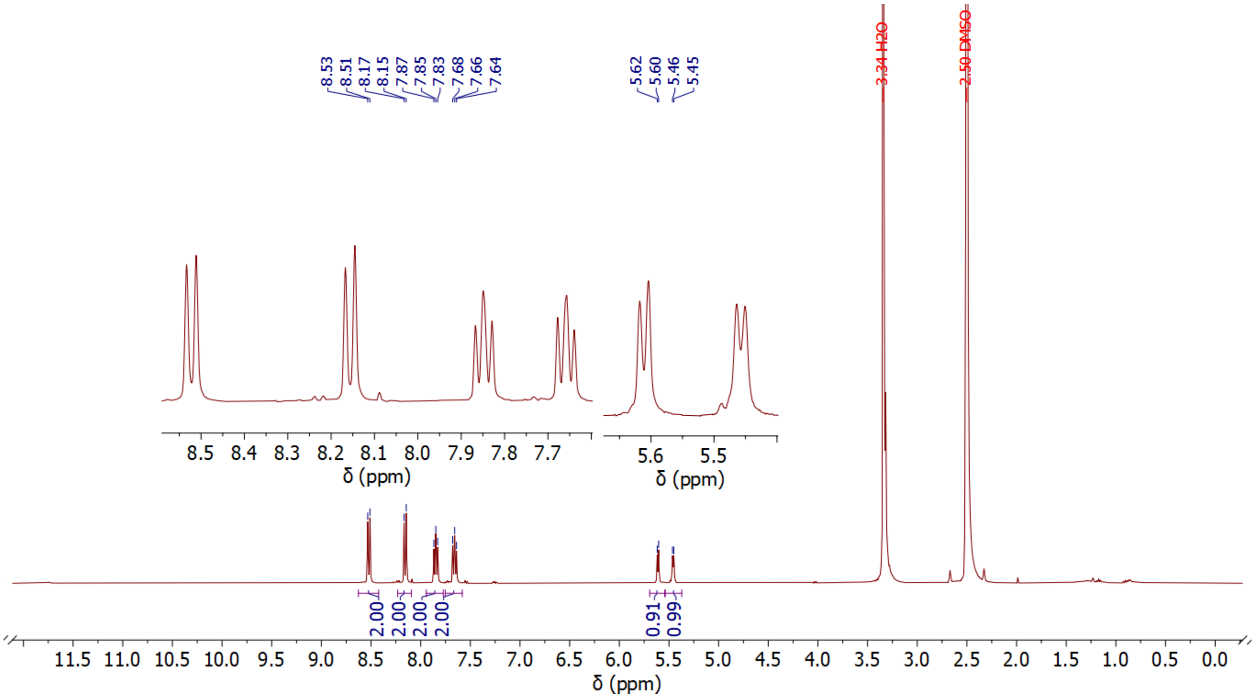


**Figure S4.** $^1$H NMR spectrum of 9-AcMeOH-$d$ in DMSO-$d_6$.

**1-, 2-, and 3-Hydroxymethylacridine (5 – 7).** The 1-, 2-, and 3- isomers of AcMeOH were prepared according to the method of Li *et al*. using the appropriate phenylboronic acid precursor.[44] 2-Hydroxymethylacridine was prepared using 4-hydroxymethylphenylboronic acid, while 1- and 3-hydroxymethyl acridine were prepared together as a mixture using 3-hydroxymethylphenylboronic acid and then separated by flash chromatography.

In a pressure tube, 2- or 3-hydroxymethylphenylboronic acid (1.90 g, 12.5 mmol), benzisoxazole (0.50 g, 0.41 mmol), and copper(II) nitrate hydrate (0.060 g), and 1.0 M HCl (0.40 mL), were combined in 1:1 DMF/trifluoroethanol (21 mL). The tube was sealed, and the mixture was stirred at 100°C overnight. After the reaction mixture was cooled to room temperature, the pH was adjusted to between 7 and 8 by dropwise addition of 1 M NaOH. The mixture was then extracted with DCM (3x30 mL). The combined organic layers were dried over sodium sulfate, and the solvent was removed under reduced pressure to give an oily paste that was dried under vacuum on an oil bath at 50°C overnight. Flash chromatography on silica gel with 3:1 EtOAc/DCM then furnished the pure product(s). Characterization of the individual compounds **5** – **7** follows on the next pages. We note that unlike all other AcMeOH isomers, compound **5** is unstable in solution even under ambient storage conditions on the timescale of days; while NMR spectra were acquired immediately following purification, peaks from decomposition products are evident especially in the $^{13}C$ NMR spectrum.

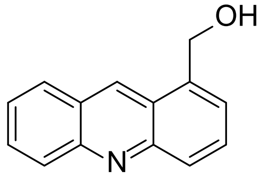

**1-Hydroxymethylacridine (5).** $^1$H NMR (400 MHz, $CDCl_3$) δ 9.11 (s, 1H), 8.22 (d, *J* = 8.8 Hz, 1H), 8.17 (d, *J* = 8.8 Hz, 1H), 8.03 (d, *J* = 8.4 Hz, 1H), 7.83 – 7.77 (m, 1H), 7.70 (dd, *J* = 8.8, 6.7 Hz, 1H), 7.56 (ddd, *J* = 8.0, 6.6, 1.2 Hz, 1H), 7.52 (dd, *J* = 6.7, 1.1 Hz, 1H), 5.27 (s, 2H). $^{13}$C NMR (101 MHz, $CDCl_3$) δ 149.39, 148.80, 137.08, 133.05, 130.68, 129.97, 129.81, 129.22, 128.73, 126.49, 126.03, 124.98, 63.69, 29.85. LCMS (ESI) m/z for $C_{14}H_{11}NO$ $[M+H]^+$: calculated: 210.09; found 210.00.

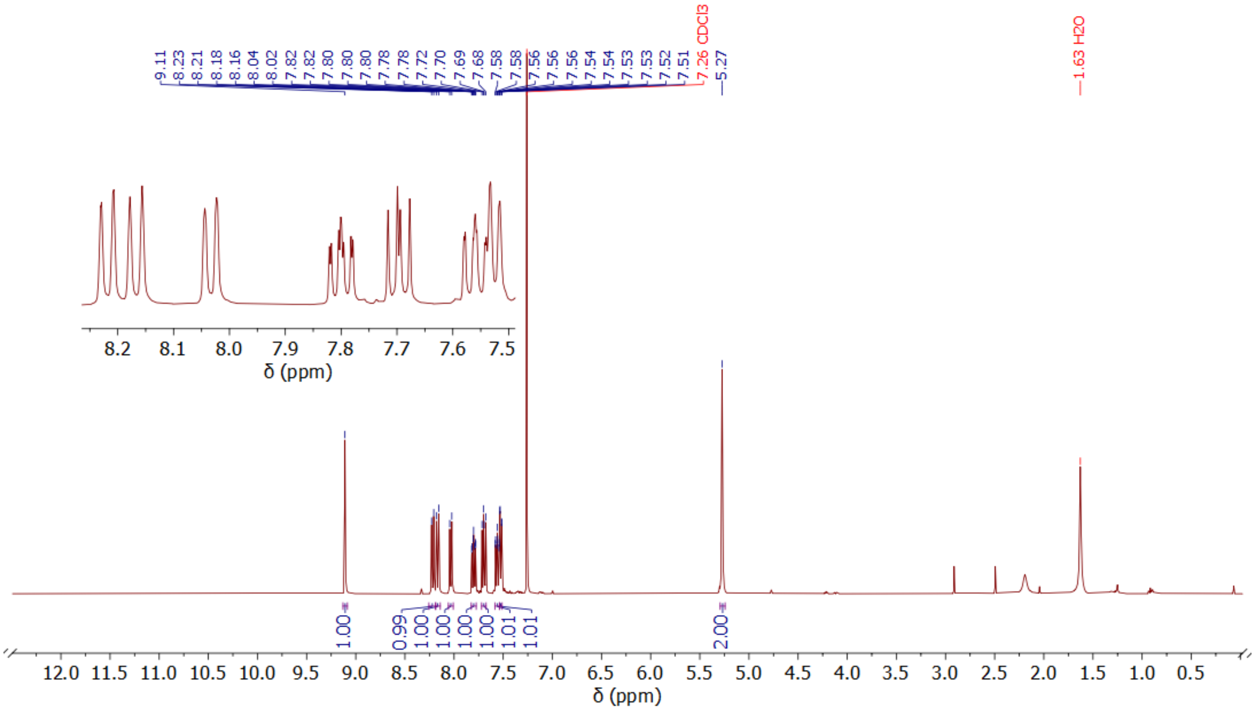


**Figure S5.** $^1$H NMR spectrum of 1-AcMeOH in $CDCl_3$.

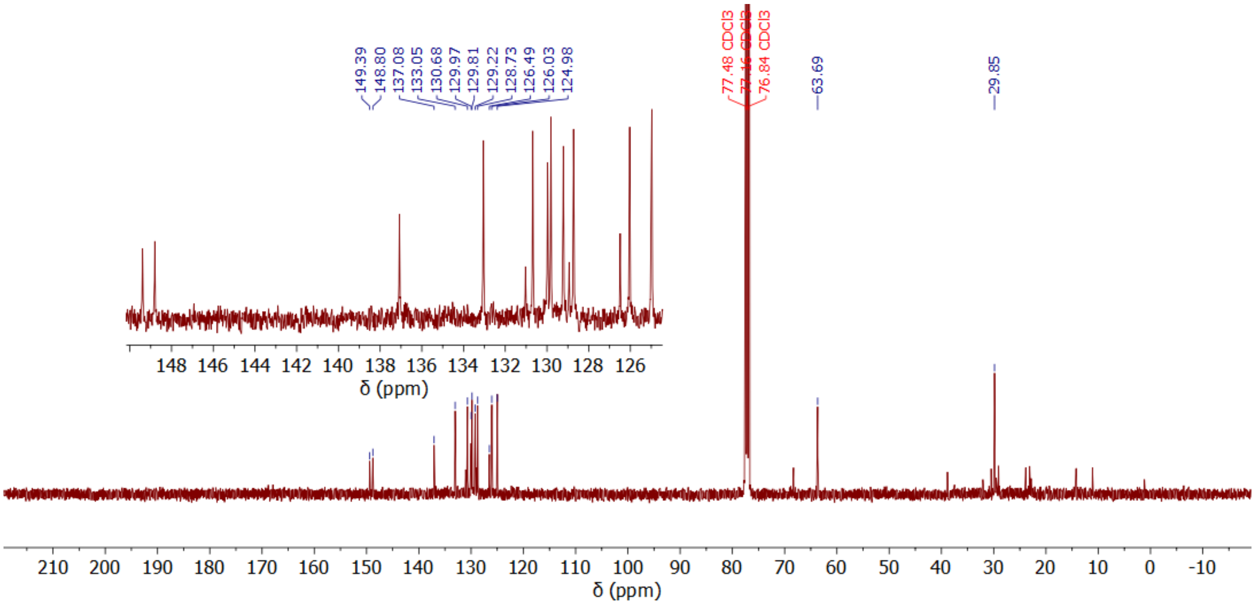


**Figure S6.** $^{13}$C NMR spectrum of 1-AcMeOH in $CDCl_3$.

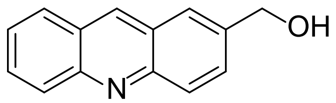


**2-Hydroxymethylacridine (6).** $^{1}H$ NMR (400 MHz, $CDCl_3$) δ 8.70 (s, 1H), 8.22 (d, $J$ = 8.8 Hz, 1H), 8.18 (d, $J$ = 9.0 Hz, 1H), 7.99 (d, $J$ = 8.5 Hz, 1H), 7.91 (s, 1H), 7.78 (ddd, $J$ = 8.9, 6.6, 1.4 Hz, 1H), 7.73 (dd, $J$ = 9.0, 1.9 Hz, 1H), 7.54 (ddd, $J$ = 7.9, 6.6, 1.2 Hz, 1H), 4.92 (s, 2H). $^{13}C$ NMR (101 MHz, $CDCl_3$) δ 148.99, 148.76, 138.51, 136.11, 130.46, 130.08, 129.64, 129.36, 128.30, 126.86, 126.45, 125.93, 124.93, 77.16, 65.00. LCMS (ESI) m/z for $C_{14}H_{11}NO$ $[M+H]^+$: calculated: 210.09; found 210.00.

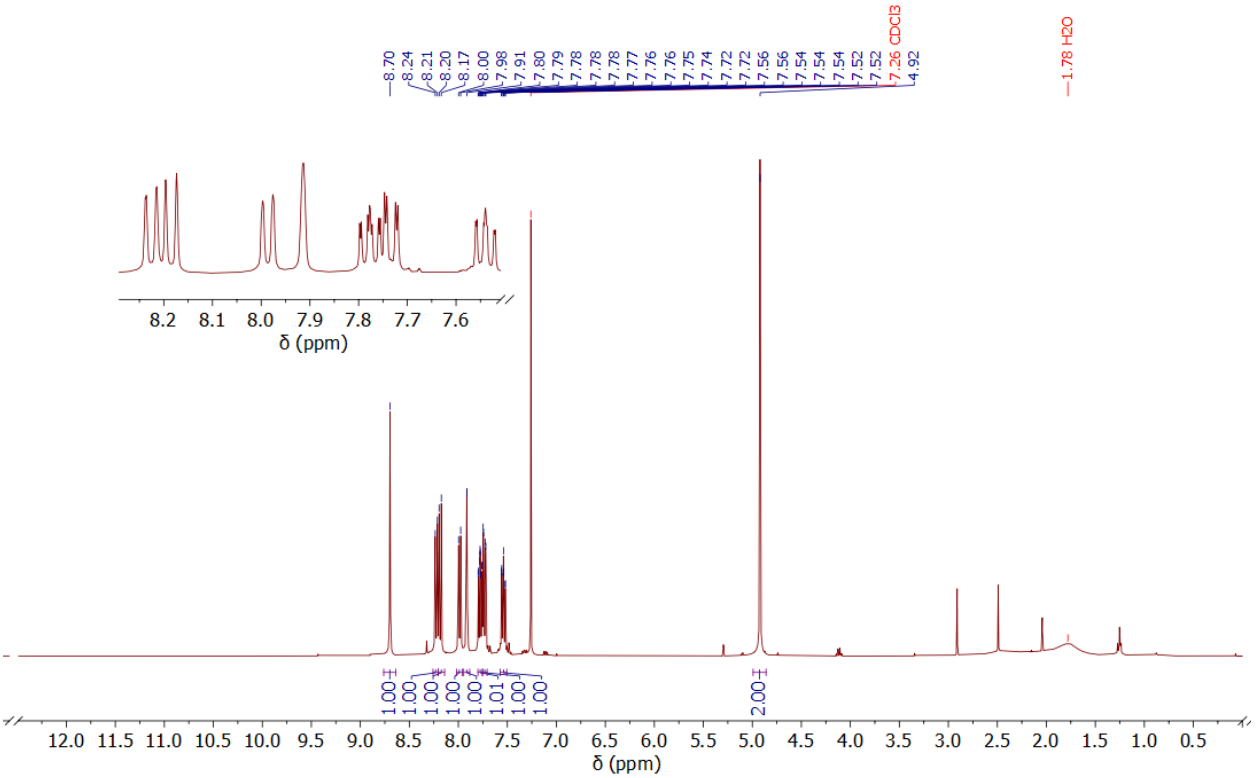


**Figure S7.** $^{1}H$ NMR spectrum of 2-AcMeOH in $CDCl_3$.

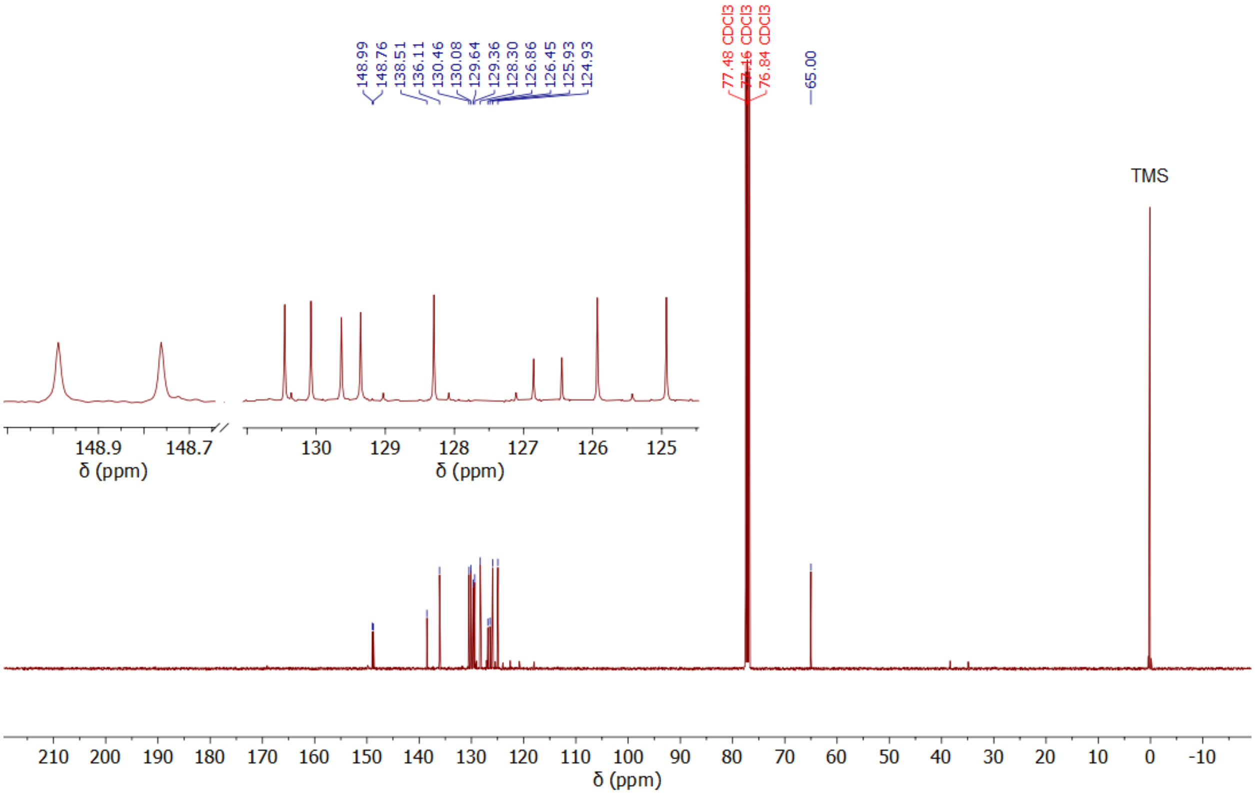


**Figure S8.** $^{13}$C NMR spectrum of 2-AcMeOH in $CDCl_3$.

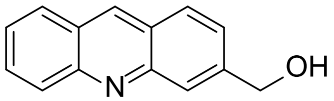


**3-Hydroxymethylacridine (7).** $^{1}$H NMR (400 MHz, $CDCl_3$) δ 8.72 (s, 1H), 8.30 (s, 1H), 8.25 (d, $J$ = 8.8 Hz, 1H), 7.98 (d, $J$ = 8.5 Hz, 1H), 7.93 (d, $J$ = 8.7 Hz, 1H), 7.79 (ddd, $J$ = 8.8, 6.7, 1.4 Hz, 1H), 7.53 (ddd, $J$ = 8.1, 6.6, 1.1 Hz, 1H), 7.48 (dd, $J$ = 8.7, 1.6 Hz, 1H), 4.96 (d, $J$ = 1.2 Hz, 2H). $^{13}$C NMR (101 MHz, $CDCl_3$) δ 144.28, 136.20, 130.63, 129.15, 128.49, 128.37, 126.05, 125.77, 125.71, 125.27, 77.16, 65.00. LCMS (ESI) m/z for $C_{14}H_{11}NO$ $[M+H]^{+}$: calculated: 210.09; found 210.00.

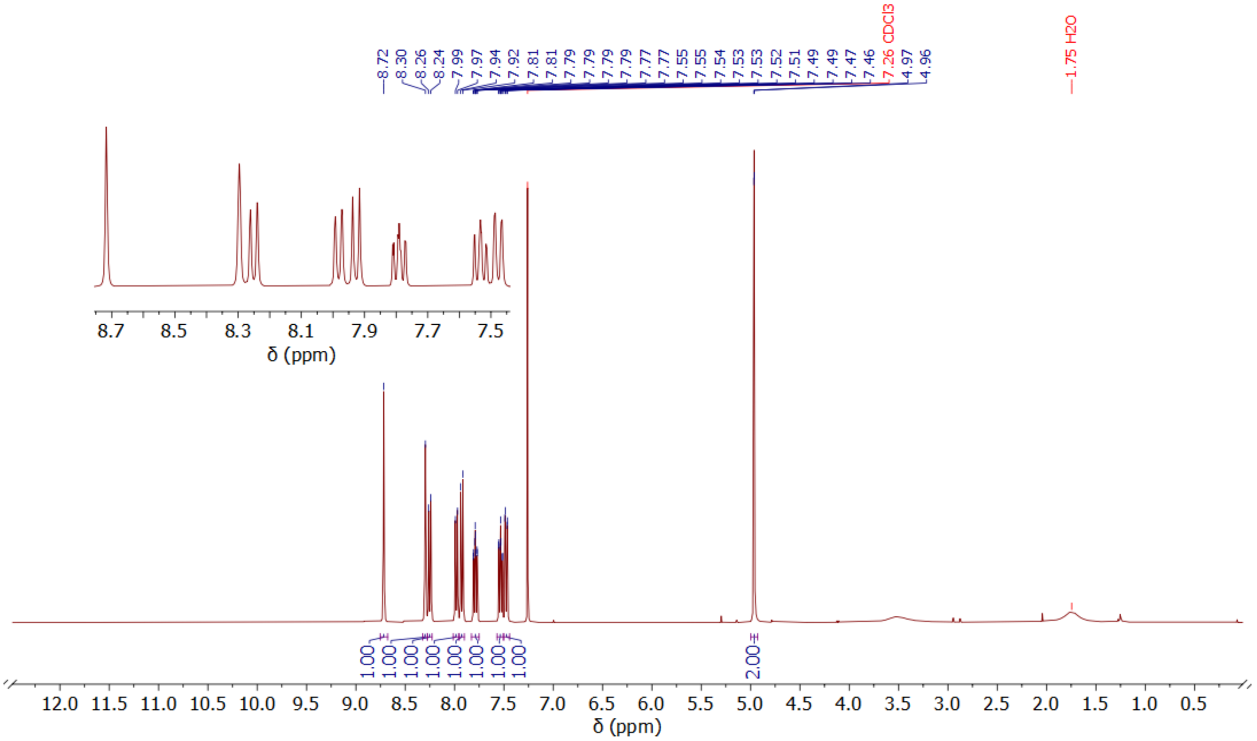


**Figure S9.** $^{1}$H NMR spectrum of 3-AcMeOH in $CDCl_3$.

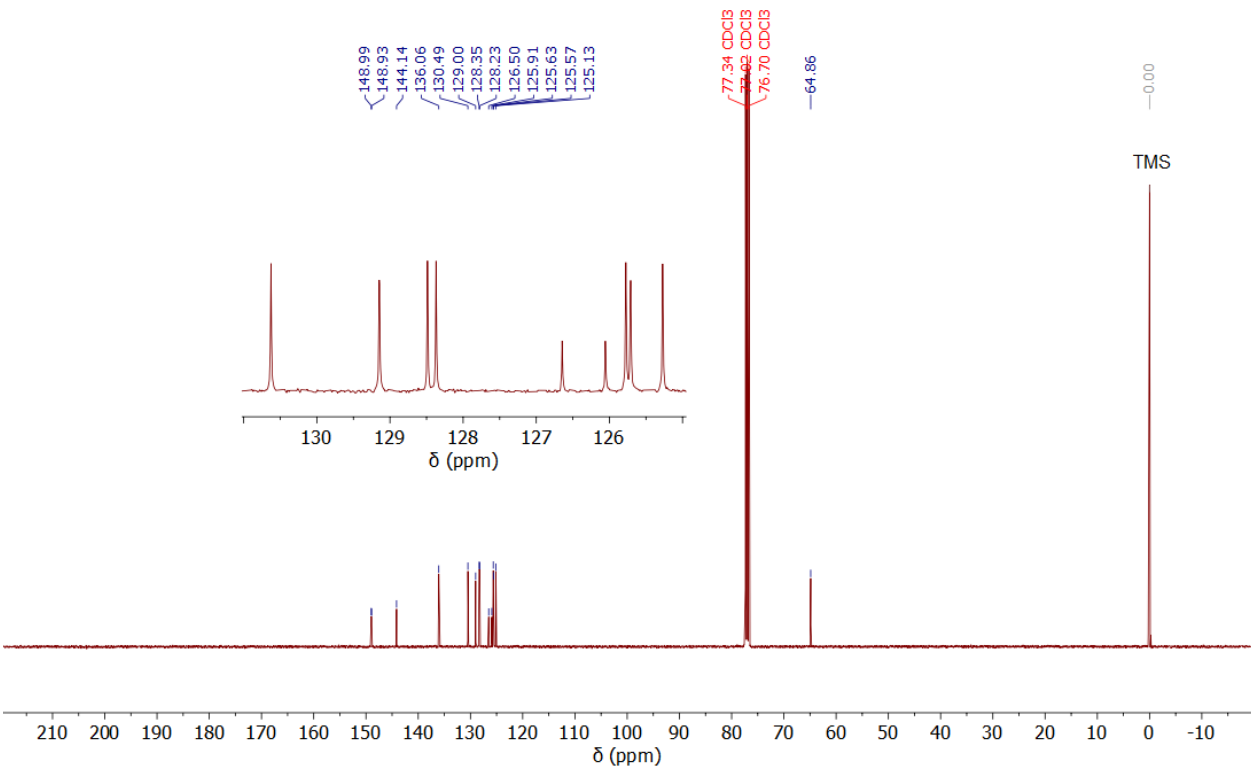


**Figure S10.** $^{13}$C NMR spectrum of 3-AcMeOH in $CDCl_3$.

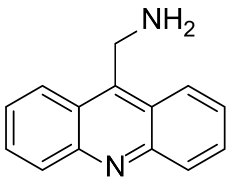


**9-Aminomethylacridine (8).** 9-Bromomethylacridine (598 mg, 2.20 mmol) was combined with hexamethylenetetramine (340 mg, 2.40 mmol) in DCM (40.0 mL) and refluxed overnight. The reaction mixture was cooled to room temperature, and the precipitate was filtered and washed with diethyl ether. The precipitate was then refluxed in ethanol (30.0 mL) with concentrated hydrochloric acid (4.40 mL) for 3 hours. The resulting precipitate was filtered and washed with diethyl ether to give the bis(hydrochloride) salt. The salt was dissolved in water (20 mL), and the pH was adjusted to between 7 and 8 by dropwise addition of a saturated solution of sodium hydroxide. The solution was then extracted with DCM (3x20 mL), and the combined organic layers were dried over sodium sulfate and concentrated under reduced pressure to yield the free base as a dark blue resinous solid (300 mg, 72%). This method was adapted from that reported by Camerel *et al.*[47]

$^{1}$H NMR (400 MHz, $CDCl_3$) δ 8.33 (d, *J* = 8.6 Hz, 2H), 8.25 (d, *J* = 8.7 Hz, 2H), 7.79 (ddd, *J* = 8.8, 6.5, 1.3 Hz, 2H), 7.61 (ddd, *J* = 8.8, 6.6, 1.3 Hz, 2H), 4.83 (s, 2H), 1.25 (s, 2H). $^{13}$C NMR (101 MHz, $CDCl_3$) δ 149.08, 145.36, 130.64, 129.96, 126.35, 124.30, 123.78, 37.78. LCMS (ESI) m/z for $C_{14}H_{12}N_2$ $[M+H]^+$: calculated: 209.10; found 209.00.

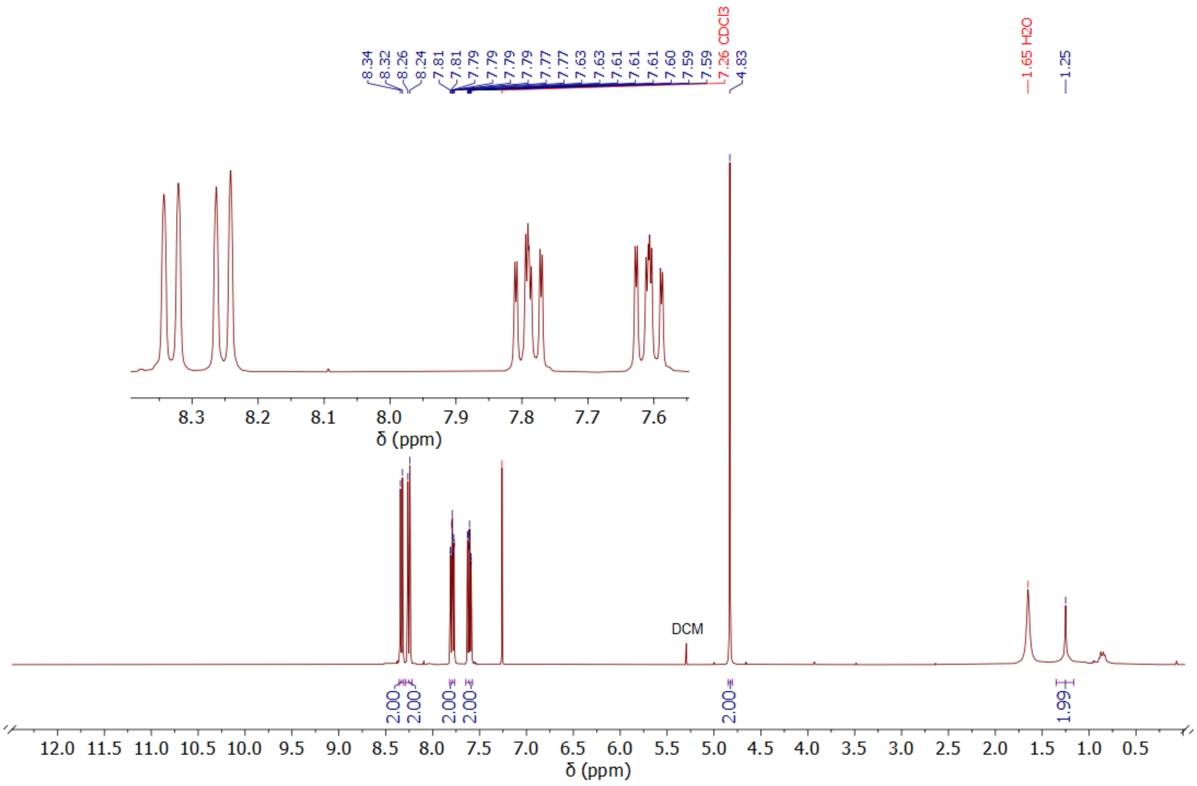


**Figure S11.** $^{1}$H NMR spectrum of 9-AcMeNH$_2$ in $CDCl_3$.

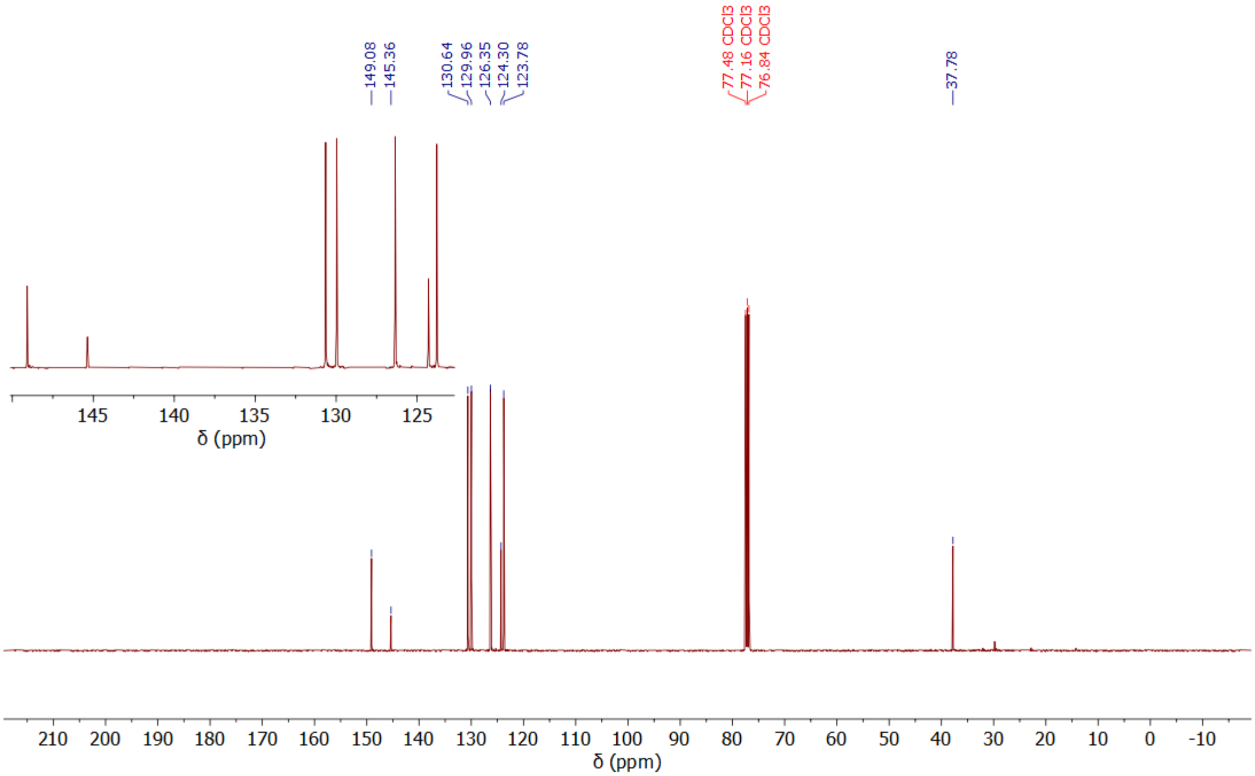


**Figure S12.** $^{13}$C NMR spectrum of 9-AcMeNH$_2$ in $CDCl_3$.

## Optimization of reaction conditions

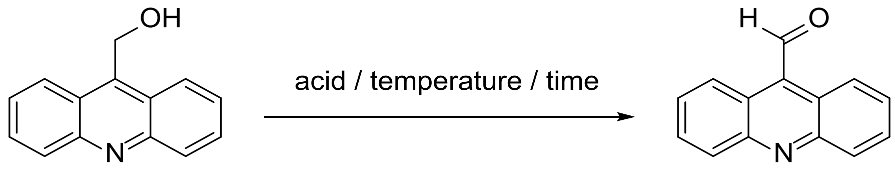


| Entry | Acid | Acid conc. (v/v) | Conversion (%) |
|---|---|---|---|
| 1 | HCl | 5% | 76 |
| 2 | HOAc | 2% | 64 |
| 3 | HOAc | 5% | 100 |
| 4 | white vinegar | ~5% | 75 |

**Table S1.** Screening acid identity and concentration for the oxidation of **2** to **3**. All reactions were performed at room temperature with 5 mM solutions of **2**, and percent conversion after 72 h was determined by NMR as described in the general procedures section above.

| Entry | Acid | Temp. (°C) | Time (h) | Conversion (%) |
|---|---|---|---|---|
| 1 | HCl | 25 ± 3 | 24 | 18 |
| 2 | HOAc | 25 ± 3 | 24 | 40 |
| 3 | HOAc | 101 | 1 | 84 |
| 4 | HOAc | 101 | 2 | 96 |
| 5 | HOAc | 101 | 4 | 100 |

**Table S2.** Screening reaction time and temperature for the oxidation of **2** to **3**. All reactions were performed with 5 mM solutions of **2** in 5% (v/v) aqueous acid, and percent conversion was determined by NMR as described in the general procedures section above.

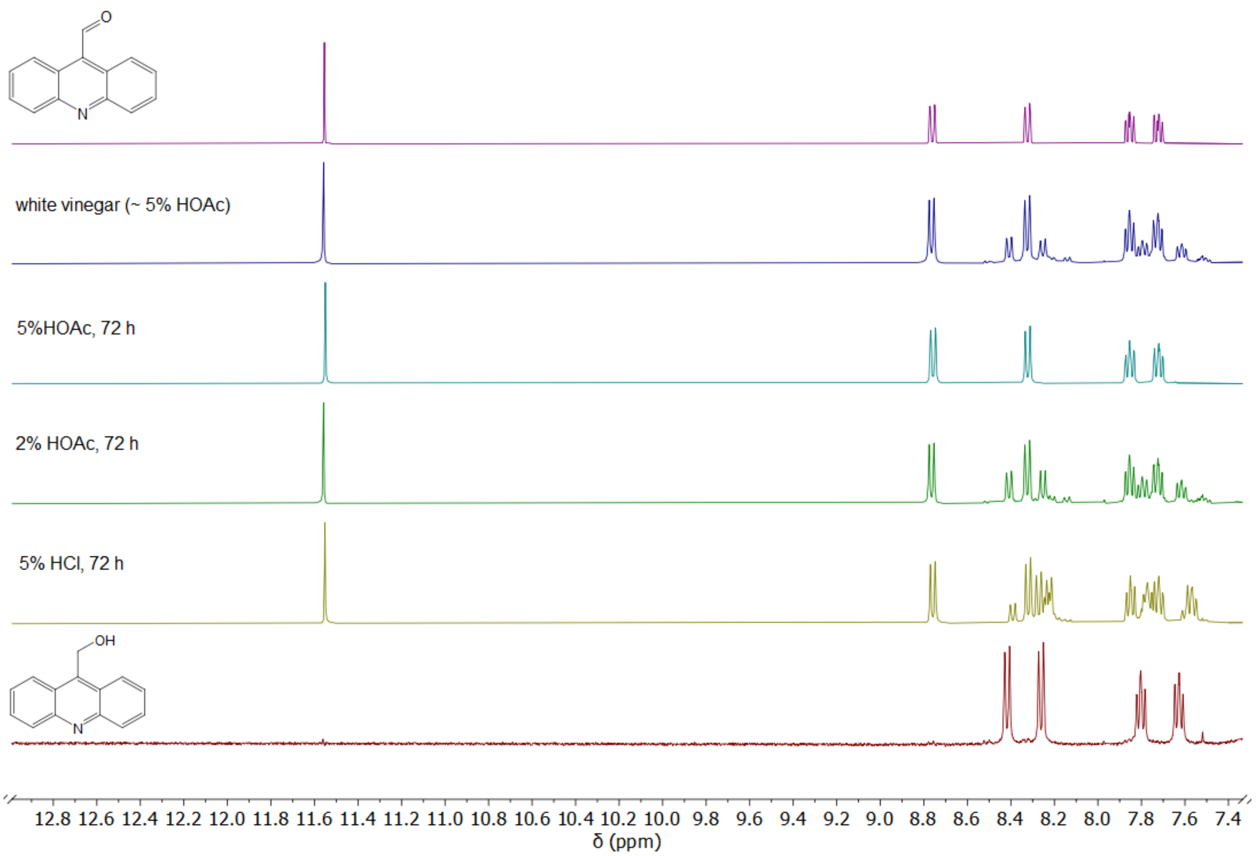


**Figure S13.** Crude $^{1}H$ NMR spectra showing the oxidation of **2** to **3** at room temperature after 72 h in in the presence of HCl or HOAc at different concentrations. For comparison, the spectra of the pure starting material and product are shown at the bottom and top, respectively.

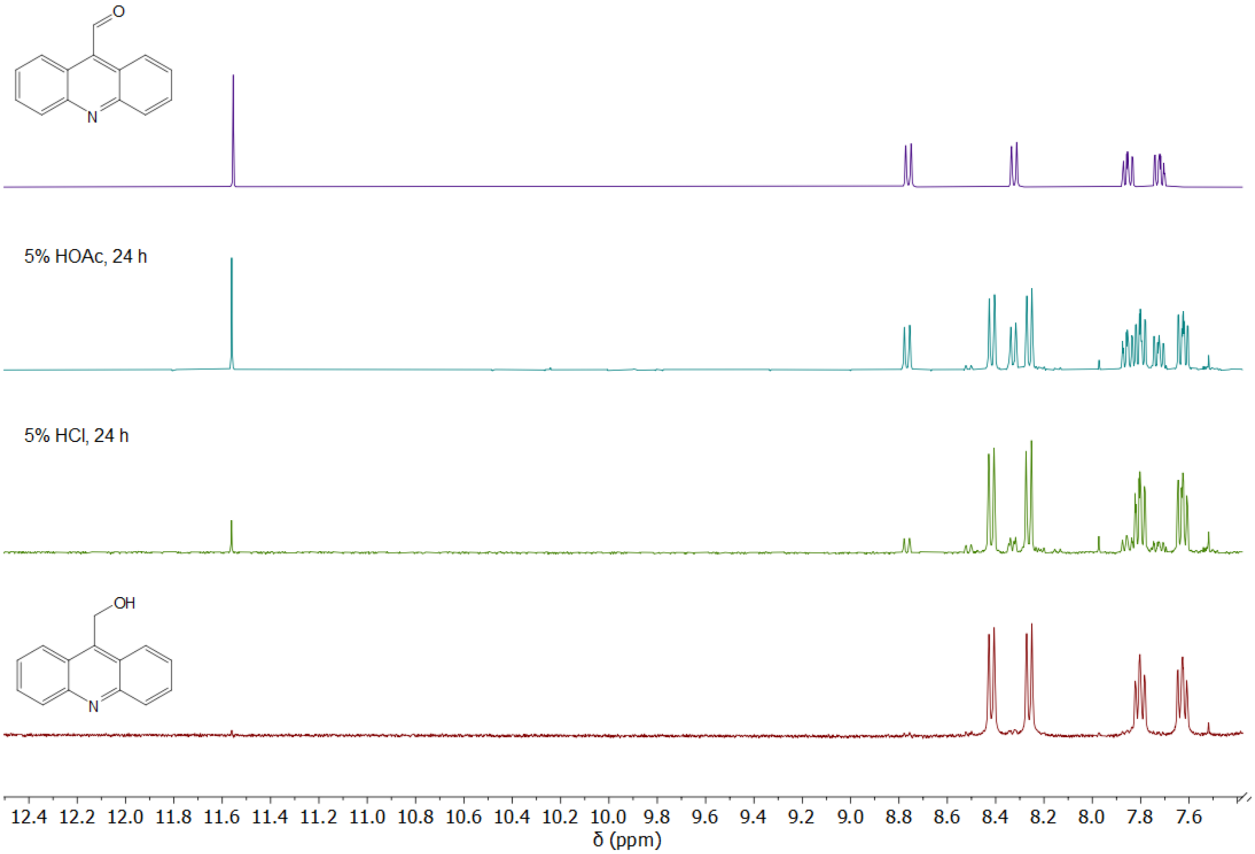


**Figure S14.** Crude $^{1}H$ NMR spectra showing the oxidation of **2** to **3** over time at room temperature in 5% (v/v) aqueous HCl and HOAc. For comparison, the spectra of the pure starting material and product are shown at the bottom and top, respectively.

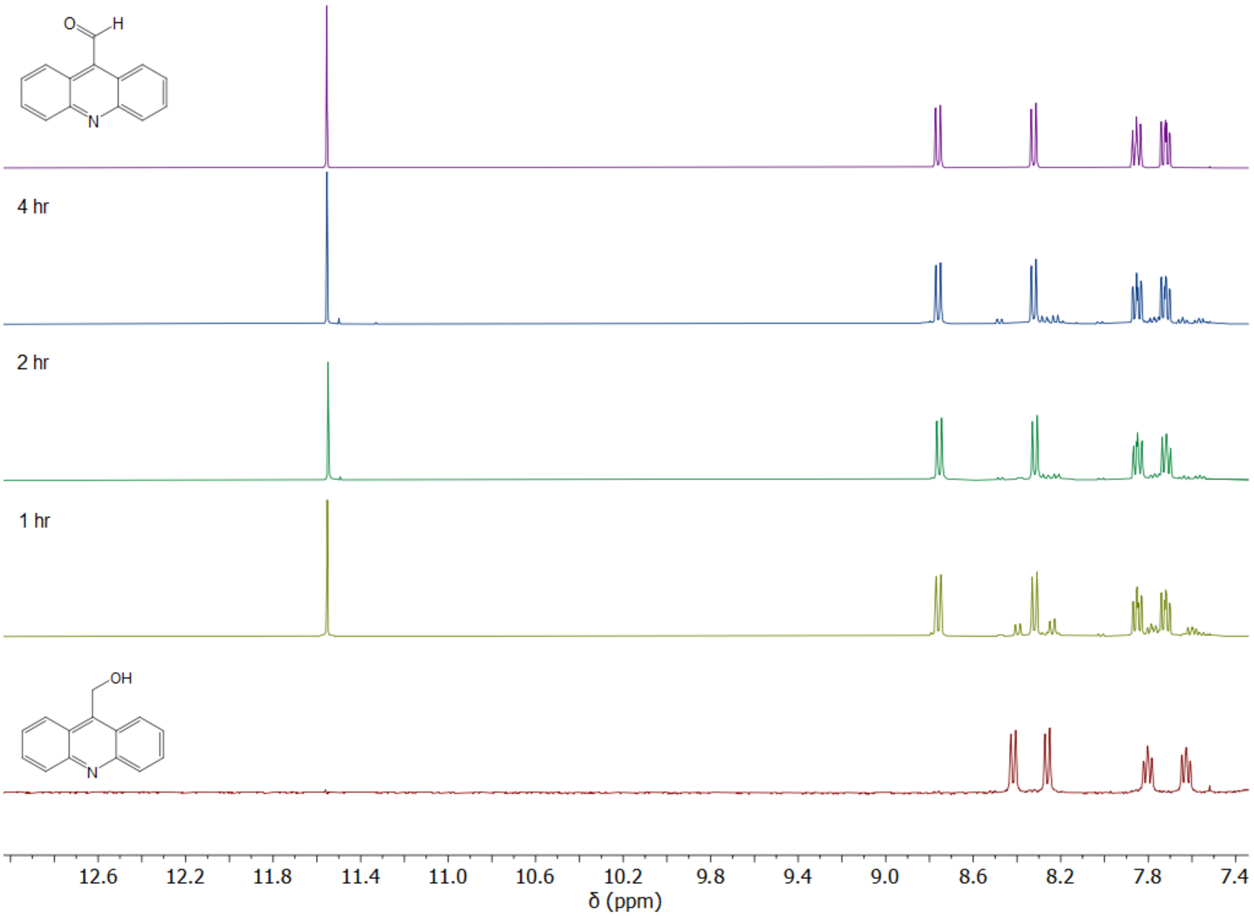


**Figure S15.** Crude $^{1}H$ NMR spectra showing the oxidation of **2** to **3** over time under the conditions of Method A. For comparison, the spectra of the pure starting material and product are shown at the bottom and top, respectively.

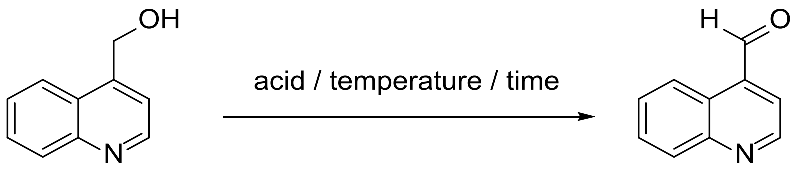


| Entry | Acid | Acid conc. (v/v) | Temp. (°C) | Time (h) | Conversion (%) |
|---|---|---|---|---|---|
| 1 | HCl | 5% | 25 ± 3 | 96 | <1 |
| 2 | HCl | 5% | 101 | 72 | 8 |
| 3 | HCl | 5% | 160 | 24 | 2 |
| 4 | HCl | 5% | 200 | 24 | 18 |
| 5 | HCl | 5% | 230 | 24 | 100 |
| 6 | HOAc | 5% | 220 | 7 | 100 |

**Table S3.** Screening acid identity and concentration and reaction time and temperature for the oxidation of 4-QuMeOH. All reactions were performed with 5 mM aqueous solutions of 4-QuMeOH, and percent conversion was determined by NMR as described in the general procedures section above.

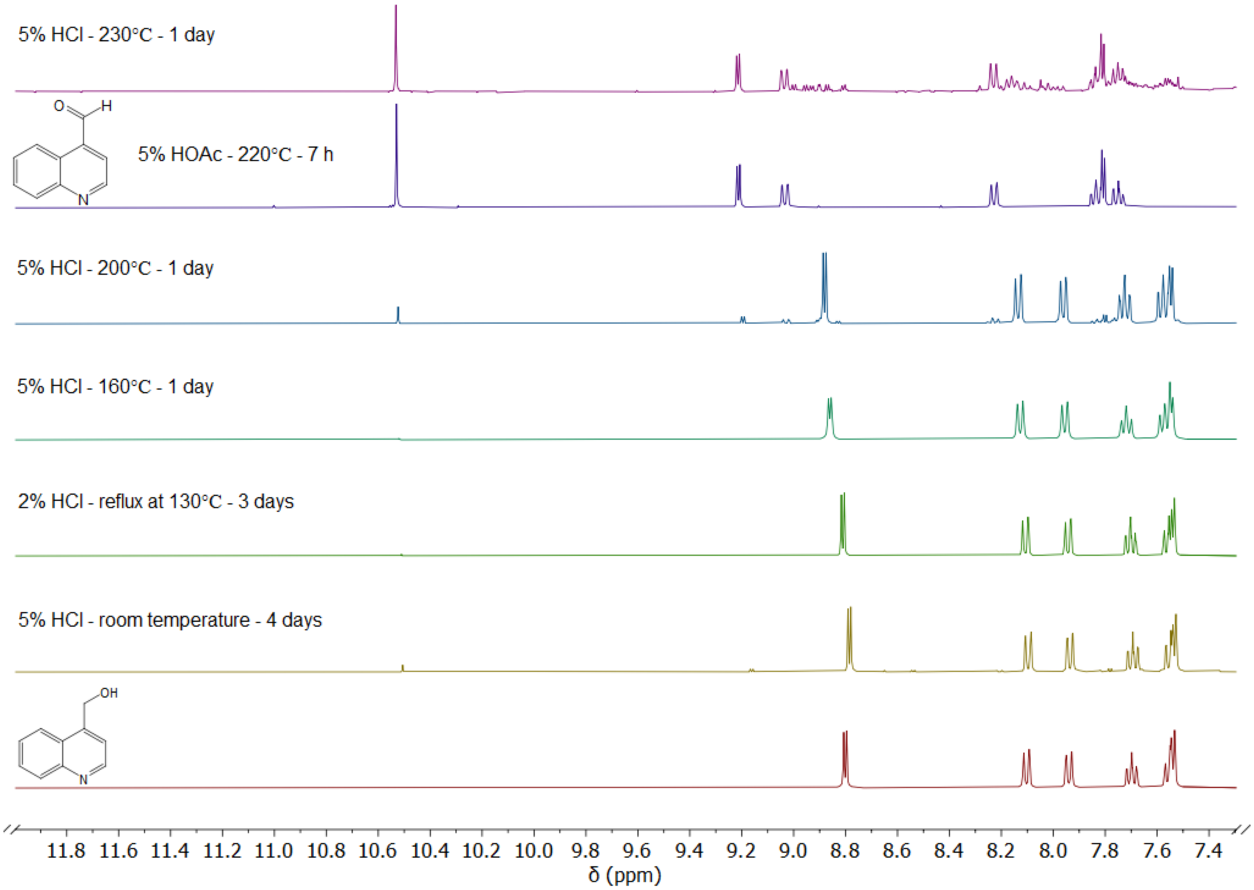


**Figure S16.** Crude $^1$H NMR spectra showing the oxidation of 4-QuMeOH to 4-QuCHO (**11**) over time under various conditions. For comparison, the spectra of the pure starting material and product are shown at the bottom and top, respectively.

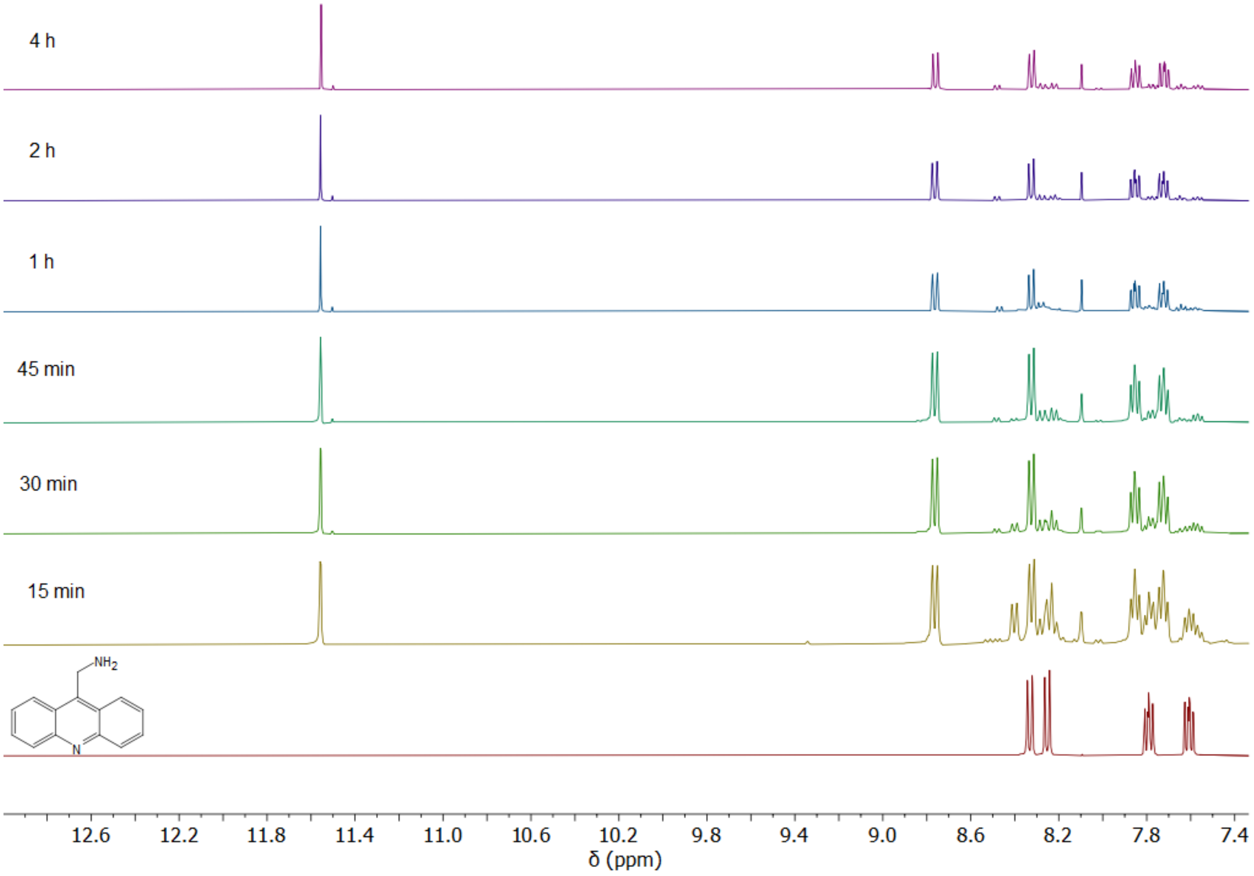


**Figure S17.** Crude $^{1}$H NMR spectra showing the oxidation of **8** to **3** over time under the conditions of Method A. For comparison, the spectra of the pure starting material and product are shown at the bottom and top, respectively.

## Additional oxidation reactions

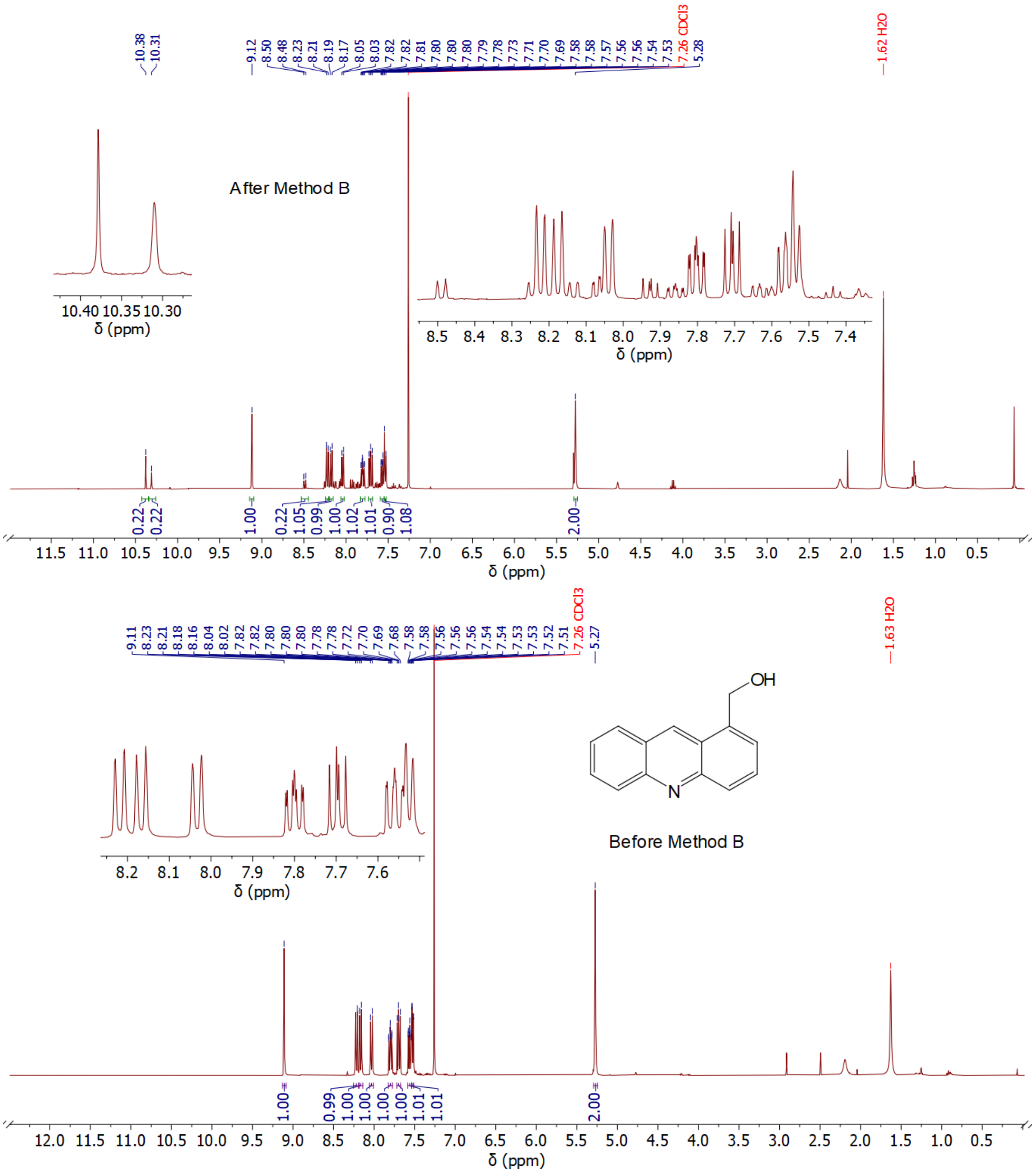


**Figure S18.** Comparison of $^{1}$H NMR spectra ($CDCl_3$) of 1-AcMeOH (**5**) before and after stirring for 9 h under the conditions of Method B, showing 15% conversion. An additional unidentified side product is also observed.

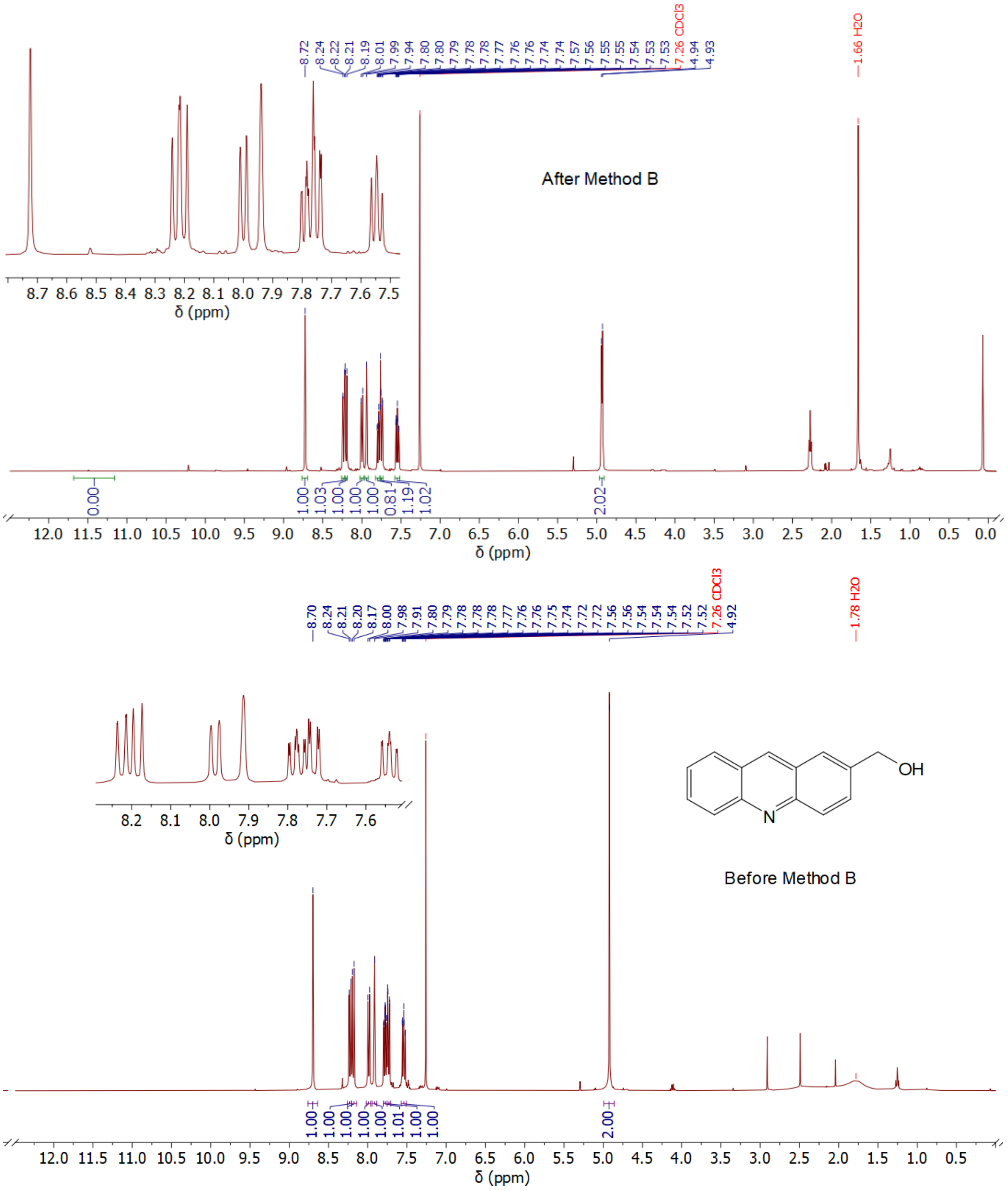


**Figure S19.** Comparison of $^{1}$H NMR spectra ($CDCl_3$) of 2-AcMeOH (**6**) before and after stirring for 7 h under the conditions of Method B, showing <1% conversion.

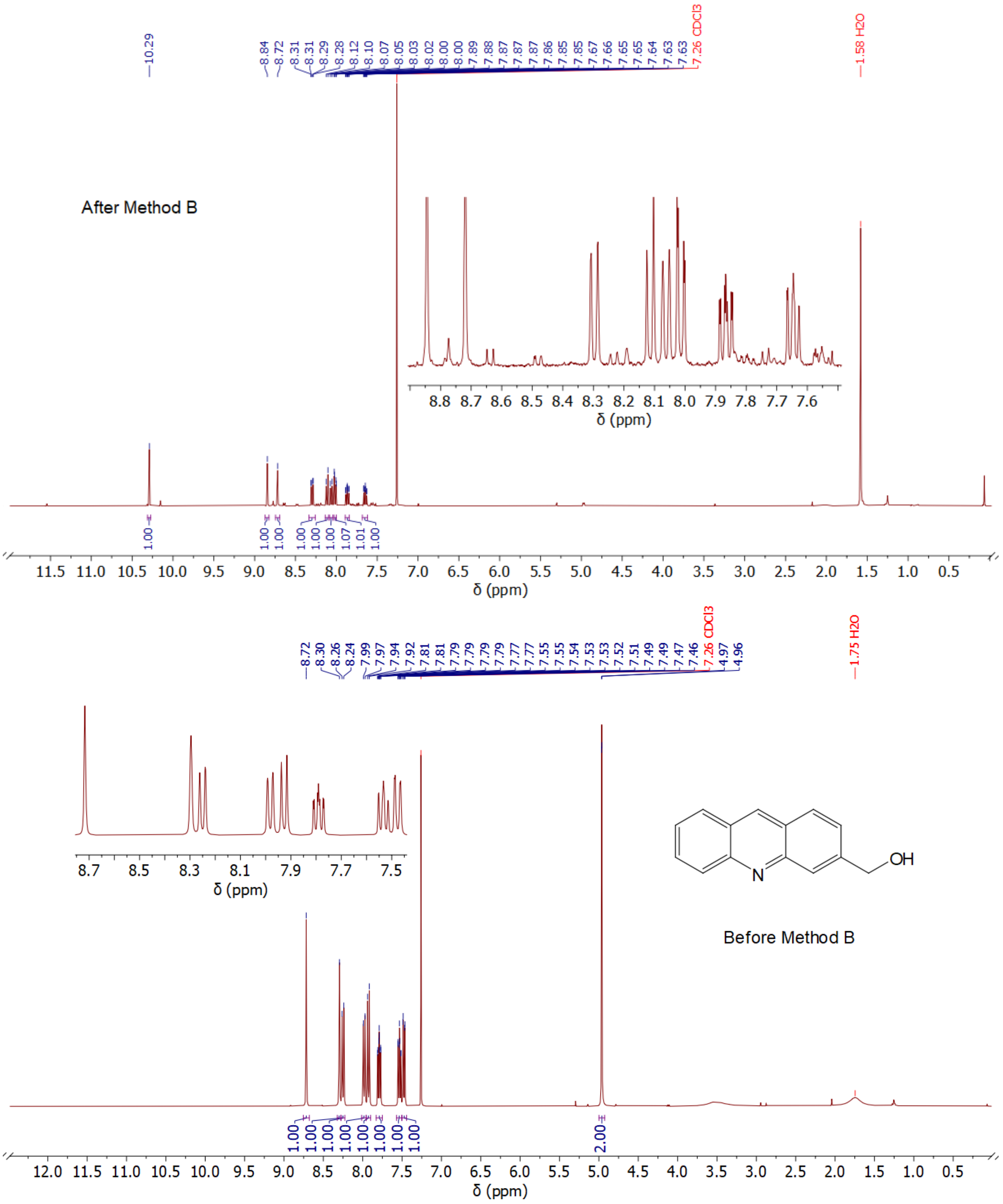


**Figure S20.** Comparison of $^{1}$H NMR spectra ($CDCl_3$) of 3-AcMeOH (**7**) before and after stirring for 4 h under the conditions of Method B, showing quantitative conversion to 3-AcCHO (**9**).

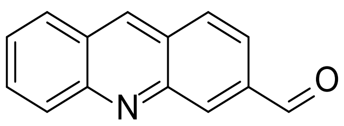

**3-Acridinecarboxaldehyde (9).** $^1$H NMR (400 MHz, $CDCl_3$) δ 10.29 (s, 1H), 8.84 (s, 1H), 8.72 (s, 1H), 8.30 (dd, *J* = 8.8, 1.0 Hz, 1H), 8.11 (d, *J* = 8.8 Hz, 1H), 8.06 (d, *J* = 8.5 Hz, 1H), 8.01 (dd, *J* = 8.7, 1.5 Hz, 1H), 7.87 (ddd, *J* = 8.8, 6.6, 1.4 Hz, 1H), 7.65 (ddd, *J* = 8.0, 6.6, 1.1 Hz, 1H). LCMS (ESI) m/z for $C_{14}H_9NO$ $[M+H]^+$: calculated: 208.07; found 208.10.

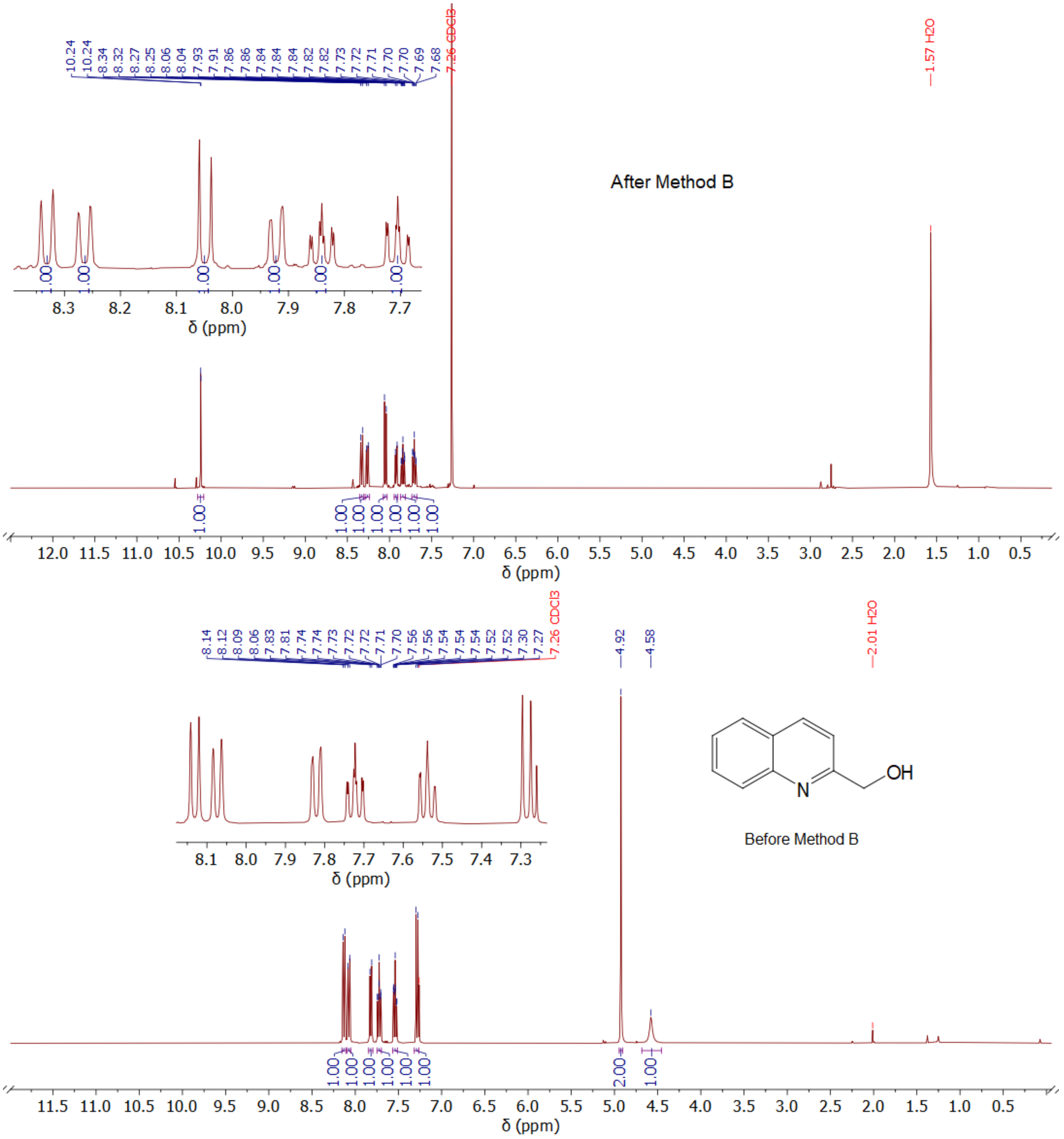


**Figure S21.** Comparison of $^{1}$H NMR spectra ($CDCl_3$) of 2-QuMeOH before and after stirring for 4 h under the conditions of Method B, showing quantitative conversion to 2-QuCHO (**10**).

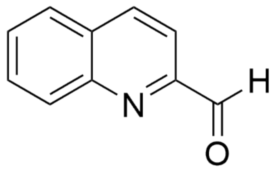

**2-Quinolinecarboxaldehyde (10).** $^{1}H$ NMR (400 MHz, $CDCl_3$) δ 10.24 (d, $J$ = 0.9 Hz, 1H), 8.33 (d, $J$ = 8.4 Hz, 1H), 8.26 (d, $J$ = 8.6 Hz, 1H), 8.05 (d, $J$ = 8.4 Hz, 1H), 7.92 (d, $J$ = 8.2 Hz, 1H), 7.84 (ddd, $J$ = 8.5, 6.9, 1.5 Hz, 1H), 7.70 (ddd, $J$ = 8.1, 6.9, 1.2 Hz, 1H). LCMS (ESI) m/z for $C_{10}H_7NO$ $[M+H]^+$: calculated: 158.052; found 158.05.

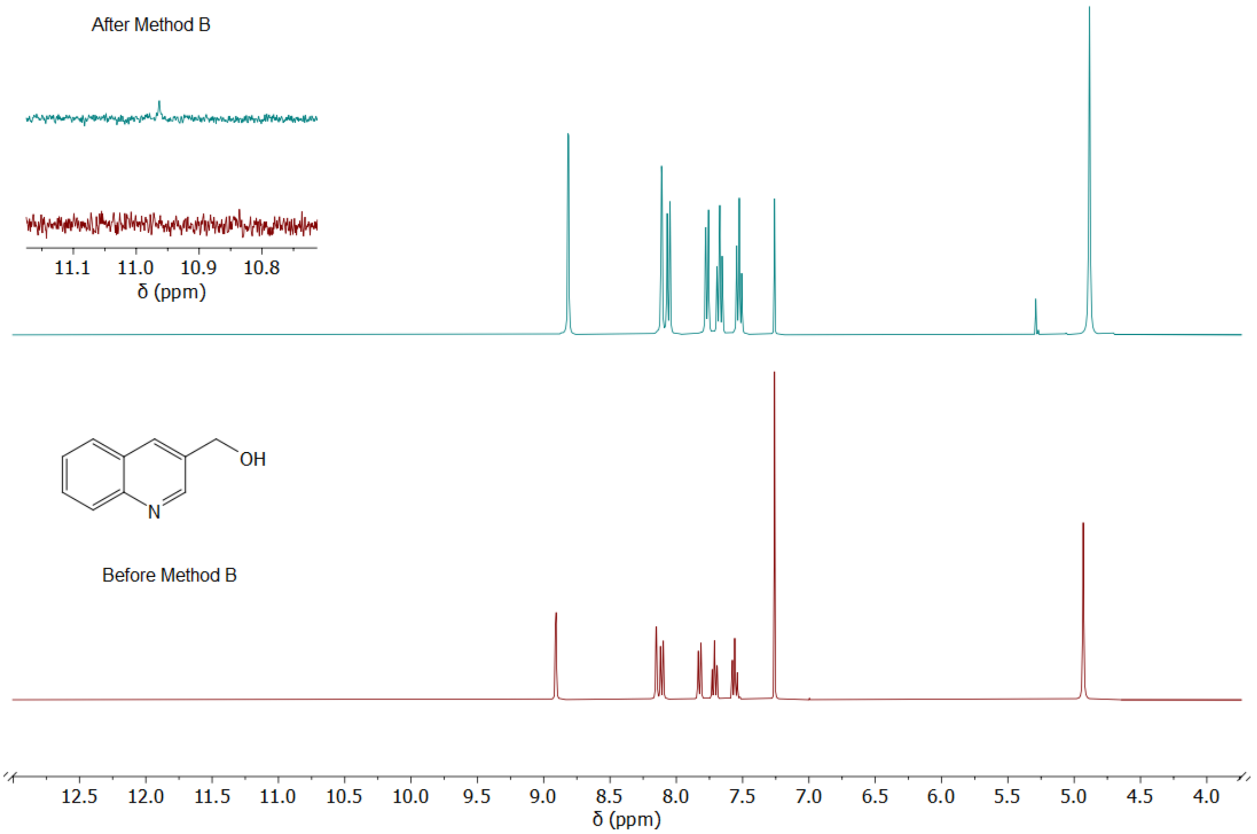


**Figure S22.** Comparison of $^1$H NMR spectra ($CDCl_3$) of 3-QuMeOH before and after stirring for 7 h under the conditions of Method B, showing <1% conversion.

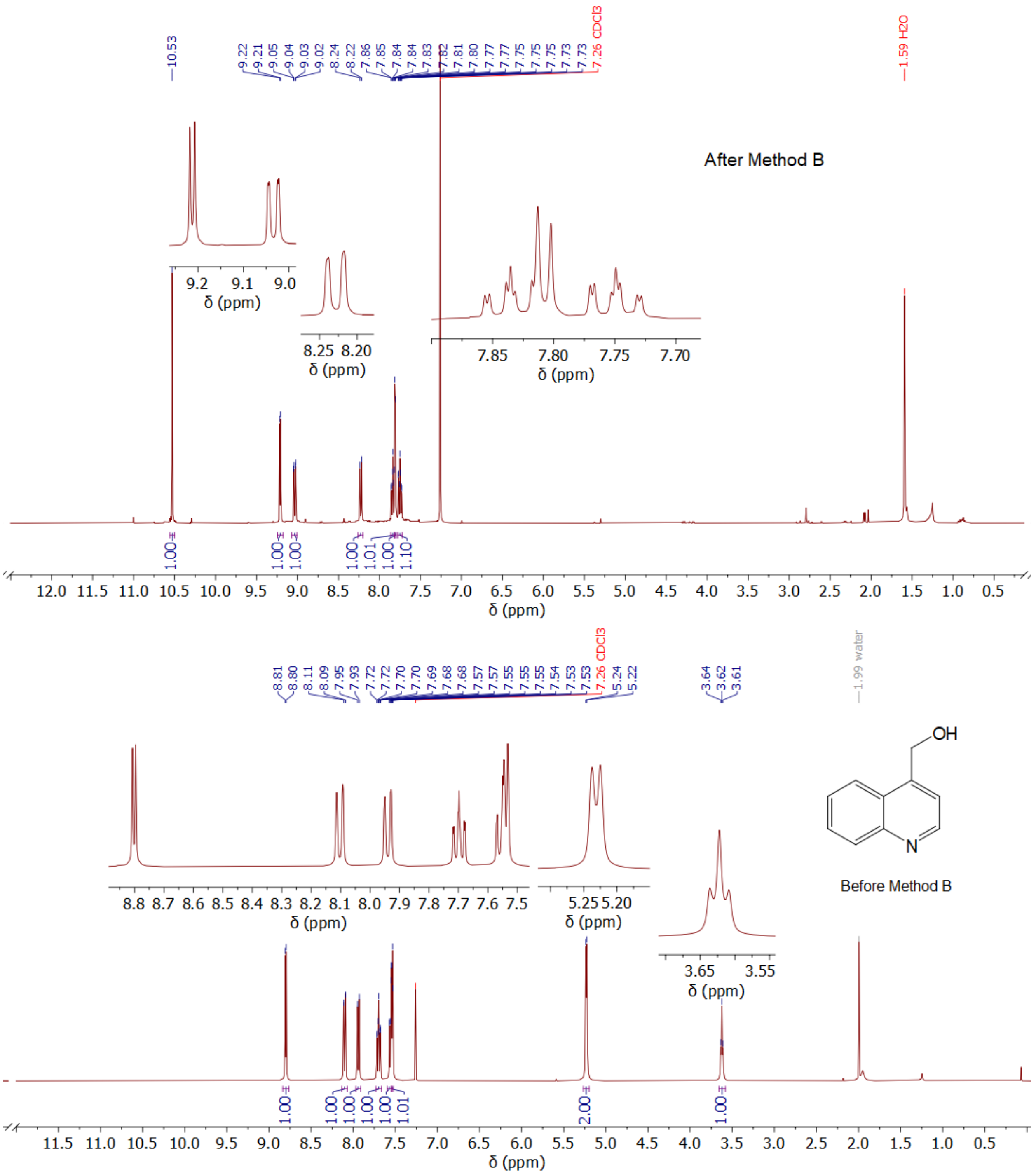


**Figure S23.** Comparison of $^{1}$H NMR spectra ($CDCl_3$) of 4-QuMeOH before and after stirring for 7 h under the conditions of Method B, showing quantitative conversion to 4-QuCHO (**11**).

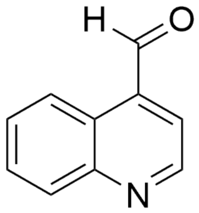

**4-Quinolinecarboxaldehyde (11).** $^{1}$H NMR (400 MHz, $CDCl_3$) δ 10.53 (s, 1H), 9.21 (d, *J* = 4.2 Hz, 1H), 9.03 (dd, *J* = 8.5, 1.5 Hz, 1H), 8.23 (d, *J* = 8.4 Hz, 1H), 7.86 – 7.81 (m, 1H), 7.81 (d, *J* = 4.3 Hz, 1H), 7.75 (ddd, *J* = 8.3, 6.9, 1.4 Hz, 1H). LCMS (ESI) m/z for $C_{10}H_7NO$ $[M+H]^+$: calculated: 158.052; found 158.05.

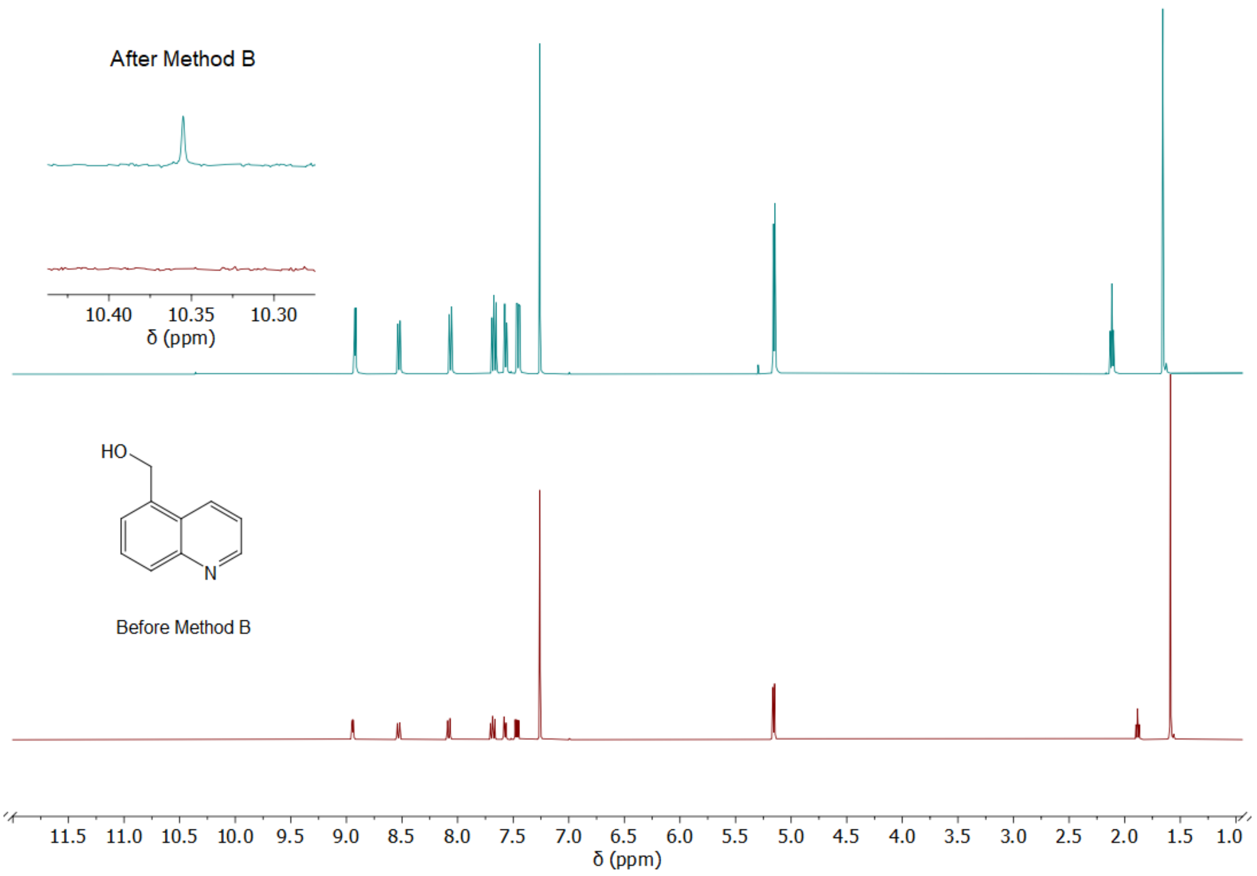


**Figure S24.** Comparison of $^{1}H$ NMR spectra ($CDCl_3$) of 5-QuMeOH before and after stirring for 7 h under the conditions of Method B, showing <1% conversion.

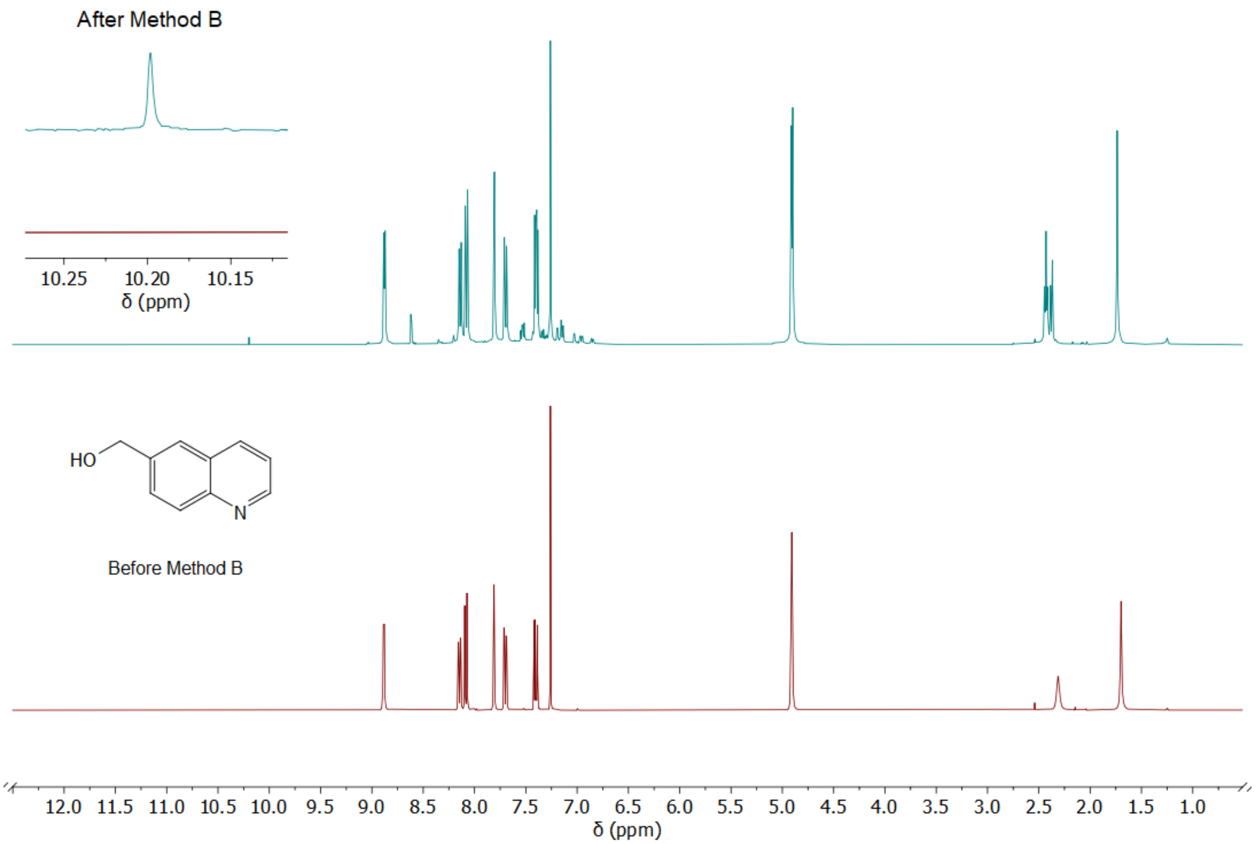


**Figure S25.** Comparison of $^{1}$H NMR spectra ($CDCl_3$) of 6-QuMeOH before and after stirring for 7 h under the conditions of Method B, showing <1% conversion.

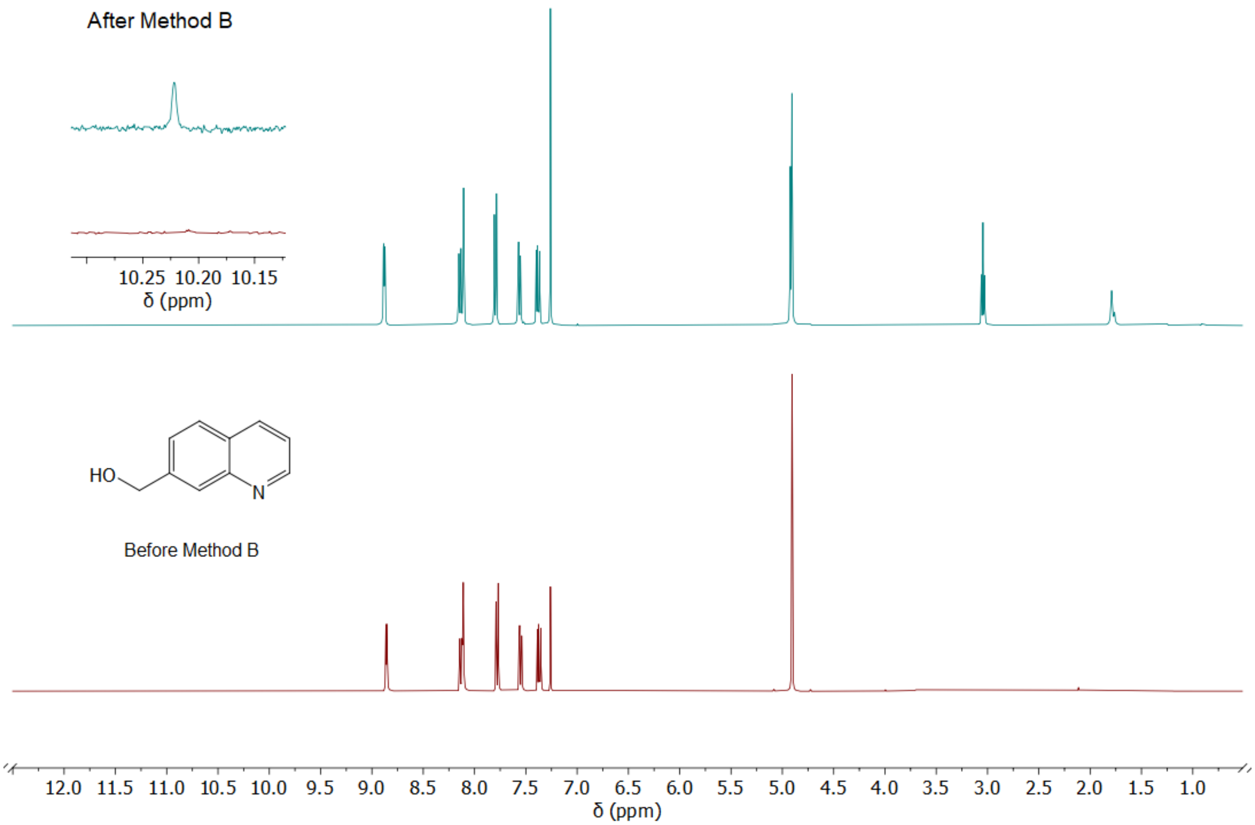


**Figure S26.** Comparison of $^1$H NMR spectra ($CDCl_3$) of 7-QuMeOH before and after stirring for 7 h under the conditions of Method B, showing <1% conversion.

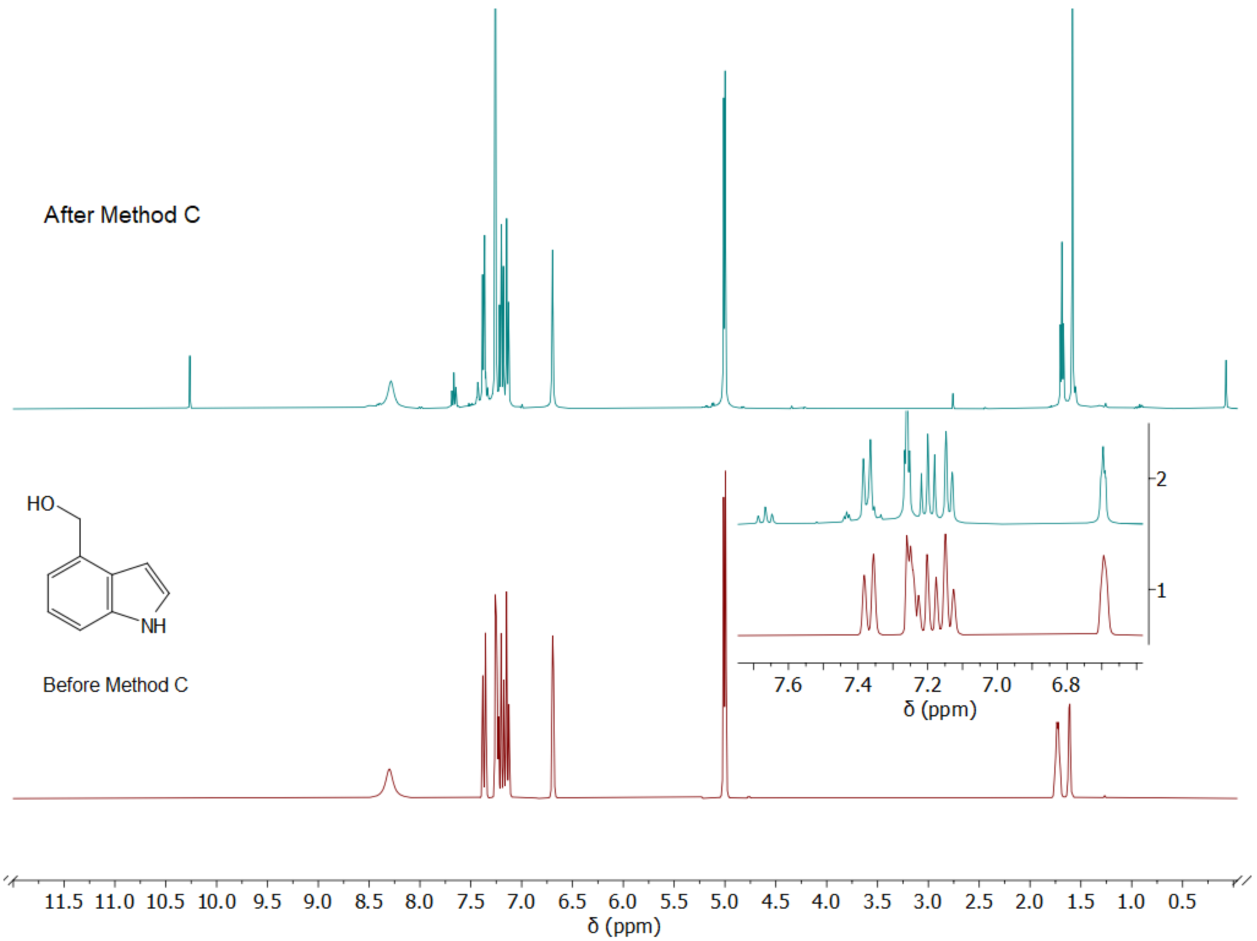


**Figure S27.** Comparison of $^{1}H$ NMR spectra ($CDCl_3$) of 4-InMeOH before and after stirring for 7 h under the conditions of Method C, showing 18% conversion.

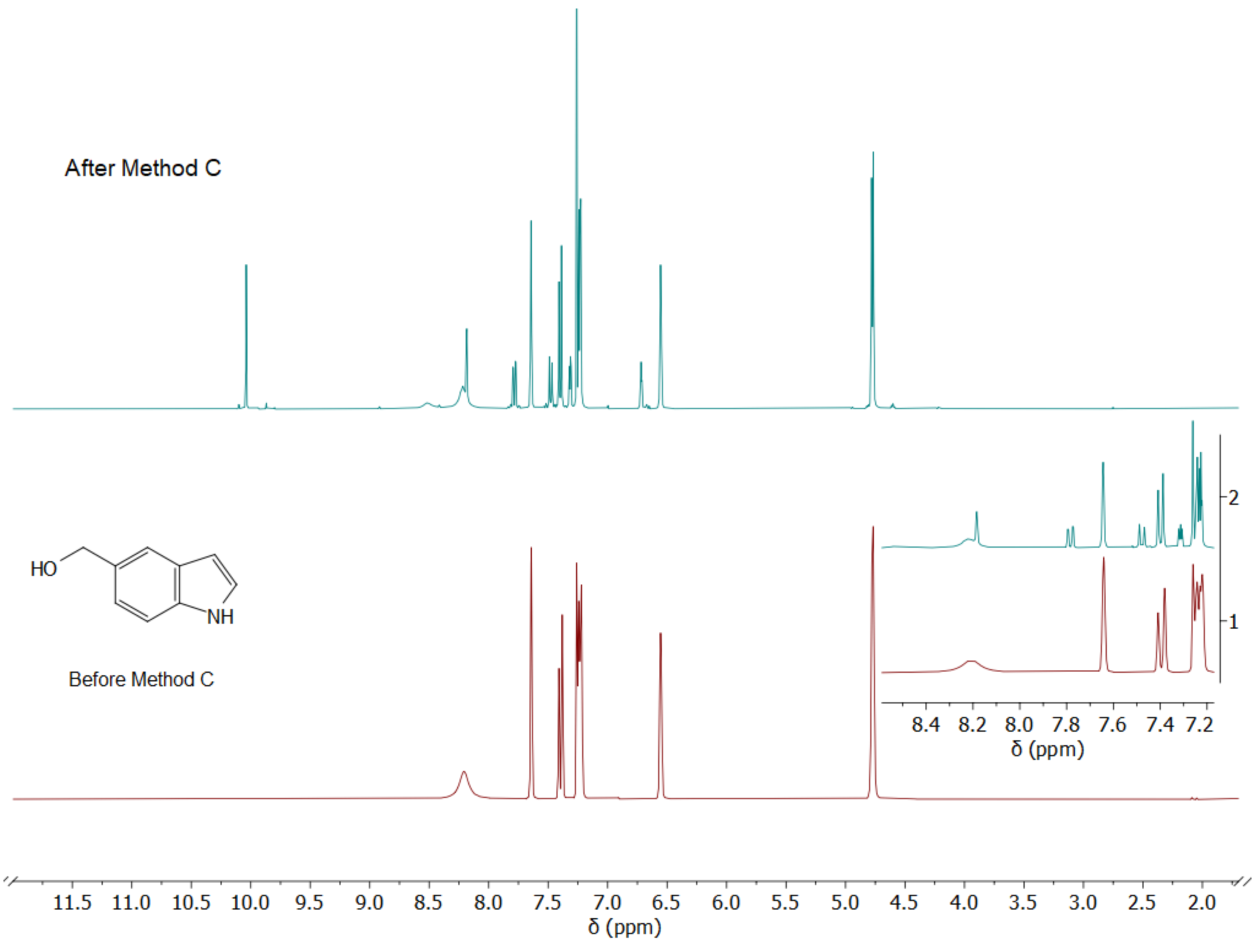


**Figure S28.** Comparison of $^1$H NMR spectra ($CDCl_3$) of 5-InMeOH before and after stirring for 7 h under the conditions of Method C, showing 44% conversion.

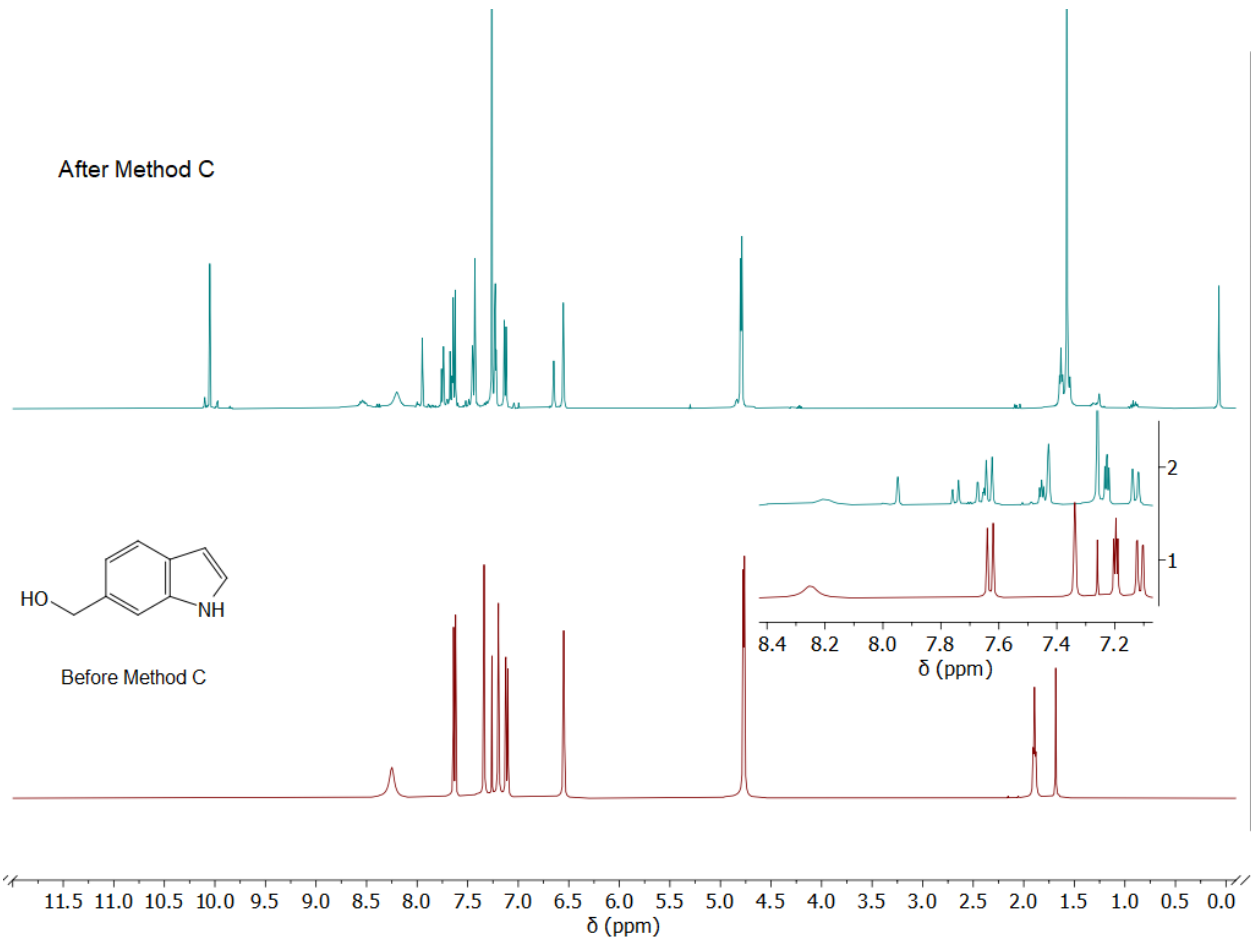


**Figure S29.** Comparison of $^{1}$H NMR spectra ($CDCl_3$) of 6-InMeOH before and after stirring for 7 h under the conditions of Method C, showing 34% conversion.

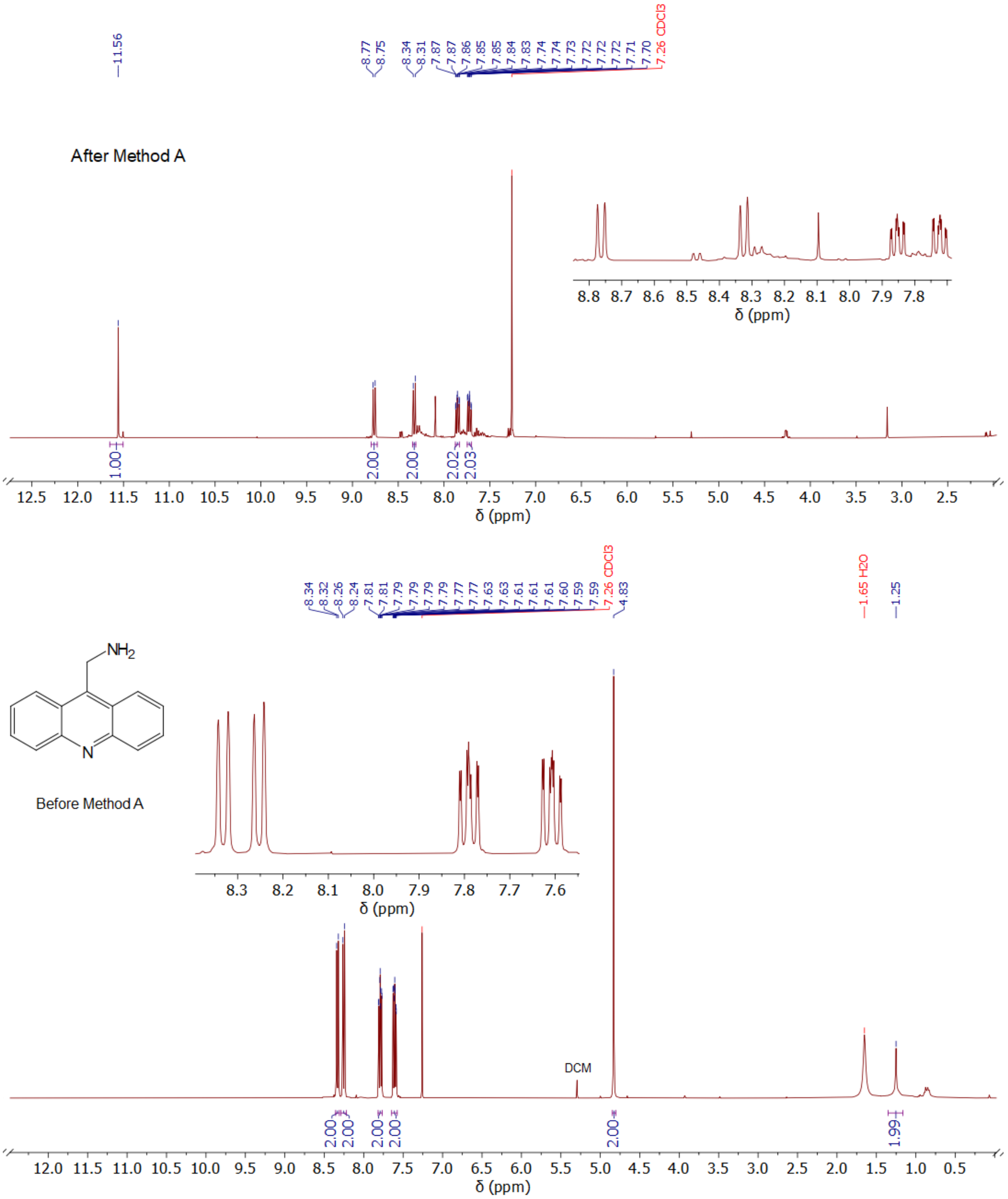


**Figure S30.** Comparison of $^{1}$H NMR spectra ($CDCl_3$) of 9-AcMeNH$_2$ (**8**) before and after refluxing for 2 h under the conditions of Method A, showing quantitative conversion to **3**.

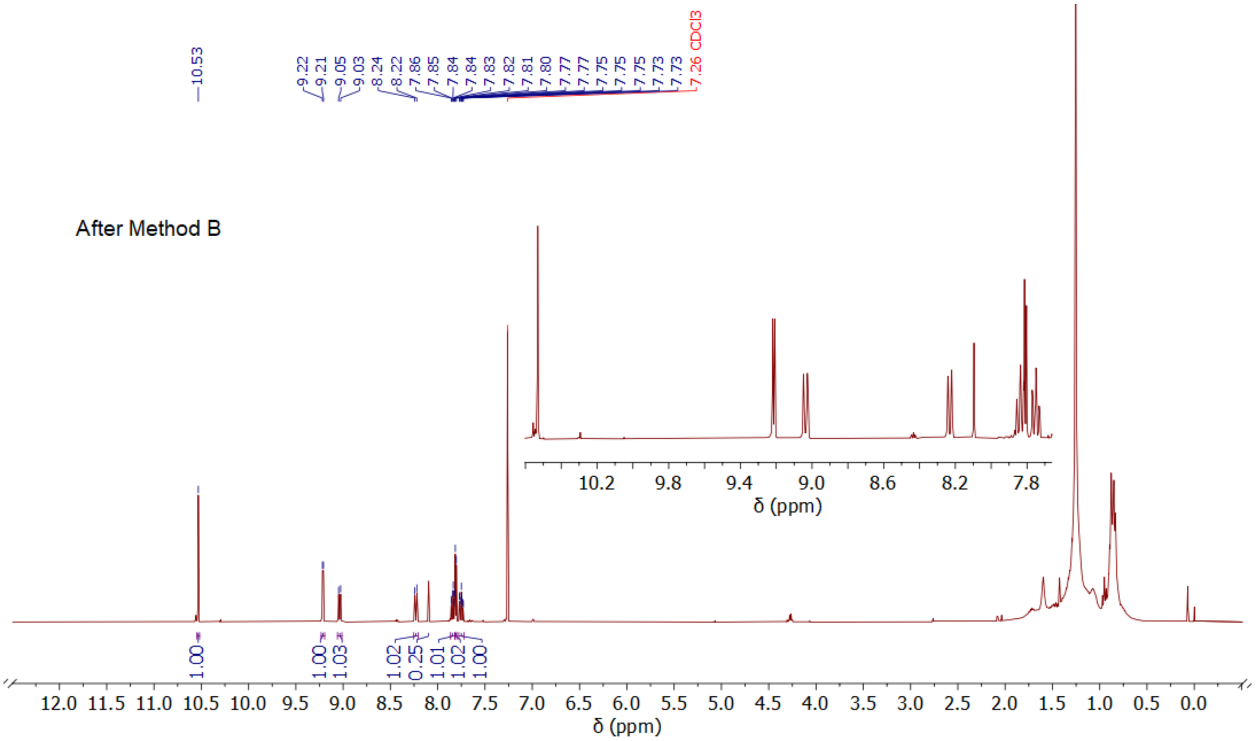


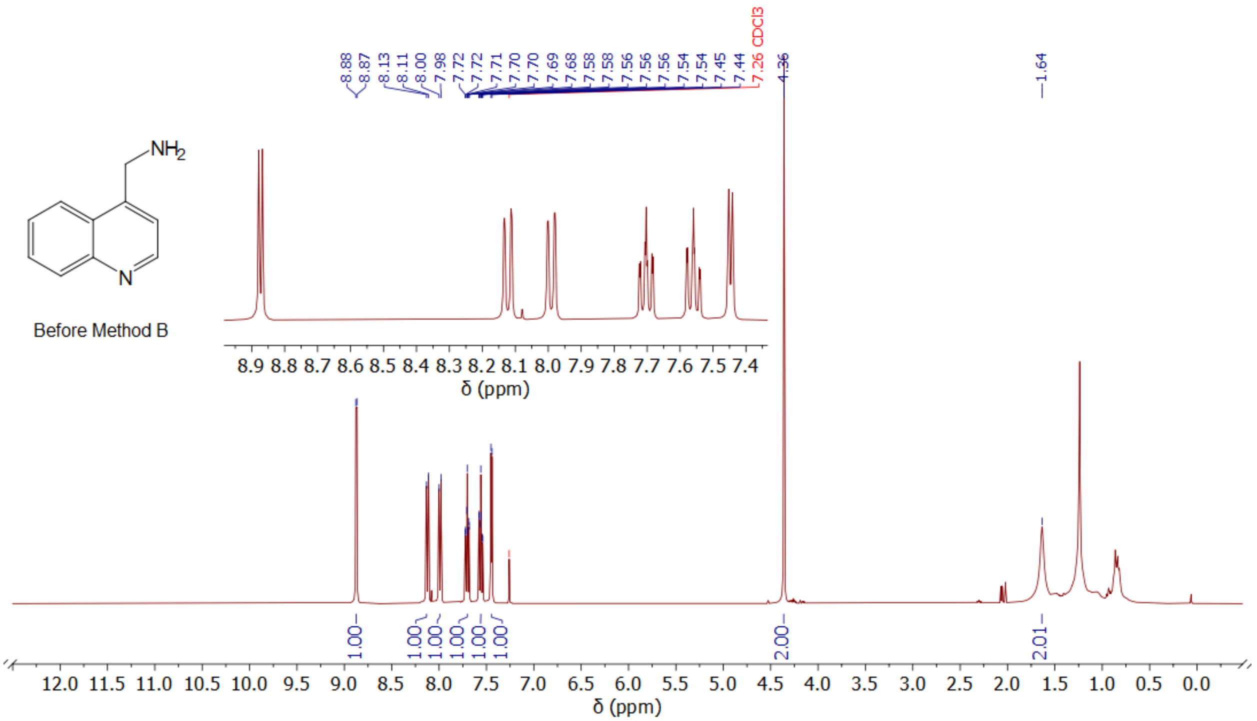


**Figure S31.** Comparison of $^{1}H$ NMR spectra ($CDCl_3$) of 4-QuMeNH$_2$ before and after stirring for 4 h under the conditions of Method B, showing quantitative conversion to **11**.

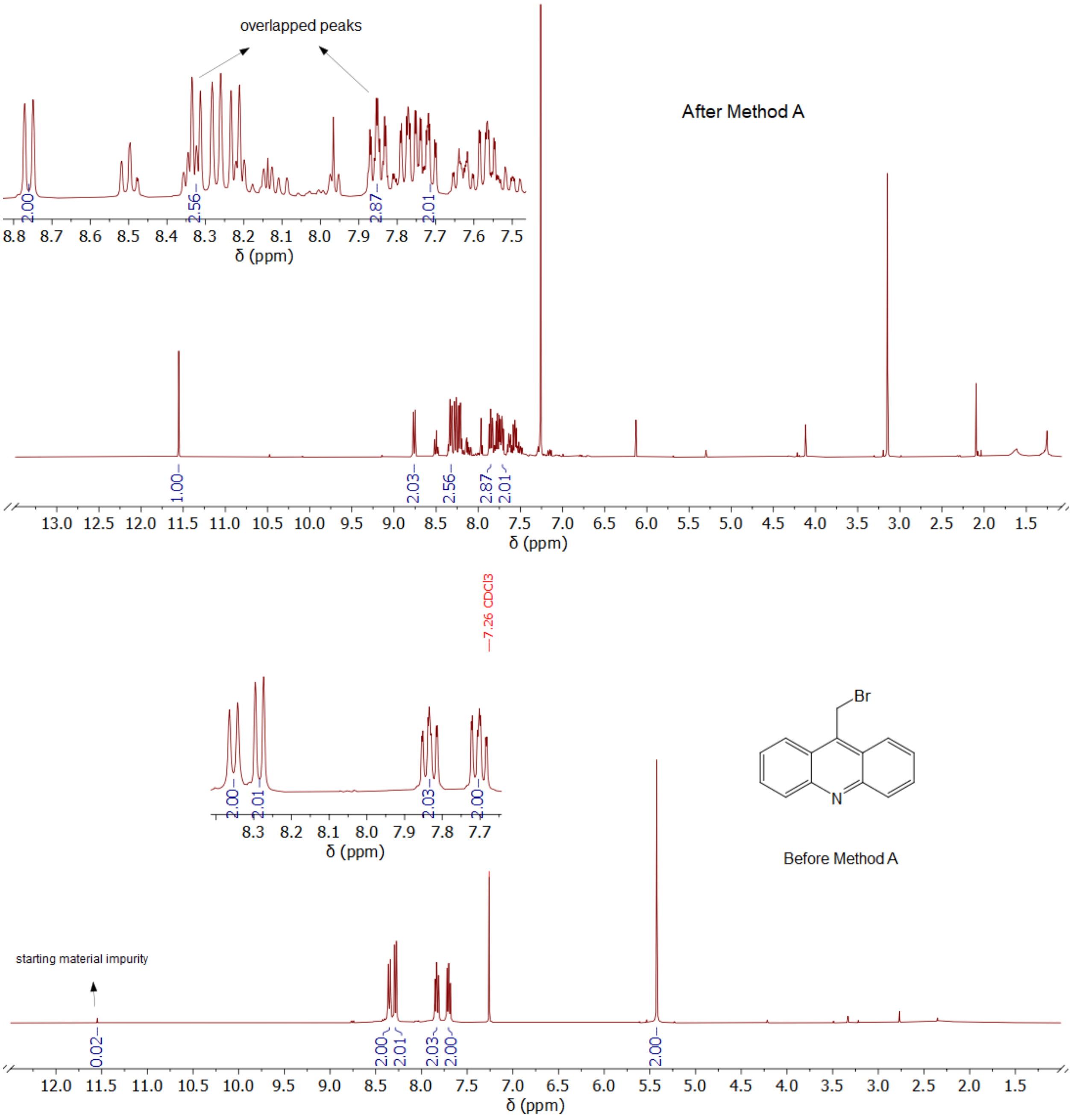


**Figure S32.** Comparison of $^{1}$H NMR spectra ($CDCl_3$) of 9-AcMeBr before and after refluxing for 5 h under the conditions of Method A, showing formation of roughly equal amounts of **3** and 9-methylacridine.

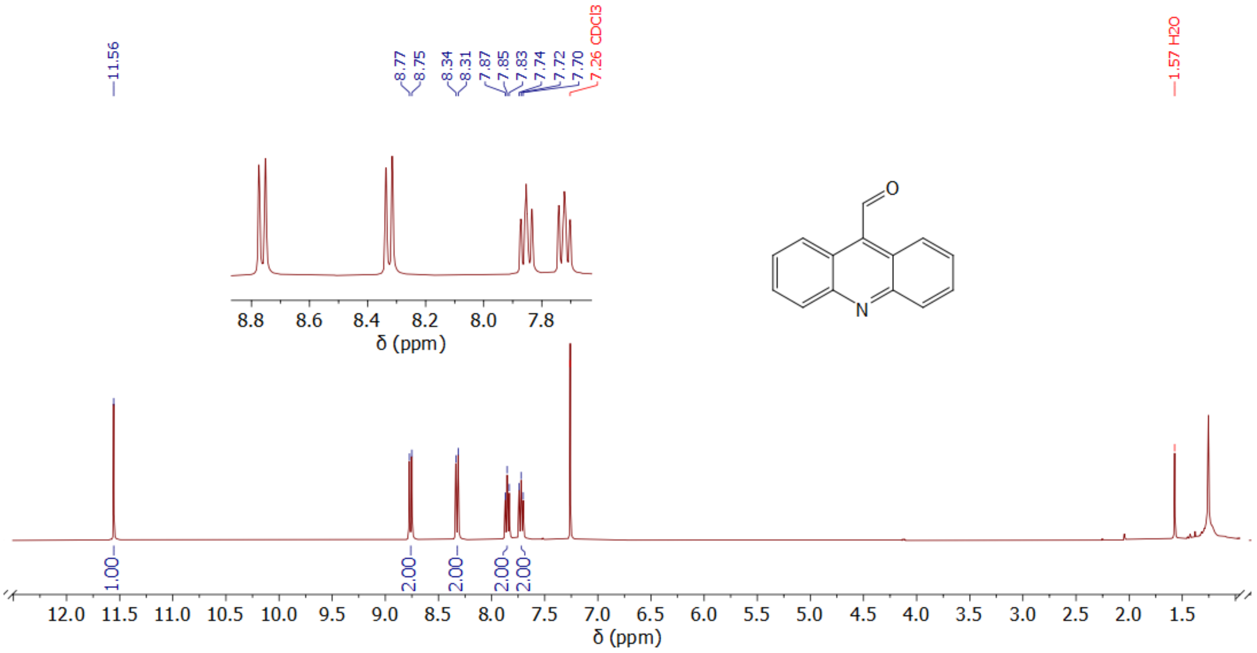


**Figure S33.** $^1$H NMR spectrum ($CDCl_3$) of **3** obtained from 9-AcMeBr under the conditions of Method A. 9-AcMeBr (200 mg, 0.735 mmol) was refluxed in 5% acidic solution (HOAc, v/v) for 5 h. Flash chromatography on silica gel with 9:1 DCM/ethyl acetate gave the product as a yellow powder (52 mg, 26%).

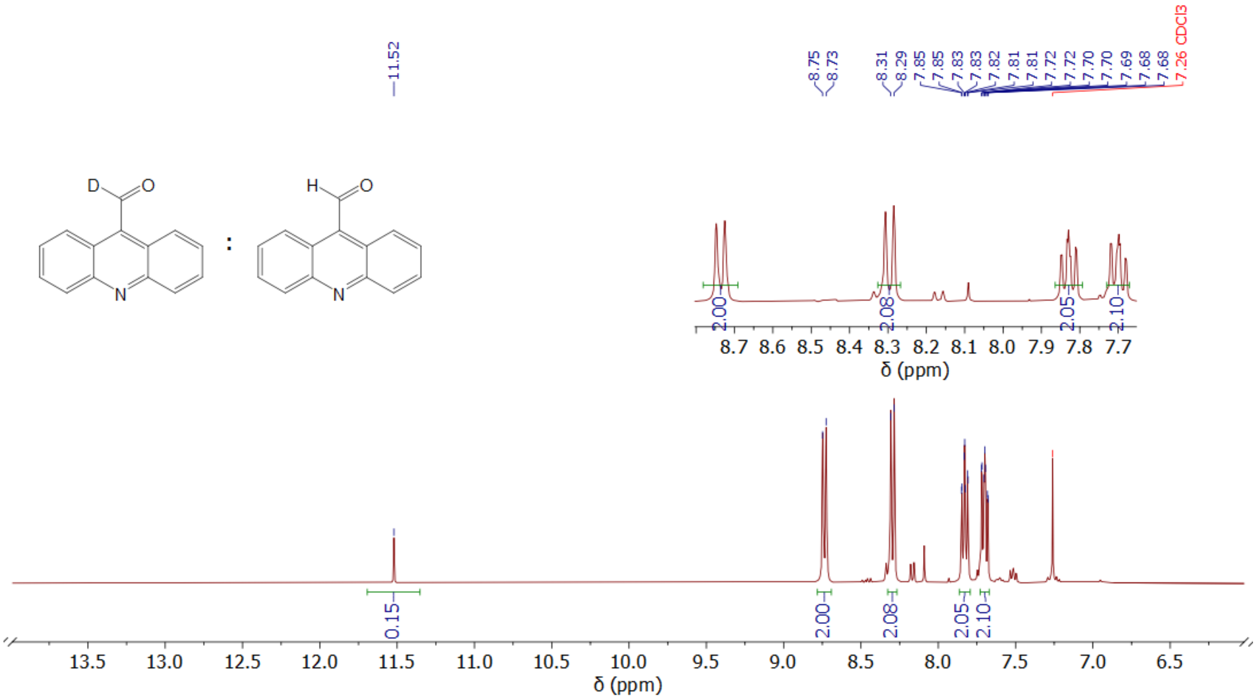


**Figure S34.** $^{1}$H NMR spectrum of crude reaction mixture following oxidation of 9-AcMeOH-*d* (**4**) in 5% (v/v) aqueous HOAc at room temperature for 5 days. All peaks match the spectrum of 9-AcCHO, but the aldehyde peak only integrates to 0.15, revealing a mixture of 9-AcCHO (15%) and 9-AcCHO-*d* (85%).

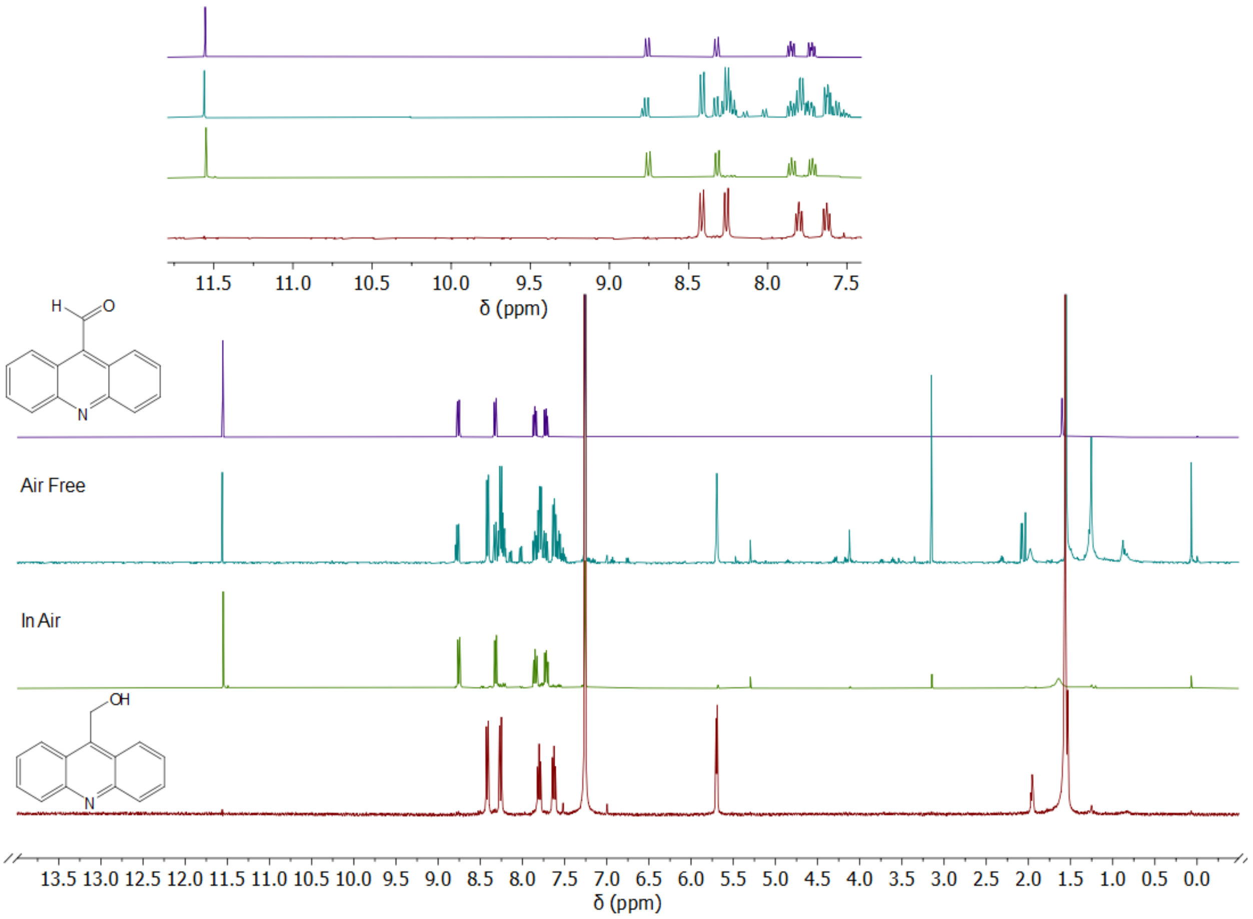


**Figure S35.** Crude $^1$H NMR spectra showing the oxidation of **2** to **3** after refluxing in 5% (v/v) aqueous HOAc for 2 h under different atmospheres. In air, the reaction proceeds nearly to completion, while only 28% conversion is achieved under argon. For comparison, the spectra of the pure starting material and product are shown at the bottom and top, respectively.

**Photochemical cascade synthesis of 9-AcCHO.** Acridine (167 mg, 0.93 mmol), selectfluor (1.30 g, 3.7 mmol), and trifluoroacetic acid (540 mg, 4.7 mmol) were combined in 14 mL of a 1:1 mixture of methanol and acetonitrile that had been rigorously sparged with argon. The reaction mixture was sealed in a 20 mL glass scintillation vial under argon with parafilm and stirred at room temperature under illumination by a 100 W, 385 nm LED array (Chanzon). The flux at the surface of the vial was ~1.7 W/cm$^2$. The LED was turned off after 5 h, and the vial cap was removed for 2 h to allow air to saturate the reaction mixture under vigorous stirring. The cap was loosely placed over the vial, and the reaction was stirred in the dark for an additional 22 h before it was diluted with 100 mL water, neutralized with aqueous NaOH, and extracted with DCM (3x100 mL). The combined organic layers were washed with brine (150 mL), dried over sodium sulfate, and evaporated under reduced pressure. Flash chromatography on silica gel with 1:1 DCM/ethyl acetate gave the product as a pale yellow powder (105 mg, 54%). The spectra of the crude product mixture and the starting material are shown in Figure S36.

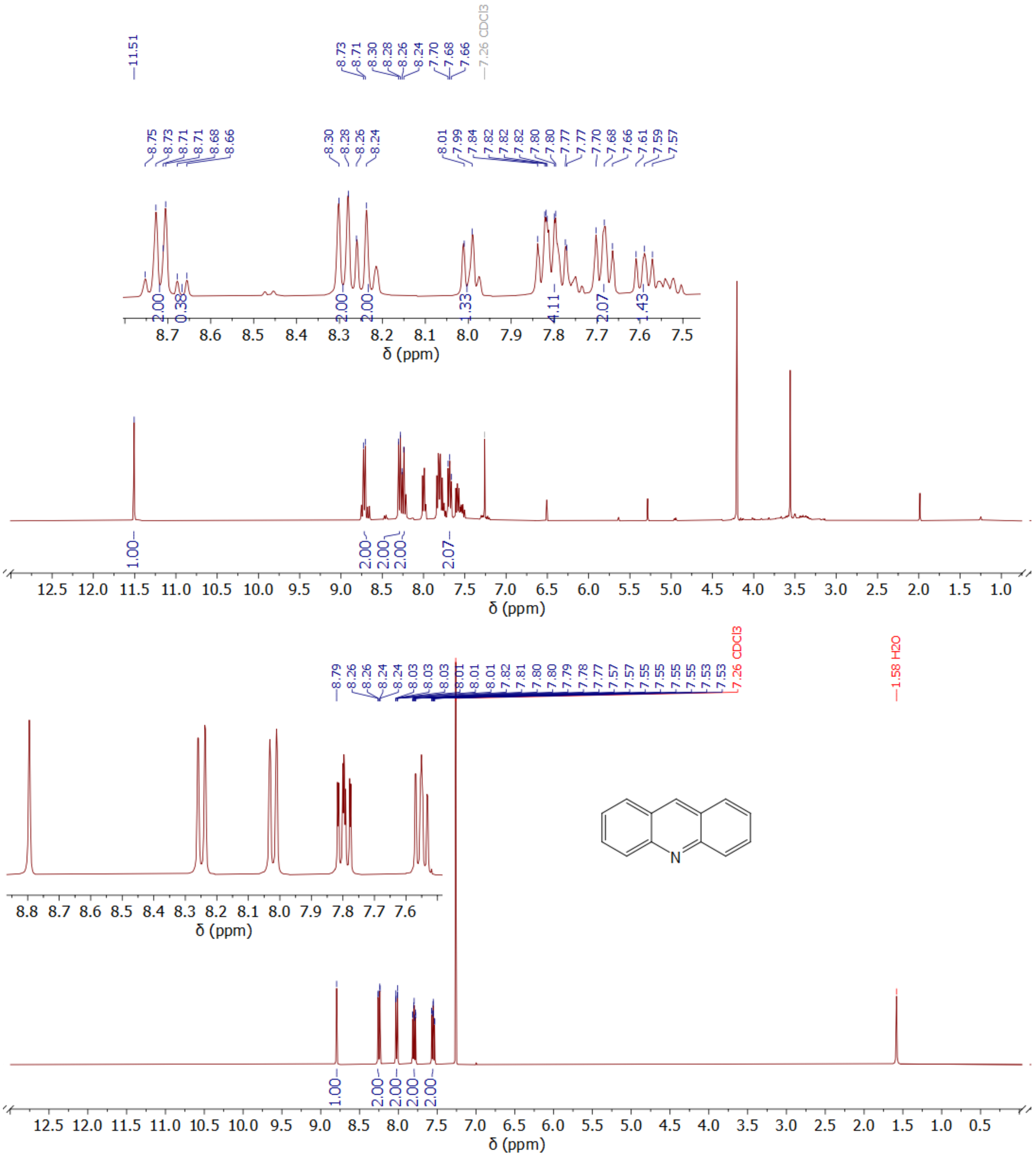


**Figure S36.** $^{1}$H NMR spectra ($CDCl_3$) of acridine (bottom) and crude product mixture (top) for the photochemical synthesis of 9-AcCHO.

**Iron-mediated one-pot synthesis of 9-AcCHO.** Acridine (90 mg, 0.50 mmol) and iron(II) perchlorate hydrate (300 mg) were combined in 4 mL of methanol and stirred vigorously for 10 min. A 30% aqueous solution of hydrogen peroxide (400 mg solution, 3.54 mmol $H_2O_2$) was then added dropwise over 3 min, and the reaction was stirred at room temperature for 1.5 h. The mixture was then diluted with 100 mL of 5% aqueous acetic acid and stirred under reflux for 3 h. After cooling to room temperature, the mixture was neutralized with aqueous NaOH and extracted with DCM (3x100 mL). The combined organic layers were washed with brine (150 mL), dried over sodium sulfate, and evaporated under reduced pressure. Flash chromatography on silica gel with 1:1 DCM/ethyl acetate gave the product as a pale yellow powder (28 mg, 27%). The spectra of the crude product mixture and the starting material are shown in Figure S37.

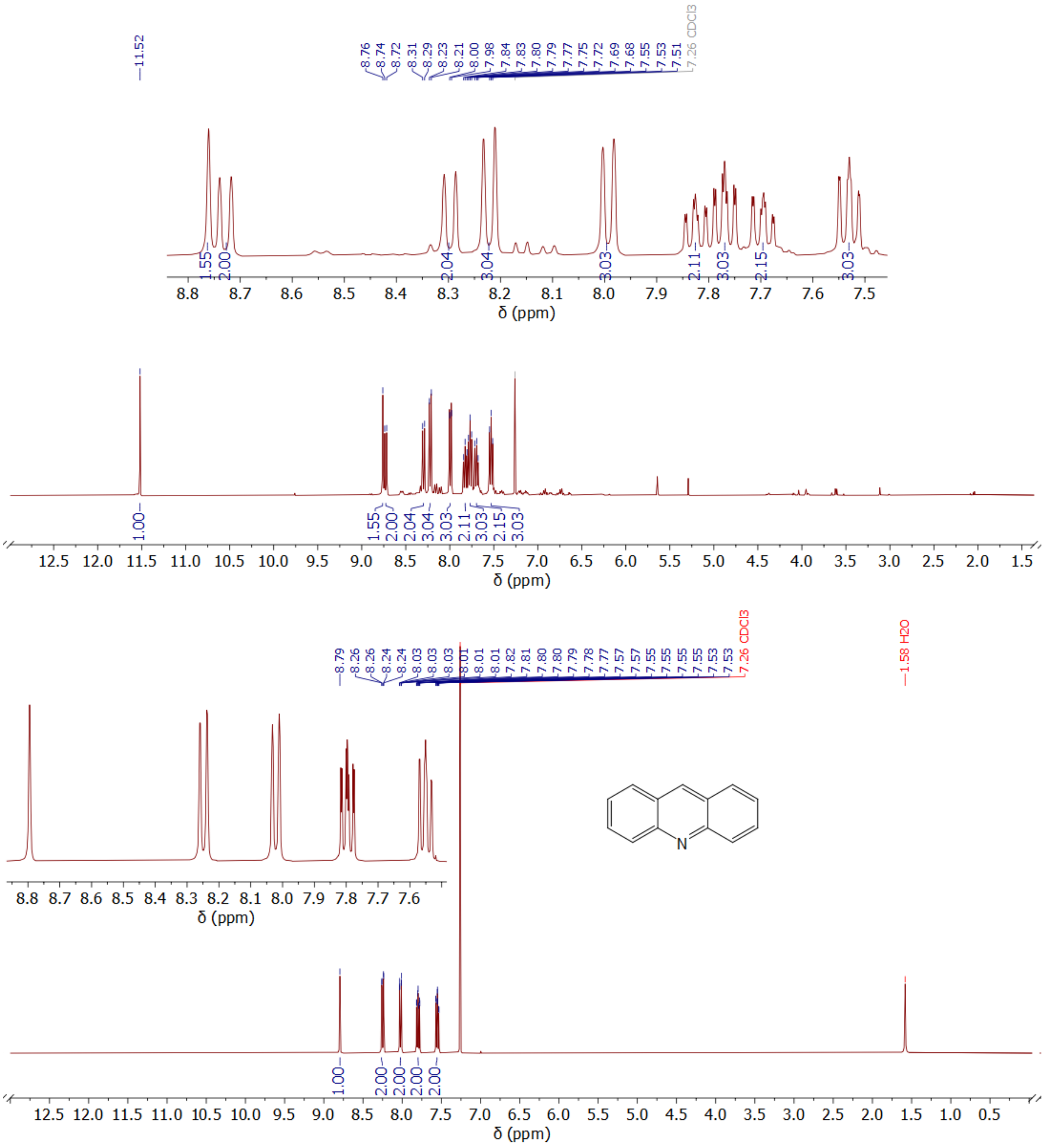


**Figure S37.** $^{1}H$ NMR spectra ($CDCl_3$) of acridine (bottom) and crude reaction mixture (top) for the iron-mediated synthesis of 9-AcCHO.

# Optimized geometry coordinates

### 1-AcMeOH, starting alcohol

| Atom | x | y | z |
|---|---|---|---|
| C | -3.776181 | 1.937887 | 0.215920 |
| C | -3.781295 | 0.521595 | 0.002358 |
| N | -2.608632 | 2.617463 | 0.052780 |
| C | -1.427893 | 2.050950 | -0.313781 |
| C | -1.398788 | 0.640943 | -0.545533 |
| C | -2.582055 | -0.087948 | -0.387055 |
| C | -5.060130 | -1.697628 | 0.035154 |
| O | -4.809553 | -2.027640 | -1.339691 |
| H | -4.819698 | -2.989320 | -1.427550 |
| C | -0.163147 | 0.041429 | -0.928032 |
| C | 0.961933 | 0.809218 | -1.067851 |
| C | 0.907800 | 2.209579 | -0.835111 |
| C | -0.261072 | 2.829006 | -0.461552 |
| H | -0.132865 | -1.028167 | -1.100066 |
| H | 1.901095 | 0.354025 | -1.357790 |
| H | 1.806192 | 2.803858 | -0.953081 |
| H | -0.300265 | 3.897491 | -0.284583 |
| C | -5.008386 | -0.199846 | 0.200128 |
| C | -6.132607 | 0.494292 | 0.572494 |
| C | -6.105897 | 1.899388 | 0.768252 |
| C | -4.951808 | 2.620384 | 0.596731 |
| H | -7.014253 | 2.410279 | 1.064551 |
| H | -4.933011 | 3.690989 | 0.755869 |
| H | -7.064095 | -0.038390 | 0.723799 |
| H | -2.621394 | 3.619962 | 0.219918 |
| H | -4.313168 | -2.172904 | 0.681679 |
| H | -6.046233 | -2.051643 | 0.345650 |
| H | -2.563681 | -1.154307 | -0.576190 |

**1-AcMeOH, enol tautomer**

| Atom | x | y | z |
|---|---|---|---|
| C | -3.941693 | 1.967740 | -0.071231 |
| C | -3.879060 | 0.568829 | -0.073831 |
| N | -2.797633 | 2.661793 | -0.715991 |
| C | -1.482091 | 2.014546 | -0.484912 |
| C | -1.483340 | 0.568840 | -0.609984 |
| C | -2.668911 | -0.111525 | -0.488018 |
| C | 0.105798 | -1.322300 | -1.126380 |
| O | -0.857242 | -2.220049 | -1.363741 |
| H | -2.774866 | 3.644492 | -0.434116 |
| H | -2.949843 | 2.650690 | -1.733664 |
| H | -0.474153 | -3.059333 | -1.654801 |
| H | 1.129133 | -1.666574 | -1.232811 |
| C | -0.145258 | -0.023356 | -0.777826 |
| C | 1.014132 | 0.819719 | -0.569112 |
| C | 0.914725 | 2.147566 | -0.309354 |
| C | -0.379196 | 2.774195 | -0.314568 |
| H | 1.991222 | 0.353127 | -0.637841 |
| H | 1.795950 | 2.755899 | -0.153551 |
| H | -0.458826 | 3.851643 | -0.220766 |
| C | -5.006649 | -0.108180 | 0.425636 |
| C | -6.103216 | 0.596908 | 0.911329 |
| C | -6.111154 | 1.993896 | 0.916730 |
| C | -5.014451 | 2.693713 | 0.408669 |
| H | -6.964115 | 2.537562 | 1.302585 |
| H | -5.008613 | 3.777948 | 0.387005 |
| H | -5.003920 | -1.192326 | 0.445006 |
| H | -6.958797 | 0.053987 | 1.296755 |
| H | -2.696503 | -1.187812 | -0.559316 |

**1-AcMeOH, dihydro tautomer**

| Atom | x | y | z |
|---|---|---|---|
| C | -6.205494 | 0.491301 | 0.754353 |
| C | -6.207667 | 1.776202 | 0.212187 |
| C | -5.010748 | 2.351725 | -0.218726 |
| C | -3.840478 | 1.624957 | -0.094130 |
| C | -3.795747 | 0.344715 | 0.463594 |
| C | -5.012932 | -0.213723 | 0.884462 |
| N | -2.572602 | 2.219933 | -0.624633 |
| C | -1.283757 | 1.575773 | -0.212562 |
| C | -1.344911 | 0.250777 | 0.364452 |
| C | -2.528895 | -0.335395 | 0.654652 |
| C | -0.140079 | 2.253035 | -0.416477 |
| C | 1.137036 | 1.654066 | -0.057571 |
| C | 1.204442 | 0.416097 | 0.450499 |
| C | -0.020519 | -0.443403 | 0.666850 |
| H | -7.130441 | 2.336089 | 0.119464 |
| H | -5.009123 | -1.206131 | 1.321229 |
| H | -7.133740 | 0.040228 | 1.084572 |
| C | 0.133275 | -1.715961 | -0.184575 |
| H | 2.033998 | 2.245071 | -0.196287 |
| H | 2.156008 | -0.021926 | 0.728124 |
| H | -5.008468 | 3.350330 | -0.639964 |
| H | -0.148684 | 3.252802 | -0.835056 |
| O | 0.513385 | -2.768368 | 0.266388 |
| H | -2.553508 | 3.206124 | -0.350960 |
| H | -0.117459 | -1.604609 | -1.254923 |
| H | -0.018012 | -0.783697 | 1.707016 |
| H | -2.622184 | 2.210944 | -1.650308 |
| H | -2.554737 | -1.320422 | 1.107782 |

**3-AcMeOH, starting alcohol**

| Atom | x | y | z |
|---|---|---|---|
| C | -3.793495 | 1.875210 | 0.235139 |
| C | -3.809451 | 0.541093 | -0.283847 |
| N | -2.597552 | 2.521841 | 0.318775 |
| C | -1.402959 | 1.982458 | -0.050651 |
| C | -1.386725 | 0.646734 | -0.562585 |
| C | -2.600494 | -0.043105 | -0.668675 |
| C | -7.466379 | 2.519893 | 0.959309 |
| O | -8.583859 | 1.653495 | 1.107732 |
| H | -8.500746 | 1.167017 | 1.936783 |
| C | -0.135321 | 0.077592 | -0.937839 |
| C | 1.019802 | 0.802660 | -0.809971 |
| C | 0.979177 | 2.128589 | -0.303497 |
| C | -0.205289 | 2.716600 | 0.073147 |
| H | -0.119452 | -0.936079 | -1.321221 |
| H | 1.971162 | 0.368883 | -1.092740 |
| H | 1.901426 | 2.690576 | -0.211492 |
| H | -0.231841 | 3.730045 | 0.456163 |
| C | -5.064389 | -0.127923 | -0.372601 |
| C | -6.214404 | 0.495921 | 0.028031 |
| C | -6.182996 | 1.819857 | 0.550522 |
| C | -4.983511 | 2.494335 | 0.656186 |
| H | -4.946409 | 3.501736 | 1.055911 |
| H | -7.167011 | -0.013235 | -0.042464 |
| H | -2.592019 | 3.459466 | 0.712581 |
| H | -2.599822 | -1.059011 | -1.049951 |
| H | -7.294569 | 3.093849 | 1.875599 |
| H | -7.738630 | 3.230961 | 0.173531 |
| H | -5.087471 | -1.138898 | -0.762723 |

**3-AcMeOH, enol tautomer**

| Atom | x | y | z |
|---|---|---|---|
| C | -3.903125 | 1.967594 | -0.020653 |
| C | -3.883108 | 0.565755 | -0.044292 |
| N | -2.756336 | 2.668315 | -0.661936 |
| C | -1.449184 | 1.960938 | -0.585562 |
| C | -1.508983 | 0.516236 | -0.650665 |
| C | -2.694551 | -0.142362 | -0.467693 |
| C | 2.110534 | 2.731768 | -0.615427 |
| O | 3.319869 | 2.164636 | -0.707393 |
| H | -2.669454 | 3.612418 | -0.270048 |
| H | -2.990446 | 2.802830 | -1.656057 |
| H | 4.014673 | 2.835968 | -0.689448 |
| H | 2.071793 | 3.809969 | -0.508356 |
| C | -0.228160 | -0.139610 | -0.825224 |
| C | 0.936364 | 0.551412 | -0.818114 |
| C | 0.962683 | 1.992535 | -0.655262 |
| C | -0.306287 | 2.674700 | -0.560921 |
| H | 1.877992 | 0.027349 | -0.927376 |
| H | -0.327333 | 3.757885 | -0.501580 |
| C | -5.033064 | -0.082256 | 0.443526 |
| C | -6.111935 | 0.646925 | 0.933568 |
| C | -6.079006 | 2.042805 | 0.959522 |
| C | -4.957937 | 2.714982 | 0.467932 |
| H | -6.917978 | 2.607400 | 1.346937 |
| H | -4.925042 | 3.798907 | 0.455472 |
| H | -5.062304 | -1.166205 | 0.446968 |
| H | -6.985635 | 0.123757 | 1.304726 |
| H | -2.725500 | -1.224755 | -0.509242 |
| H | -0.222500 | -1.218783 | -0.930672 |

**3-AcMeOH, dihydro tautomer**

| Atom | x | y | z |
|---|---|---|---|
| C | -6.226164 | 0.442676 | 0.809515 |
| C | -6.252763 | 1.693153 | 0.193003 |
| C | -5.065957 | 2.271703 | -0.261594 |
| C | -3.880107 | 1.581326 | -0.087143 |
| C | -3.811362 | 0.330139 | 0.533305 |
| C | -5.020325 | -0.229517 | 0.978877 |
| N | -2.621603 | 2.189855 | -0.626850 |
| C | -1.316852 | 1.572992 | -0.210206 |
| C | -1.362040 | 0.260197 | 0.417293 |
| C | -2.538163 | -0.329423 | 0.738089 |
| C | -0.193129 | 2.242809 | -0.473655 |
| C | 1.153706 | 1.675569 | -0.121554 |
| C | 1.090527 | 0.293170 | 0.458514 |
| C | -0.064722 | -0.340610 | 0.697688 |
| H | -7.188146 | 2.222693 | 0.056680 |
| H | -4.996091 | -1.198221 | 1.465050 |
| H | -7.146814 | -0.009295 | 1.159175 |
| C | 2.122566 | 1.789969 | -1.307976 |
| H | 2.034307 | -0.187012 | 0.685499 |
| H | -5.095737 | 3.232108 | -0.761747 |
| H | -0.232310 | 3.215592 | -0.952795 |
| O | 2.894479 | 0.924642 | -1.636945 |
| H | -2.618157 | 3.177614 | -0.358109 |
| H | 2.086910 | 2.757885 | -1.843685 |
| H | -2.670813 | 2.170181 | -1.652010 |
| H | -2.540086 | -1.306524 | 1.207902 |
| H | -0.065463 | -1.335853 | 1.129344 |
| H | 1.612411 | 2.360585 | 0.619553 |

**9-AcMeOH, starting alcohol**

| **Atom** | **x** | **y** | **z** |
|---|---|---|---|
| C | -3.783233 | 1.918153 | 0.193000 |
| C | -3.771671 | 0.495099 | 0.027539 |
| N | -2.620259 | 2.601058 | 0.039068 |
| C | -1.433831 | 2.029651 | -0.288971 |
| C | -1.376803 | 0.608668 | -0.458949 |
| C | -2.550185 | -0.152405 | -0.262165 |
| C | -2.503591 | -1.661681 | -0.382565 |
| O | -2.782078 | -1.995953 | -1.746626 |
| H | -2.704840 | -2.954155 | -1.842033 |
| C | -0.114503 | 0.045080 | -0.820417 |
| C | 0.988220 | 0.838903 | -0.987236 |
| C | 0.904762 | 2.242246 | -0.792393 |
| C | -0.284413 | 2.833739 | -0.448485 |
| H | -0.031255 | -1.021471 | -0.972791 |
| H | 1.937107 | 0.395891 | -1.265024 |
| H | 1.789684 | 2.854919 | -0.916900 |
| H | -0.359786 | 3.905057 | -0.302213 |
| C | -5.017248 | -0.190206 | 0.167213 |
| C | -6.167130 | 0.492392 | 0.460433 |
| C | -6.142722 | 1.899191 | 0.642089 |
| C | -4.974463 | 2.606899 | 0.509364 |
| H | -7.059203 | 2.424045 | 0.884117 |
| H | -4.959210 | 3.681357 | 0.645578 |
| H | -5.055828 | -1.259482 | 0.016105 |
| H | -7.104581 | -0.043093 | 0.549830 |
| H | -2.636564 | 3.606645 | 0.188086 |
| H | -1.521744 | -2.030397 | -0.080374 |
| H | -3.236023 | -2.115882 | 0.288880 |

**9-AcMeOH, enol tautomer**

| Atom | x | y | z |
|---|---|---|---|
| C | -3.875782 | 1.839711 | -0.184389 |
| C | -3.801879 | 0.445167 | -0.130951 |
| N | -2.757705 | 2.554124 | -0.858670 |
| C | -1.421359 | 1.971567 | -0.569718 |
| C | -1.342788 | 0.577604 | -0.530102 |
| C | -2.583694 | -0.218503 | -0.635684 |
| C | -2.547479 | -1.457319 | -1.173020 |
| O | -3.634425 | -2.248039 | -1.277169 |
| H | -2.779346 | 3.541717 | -0.593475 |
| H | -2.902813 | 2.521940 | -1.875941 |
| H | -3.408758 | -3.063650 | -1.740868 |
| H | -1.623453 | -1.863651 | -1.569003 |
| C | -0.064893 | 0.031263 | -0.329823 |
| C | 1.052117 | 0.851573 | -0.205870 |
| C | 0.927597 | 2.241554 | -0.247422 |
| C | -0.329125 | 2.813192 | -0.431550 |
| H | 0.049071 | -1.042652 | -0.243551 |
| H | 2.026544 | 0.402348 | -0.052939 |
| H | 1.797187 | 2.876487 | -0.132812 |
| H | -0.452045 | 3.889696 | -0.467570 |
| C | -4.884346 | -0.213235 | 0.476334 |
| C | -5.957231 | 0.506960 | 0.994854 |
| C | -5.989517 | 1.900219 | 0.920557 |
| C | -4.932134 | 2.579813 | 0.320494 |
| H | -6.826453 | 2.455272 | 1.325392 |
| H | -4.934271 | 3.661814 | 0.247089 |
| H | -4.871920 | -1.291059 | 0.555012 |
| H | -6.772810 | -0.024693 | 1.471808 |

**9-AcMeOH, dihydro tautomer**

| Atom | x | y | z |
|---|---|---|---|
| C | -6.138385 | 0.460986 | 0.814128 |
| C | -6.131712 | 1.780513 | 0.359984 |
| C | -4.954638 | 2.336820 | -0.128196 |
| C | -3.805628 | 1.555759 | -0.158571 |
| C | -3.775727 | 0.241548 | 0.292267 |
| C | -4.972649 | -0.296494 | 0.783127 |
| N | -2.568777 | 2.179456 | -0.704239 |
| C | -1.279026 | 1.562761 | -0.287129 |
| C | -1.257797 | 0.252130 | 0.174689 |
| C | -2.511483 | -0.590645 | 0.268484 |
| C | -0.138532 | 2.350464 | -0.378573 |
| C | 1.083614 | 1.805112 | 0.001238 |
| C | 1.141458 | 0.490849 | 0.468702 |
| C | -0.016287 | -0.274150 | 0.554751 |
| H | -7.034051 | 2.378894 | 0.389534 |
| H | -4.981800 | -1.317310 | 1.148981 |
| H | -7.050611 | 0.023605 | 1.201824 |
| C | -2.561677 | -1.568691 | -0.915869 |
| H | 1.981707 | 2.406726 | -0.062659 |
| H | 2.089819 | 0.063910 | 0.772978 |
| H | 0.032033 | -1.290387 | 0.929782 |
| H | -4.931281 | 3.362730 | -0.476998 |
| H | -0.201298 | 3.371892 | -0.734942 |
| O | -2.561934 | -2.767413 | -0.783665 |
| H | -2.564764 | 3.165191 | -0.424689 |
| H | -2.595791 | -1.104207 | -1.918639 |
| H | -2.467882 | -1.200016 | 1.173154 |
| H | -2.620136 | 2.190163 | -1.730851 |

**2-QuMeOH, starting alcohol**

| Atom | x | y | z |
|---|---|---|---|
| C | -3.691890 | 1.844523 | 0.186153 |
| C | -3.756482 | 0.435320 | 0.015314 |
| N | -2.488155 | 2.464419 | -0.060972 |
| C | -1.382062 | 1.831222 | -0.443889 |
| C | -1.410643 | 0.440062 | -0.625843 |
| C | -2.583195 | -0.242752 | -0.399525 |
| C | -0.146146 | 2.662643 | -0.637522 |
| O | -0.516735 | 4.030643 | -0.599937 |
| H | 0.256601 | 4.570582 | -0.400135 |
| C | -4.988306 | -0.218332 | 0.265806 |
| C | -6.085278 | 0.506936 | 0.669642 |
| C | -5.995997 | 1.907837 | 0.838578 |
| C | -4.815534 | 2.579944 | 0.600430 |
| H | -6.868233 | 2.462104 | 1.164516 |
| H | -4.745976 | 3.651052 | 0.747542 |
| H | -5.048143 | -1.292892 | 0.135446 |
| H | -7.026061 | 0.006231 | 0.865241 |
| H | -2.411962 | 3.478052 | 0.038465 |
| H | 0.557608 | 2.410665 | 0.164694 |
| H | 0.321841 | 2.391799 | -1.590757 |
| H | -0.512196 | -0.070032 | -0.948430 |
| H | -2.617870 | -1.317294 | -0.541960 |

**2-QuMeOH, enol tautomer**

| Atom | x | y | z |
|---|---|---|---|
| C | -3.715402 | 1.583556 | 0.104915 |
| C | -3.471647 | 0.185177 | -0.122596 |
| N | -2.599617 | 2.544060 | -0.158845 |
| C | -1.239361 | 1.967773 | -0.385266 |
| C | -1.103976 | 0.587206 | -0.560931 |
| C | -2.257448 | -0.294163 | -0.450173 |
| C | -4.870543 | 2.139405 | 0.511095 |
| O | -4.947507 | 3.475097 | 0.657424 |
| H | -2.552499 | 3.201444 | 0.628712 |
| H | -2.846261 | 3.127616 | -0.967538 |
| H | -4.322943 | -0.475333 | -0.005538 |
| H | -5.819305 | 3.750970 | 0.966408 |
| H | -5.746149 | 1.535457 | 0.715678 |
| C | 0.187353 | 0.105519 | -0.824973 |
| C | 1.272811 | 0.971950 | -0.909967 |
| C | 1.097292 | 2.343773 | -0.727530 |
| C | -0.176299 | 2.849684 | -0.464654 |
| H | 0.328292 | -0.960640 | -0.962783 |
| H | 2.259579 | 0.575976 | -1.117846 |
| H | 1.939257 | 3.021658 | -0.791734 |
| H | -0.324297 | 3.914728 | -0.322712 |
| H | -2.109595 | -1.354329 | -0.614182 |

**2-QuMeOH, dihydro tautomer**

| Atom | x | y | z |
|---|---|---|---|
| C | -6.278944 | 0.526726 | 0.637183 |
| C | -6.175859 | 1.798766 | 0.076099 |
| C | -4.926123 | 2.294894 | -0.298433 |
| C | -3.809883 | 1.503188 | -0.095773 |
| C | -3.873063 | 0.230801 | 0.473690 |
| C | -5.139387 | -0.249241 | 0.833901 |
| N | -2.489912 | 2.036375 | -0.532425 |
| C | -1.235511 | 1.419354 | 0.059292 |
| C | -1.439239 | 0.010875 | 0.554016 |
| C | -2.648864 | -0.526383 | 0.721750 |
| H | -7.056893 | 2.411443 | -0.069594 |
| H | -5.219105 | -1.234066 | 1.280241 |
| H | -7.248870 | 0.141478 | 0.928739 |
| C | -0.185933 | 1.412976 | -1.058330 |
| H | -4.839734 | 3.287641 | -0.725140 |
| O | -0.460712 | 1.727150 | -2.188838 |
| H | -2.480561 | 3.033116 | -0.302510 |
| H | 0.816522 | 1.071552 | -0.759412 |
| H | -0.883054 | 2.067503 | 0.869507 |
| H | -2.384347 | 1.978621 | -1.560948 |
| H | -0.534855 | -0.540178 | 0.783165 |
| H | -2.742913 | -1.542076 | 1.088991 |

**4-QuMeOH, starting alcohol**

| Atom | x | y | z |
|---|---|---|---|
| C | -3.781272 | 1.900603 | 0.173476 |
| C | -3.774268 | 0.487152 | -0.007670 |
| N | -2.589747 | 2.577539 | 0.060821 |
| C | -1.430292 | 1.980799 | -0.202086 |
| C | -1.380581 | 0.599264 | -0.377194 |
| C | -2.533261 | -0.160664 | -0.278719 |
| C | -2.446552 | -1.660770 | -0.437775 |
| O | -3.208691 | -2.044894 | -1.584614 |
| H | -3.205799 | -3.008580 | -1.639373 |
| C | -5.010254 | -0.199497 | 0.119199 |
| C | -6.165670 | 0.487724 | 0.406335 |
| C | -6.143711 | 1.890153 | 0.581313 |
| C | -4.967977 | 2.596347 | 0.469851 |
| H | -7.062453 | 2.417039 | 0.811512 |
| H | -4.950625 | 3.668959 | 0.620274 |
| H | -5.034868 | -1.269812 | -0.030497 |
| H | -7.101921 | -0.049389 | 0.498714 |
| H | -2.595070 | 3.585619 | 0.199765 |
| H | -1.397170 | -1.945477 | -0.548354 |
| H | -2.834710 | -2.140026 | 0.470019 |
| H | -0.423873 | 0.141938 | -0.590870 |
| H | -0.560145 | 2.619833 | -0.271995 |

**4-QuMeOH, enol tautomer**

| Atom | x | y | z |
|---|---|---|---|
| C | -3.669525 | 1.718073 | 0.043876 |
| C | -3.551378 | 0.401198 | -0.093385 |
| N | -2.518468 | 2.611847 | -0.215719 |
| C | -1.194063 | 1.956495 | -0.419004 |
| C | -1.106426 | 0.566989 | -0.557156 |
| C | -2.318492 | -0.267033 | -0.468621 |
| C | -2.310037 | -1.599635 | -0.706435 |
| O | -3.426410 | -2.345049 | -0.578639 |
| H | -2.438110 | 3.269243 | 0.568560 |
| H | -2.730604 | 3.202831 | -1.029106 |
| H | -4.560679 | 2.253024 | 0.334999 |
| H | -4.434141 | -0.199576 | 0.086819 |
| H | -3.233960 | -3.270609 | -0.775151 |
| H | -1.420055 | -2.131592 | -1.019935 |
| C | 0.189087 | 0.047694 | -0.740388 |
| C | 1.301866 | 0.874076 | -0.810649 |
| C | 1.169380 | 2.258526 | -0.681589 |
| C | -0.092743 | 2.802092 | -0.479068 |
| H | 0.332546 | -1.022465 | -0.817476 |
| H | 2.281908 | 0.435228 | -0.957163 |
| H | 2.035848 | 2.906378 | -0.729423 |
| H | -0.215915 | 3.873195 | -0.362359 |

**4-QuMeOH, dihydro tautomer**

| Atom | x | y | z |
|---|---|---|---|
| C | -6.218529 | 0.534128 | 0.708616 |
| C | -6.139996 | 1.826379 | 0.191433 |
| C | -4.915402 | 2.328522 | -0.245556 |
| C | -3.796778 | 1.513823 | -0.157911 |
| C | -3.842319 | 0.222027 | 0.358525 |
| C | -5.077825 | -0.261294 | 0.796087 |
| N | -2.484758 | 2.037174 | -0.631083 |
| C | -1.309751 | 1.491788 | 0.097354 |
| C | -1.366310 | 0.267314 | 0.589248 |
| C | -2.571926 | -0.610939 | 0.418250 |
| H | -7.023258 | 2.450230 | 0.132572 |
| H | -5.143024 | -1.260652 | 1.210907 |
| H | -7.169440 | 0.145483 | 1.053106 |
| C | -2.501217 | -1.392299 | -0.915244 |
| H | -4.842138 | 3.337553 | -0.634730 |
| O | -2.063911 | -0.911244 | -1.931323 |
| H | -2.474200 | 3.059589 | -0.541814 |
| H | -2.923605 | -2.411108 | -0.892356 |
| H | -2.643829 | -1.331398 | 1.234068 |
| H | -2.380554 | 1.835000 | -1.636273 |
| H | -0.499177 | -0.125306 | 1.106094 |
| H | -0.471122 | 2.167550 | 0.148971 |

**5-QuMeOH, starting alcohol**

| Atom | x | y | z |
|---|---|---|---|
| C | -3.815532 | 1.902253 | 0.177329 |
| C | -3.834095 | 0.496803 | -0.047228 |
| N | -2.668752 | 2.590637 | -0.136946 |
| C | -1.572924 | 2.022533 | -0.637780 |
| C | -1.553786 | 0.652266 | -0.888420 |
| C | -2.675357 | -0.099585 | -0.598357 |
| C | -5.074266 | -1.731056 | 0.132071 |
| O | -5.010224 | -2.042979 | -1.268441 |
| H | -4.959202 | -3.002523 | -1.359255 |
| C | -5.011299 | -0.234082 | 0.307636 |
| C | -6.079797 | 0.451640 | 0.841916 |
| C | -6.038666 | 1.850610 | 1.051011 |
| C | -4.919978 | 2.580047 | 0.726668 |
| H | -6.901891 | 2.349700 | 1.474715 |
| H | -4.874489 | 3.650657 | 0.887242 |
| H | -6.975988 | -0.091416 | 1.119910 |
| H | -2.659952 | 3.593489 | 0.029512 |
| H | -4.238399 | -2.205727 | 0.661068 |
| H | -6.004937 | -2.103182 | 0.568835 |
| H | -0.662184 | 0.199595 | -1.300095 |
| H | -0.724098 | 2.670944 | -0.809862 |
| H | -2.669758 | -1.162743 | -0.799390 |

**5-QuMeOH, enol tautomer**

| Atom | x | y | z |
|---|---|---|---|
| C | -3.717770 | 1.836851 | 0.106398 |
| C | -3.617695 | 0.510265 | -0.024758 |
| N | -2.593412 | 2.661391 | -0.408388 |
| C | -1.262345 | 1.984785 | -0.385357 |
| C | -1.245875 | 0.550615 | -0.588248 |
| C | -2.423162 | -0.153657 | -0.481445 |
| C | 0.361174 | -1.334076 | -1.079015 |
| O | -0.585206 | -2.260576 | -1.233794 |
| H | -2.515440 | 3.523181 | 0.136042 |
| H | -2.823677 | 2.941646 | -1.371585 |
| H | -4.570602 | 2.399605 | 0.449388 |
| H | -4.468768 | -0.090276 | 0.275163 |
| H | -0.193572 | -3.122771 | -1.430821 |
| H | 1.390508 | -1.656513 | -1.193464 |
| C | 0.095208 | -0.020813 | -0.789702 |
| C | 1.251951 | 0.836175 | -0.643491 |
| C | 1.145641 | 2.156991 | -0.353364 |
| C | -0.155572 | 2.749965 | -0.238948 |
| H | 2.230716 | 0.385696 | -0.769295 |
| H | 2.023753 | 2.777958 | -0.233883 |
| H | -0.246080 | 3.816810 | -0.065788 |
| H | -2.444546 | -1.222850 | -0.610592 |

**5-QuMeOH, dihydro tautomer**

| Atom | x | y | z |
|---|---|---|---|
| C | -6.237253 | 0.604704 | 0.631325 |
| C | -6.069353 | 1.897485 | 0.313771 |
| C | -4.783236 | 2.395932 | -0.147487 |
| C | -3.712744 | 1.581553 | -0.144769 |
| C | -3.747138 | 0.221300 | 0.355017 |
| C | -5.115832 | -0.403821 | 0.498195 |
| N | -2.407287 | 2.063464 | -0.669020 |
| C | -1.207836 | 1.482478 | -0.002882 |
| C | -1.325706 | 0.302244 | 0.600516 |
| C | -2.595849 | -0.397308 | 0.703031 |
| H | -6.892517 | 2.597599 | 0.391243 |
| H | -7.203724 | 0.235291 | 0.955183 |
| C | -5.483311 | -1.334735 | -0.678710 |
| H | -4.706649 | 3.423172 | -0.486063 |
| O | -4.766178 | -1.580958 | -1.614541 |
| H | -2.363564 | 3.083989 | -0.590800 |
| H | -6.494101 | -1.776954 | -0.595215 |
| H | -5.133644 | -1.064062 | 1.376047 |
| H | -2.349067 | 1.852557 | -1.676615 |
| H | -0.440005 | -0.134907 | 1.046103 |
| H | -0.315691 | 2.075914 | -0.125535 |
| H | -2.604219 | -1.395324 | 1.121080 |

**7-QuMeOH, starting alcohol**

| Atom | x | y | z |
|---|---|---|---|
| C | -3.860008 | 1.902515 | 0.143611 |
| C | -3.815140 | 0.491402 | -0.032583 |
| N | -2.739352 | 2.622041 | -0.193364 |
| C | -1.624063 | 2.075477 | -0.682529 |
| C | -1.553298 | 0.700630 | -0.892942 |
| C | -2.642531 | -0.085104 | -0.562895 |
| C | -7.344335 | 2.414971 | 1.568257 |
| O | -7.215670 | 3.830903 | 1.579353 |
| H | -8.038615 | 4.210928 | 1.906580 |
| C | -4.960861 | -0.260457 | 0.341087 |
| C | -6.064620 | 0.371796 | 0.849674 |
| C | -6.096715 | 1.785400 | 1.007729 |
| C | -5.000654 | 2.544078 | 0.655800 |
| H | -5.022793 | 3.620299 | 0.766154 |
| H | -6.933386 | -0.210140 | 1.137323 |
| H | -2.757785 | 3.627528 | -0.045537 |
| H | -8.201645 | 2.103503 | 0.957742 |
| H | -7.506953 | 2.027783 | 2.583165 |
| H | -0.648308 | 0.272135 | -1.299564 |
| H | -0.800884 | 2.749081 | -0.882337 |
| H | -2.594837 | -1.159598 | -0.700549 |
| H | -4.943016 | -1.336391 | 0.214786 |

**7-QuMeOH, enol tautomer**

| Atom | x | y | z |
|---|---|---|---|
| C | -3.633485 | 1.917186 | 0.147925 |
| C | -3.570921 | 0.583952 | 0.051380 |
| N | -2.530643 | 2.722697 | -0.445222 |
| C | -1.223354 | 2.011005 | -0.581570 |
| C | -1.263112 | 0.570427 | -0.714521 |
| C | -2.423585 | -0.115988 | -0.468621 |
| C | 2.310075 | 2.846083 | -0.919982 |
| O | 3.513266 | 2.306702 | -1.139983 |
| H | -2.380283 | 3.568618 | 0.110431 |
| H | -2.833367 | 3.044765 | -1.373734 |
| H | -4.439795 | 2.509771 | 0.549997 |
| H | -4.412704 | 0.014343 | 0.428718 |
| H | 4.197283 | 2.988812 | -1.134769 |
| H | 2.267691 | 3.919764 | -0.769813 |
| C | 0.005442 | -0.055742 | -1.021331 |
| C | 1.157312 | 0.653833 | -1.097070 |
| C | 1.174146 | 2.085881 | -0.885710 |
| C | -0.087336 | 2.736223 | -0.634656 |
| H | 2.092971 | 0.151429 | -1.309480 |
| H | -0.112762 | 3.814473 | -0.514430 |
| H | -2.451385 | -1.194668 | -0.553800 |
| H | 0.015657 | -1.130625 | -1.162798 |

**7-QuMeOH, dihydro tautomer**

| **Atom** | **x** | **y** | **z** |
|---|---|---|---|
| C | -6.148685 | 0.492675 | 0.459448 |
| C | -6.102749 | 1.982937 | 0.216918 |
| C | -4.721196 | 2.489926 | -0.085018 |
| C | -3.665350 | 1.676203 | -0.052869 |
| C | -3.717715 | 0.267124 | 0.316641 |
| C | -5.053158 | -0.280513 | 0.493858 |
| N | -2.334621 | 2.168569 | -0.506180 |
| C | -1.160676 | 1.538362 | 0.159014 |
| C | -1.296950 | 0.285860 | 0.595268 |
| C | -2.568684 | -0.411334 | 0.571882 |
| H | -7.128304 | 0.062023 | 0.634816 |
| C | -7.074435 | 2.313397 | -0.931594 |
| H | -4.610833 | 3.536116 | -0.351926 |
| O | -8.229227 | 2.610914 | -0.748438 |
| H | -2.288759 | 3.188757 | -0.414264 |
| H | -6.655958 | 2.232960 | -1.952170 |
| H | -6.501089 | 2.504961 | 1.098189 |
| H | -2.257591 | 1.972137 | -1.514855 |
| H | -0.430718 | -0.206442 | 1.020029 |
| H | -0.273825 | 2.150717 | 0.170359 |
| H | -2.602527 | -1.453048 | 0.865310 |
| H | -5.136970 | -1.340437 | 0.707666 |
